# Synthesis of Indole Derivatives via Aryl Triazole Ring-Opening and Subsequent Cyclization

**DOI:** 10.3390/molecules30020337

**Published:** 2025-01-16

**Authors:** Aleksejs Burcevs, Armands Sebris, Irina Novosjolova, Anatoly Mishnev, Māris Turks

**Affiliations:** 1 Institute of Chemistry and Chemical Technology, Faculty of Natural Sciences and Technology, Riga Technical University, P. Valdena Str. 3, LV-1048 Riga, Latvia; 2 Latvian Institute of Organic Synthesis, Aizkraukles Str. 21, LV-1006 Riga, Latvia

**Keywords:** indoles, purines, triazole ring-opening, indole synthesis, metal-free approach

## Abstract

A metal-free two-step synthetic approach for obtaining indole derivatives from aryl triazole fragment-containing compounds has been developed. In the first step, the Dimroth equilibrium, followed by nitrogen extrusion, Wolff rearrangement, and amine nucleophile addition, leads to the formation of *N*-aryl ethene-1,1-diamines. In the second step, the latter intermediates are cyclized into the target 1*H*-indoles in the presence of iodine. The developed method ensures the synthesis of indoles that possess *N*-substituents at the indole *C*2 position. Depending on the applied *N*-nucleophile, the indolization step provides a selectivity either towards 1*H*-indoles or 1-aryl-1*H*-indoles.

## 1. Introduction

Both purine and indole are heterocycles that are abundant in nature and have a wide array of biological activities and thus, are important to be studied for their applications in medicinal chemistry [1,2,3,4]. Since many compounds in these substance classes possess fluorescent properties, they are also used as probes for studying many biological processes in cells [5,6,7,8]. A logic junction of both structural motifs has led to the synthesis of purine–indole conjugates to test their combined properties. There have been multiple reports from different research groups on purine–indole conjugates as fluorescent probes for various processes in organism, such as the NarI recognition sequence [9], the protein-mediated duplex to G-quadruplex exchange [10], and Hoogsteen base pairing [11]. Purine–indole conjugates possessing antitumor properties [12] or regulating lipid metabolism [13] have also been reported.

Purine–indole conjugates can be synthesized using multiple methods, with the most common method being Pd catalysed cross-coupling between selected fragments of indole and purine [9,11,13,14]. There have also been reports of the transition metal free synthesis of purine–indole conjugates by the direct arylation of purine *C*6 position using AlCl_3_ [15] or a TfOH and HFIP combination [16]. Nevertheless, the latter methods are limited to a specific purine position and substituents at the aromatic ring, which is being attached to the purine core.

**Scheme 1:**
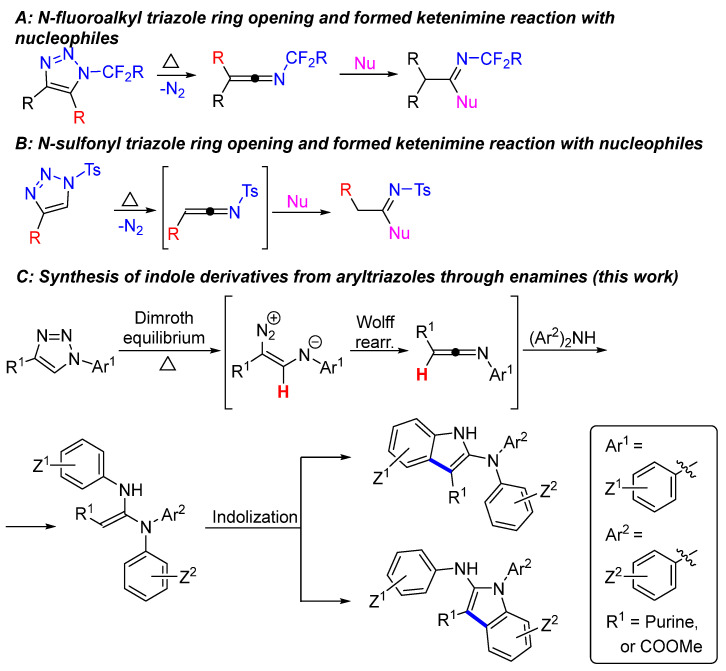
(**A**) *N*-fluoroalkyl triazole ring-opening and subsequent reactions. (**B**) *N*-sulfonyl triazole ring-opening and subsequent reactions. (**C**) Synthesis of indole derivatives from aryl triazoles (this work).

On the other hand, chemistry leading to purine–triazole conjugates has been well developed. Among others [17,18,19,20,21,22,23,24,25,26], we have also contributed to both C–C and C–N linked triazolyl purines and have demonstrated their further synthetic applications in S_N_Ar reactions [27,28,29,30], as well as their use as metal ion sensors [31] and fluorescent materials [32,33,34]. We have envisaged that the triazole ring-opening in a C–C-linked triazolyl purine via the sequential Dimroth equilibrium and Wolff rearrangement should provide a ketenimine intermediate, which upon amine addition, will result in ethene-1,1-diamine. The latter, in turn, can be cyclized to indole if an *N*-aryl substituent is available (Scheme 1).

The triazole ring-opening reactions that are required for our planned sequence are known in the literature. At high temperatures using flash vacuum pyrolysis conditions (400−800 °C), the triazole ring opens with the formation of carbene species that further rearrange into various products [35,36,37,38]. Other examples involve the use of transition metal catalysis at more ordinary temperatures, yet most of these methods are developed for triazoles bearing a *N*-tosyl group or other similar electron-withdrawing substituents. These methods are mostly developed for rhodium catalysis [39], and the formed metal-stabilized carbene can be used to form new five- or six-membered heterocycles [40,41]. Other metals, such as Ag [42,43], Cu [44], and Ni [45], have also been used in triazole ring-opening.

Nevertheless, evidence for triazole ring-opening at ordinary temperatures and in the absence of transition metals is present in the literature. By using 1-fluoroalkyl-1*H*-triazole, the ring-opening and formation of ketenimine occurs under metal-free conditions at 130−160 °C [46] (Scheme 1A). Next, the formed intermediate can react with *N*-, *S*-, or *O*-nucleophiles with the formation of amidines, thioimidates, or iminoesters. Also, *N*-sulfonyltriazoles can be opened similarly [42] (Scheme 1B).

Hence, we report a synthetic sequence which involves a metal-free triazole ring-opening of C–C-linked purine triazole conjugates, an amine addition to the intermediate ketenimine, and an oxidative cyclization of the resulting ethene-1,1-diamines to indoles. (Scheme 1C).

## 2. Results and Discussion

**Scheme 2:**
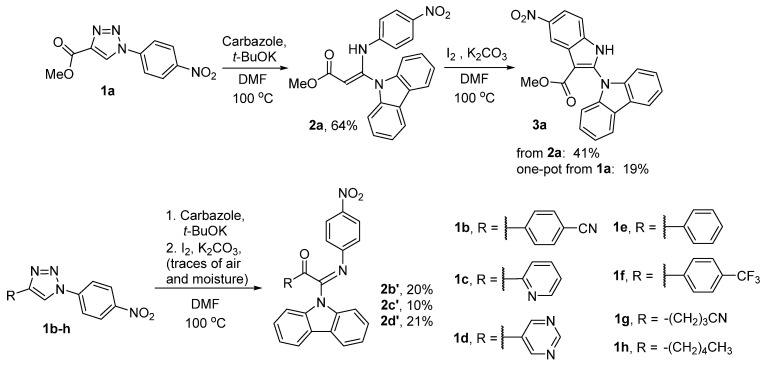
Model studies for 1*H*-indole synthesis from triazoles **1a**–**h**.

We started our research with attempts to open model aryl triazoles **1** [47,48,49,50,51] with carbazole. Carbazole nucleophile was chosen due to its extensive use in fluorescent materials [52] and its presence in both biologically active natural alkaloids [53] and numerous drugs [54]. We found that such transformation was possible by using *t*-BuOK and DMF at 100 °C (Scheme 2), and only in the case of starting material **1a**, we observed the formation of ethene-1,1-diamine **2a**, which could be isolated. With the model ethene-1,1-diamine **2a** in hand, we proceeded to its cyclization into indole. There are several examples of similar known transformations that use Pd, Cu, and Fe catalysts [55,56,57,58,59]. We have unsuccessfully explored combinations of Pd(OAc)_2_/Bu_4_NBr/O_2_ [55], CuI/Li_2_CO_3_/phen [56], and CuCl_2_/K_3_PO_4_ [58] as the reagent systems for the substrate **2a** cyclization. Then, we switched to the screening of metal-free methods that involved the use of CBr_4_/K_2_CO_3_ [60], PIDA [61], and I_2_/K_2_CO_3_ [62]. To our delight, we have found that the I_2_/K_2_CO_3_ reagent system led to the formation of envisaged indole **3a**. For the given sequence, a step-wise process provided better yield than the tested one-pot approach.

**Figure 1:**
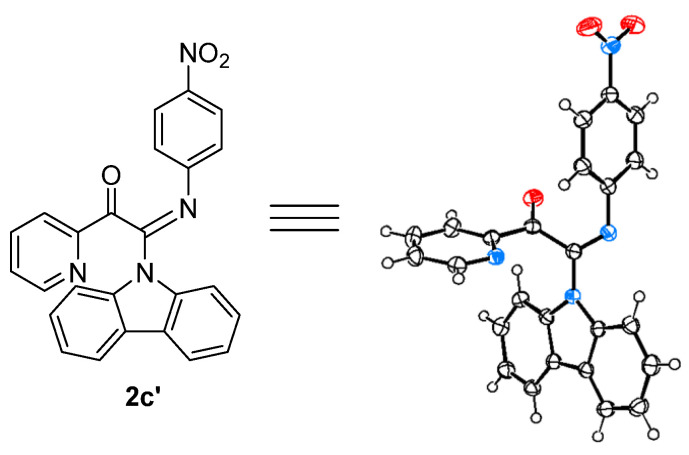
X-ray crystallographic analysis of compound **2c’**.

On the other hand, triazoles **1b**–**d**, which contained weaker electron-withdrawing substituents such as cyanophenyl, pyridyl, or pyrimidinyl groups, provided ethene-1,1-diamine intermediates **2b**–**d,** which appeared to be incompatible with standard isolation and purification approaches. Therefore, the one-pot approach was applied, which implied the direct addition of I_2_/K_2_CO_3_ to the reaction mixture after the first triazole ring-opening/carbazole addition step. In this case, we observed only the formation of acyclic oxidation products of type **2b′**–**d′**, whose structures were elucidated by standard spectroscopic methods and unambiguously proved by single crystal X-ray analysis, in the case of product **2c′** (Figure 1). Compounds **1e**–**h**, bearing other sidechains, including aliphatic substituents, underwent rapid degradation of their starting material into various unidentifiable mixtures of products upon attempted triazole ring-opening reactions.

**Scheme 3:**
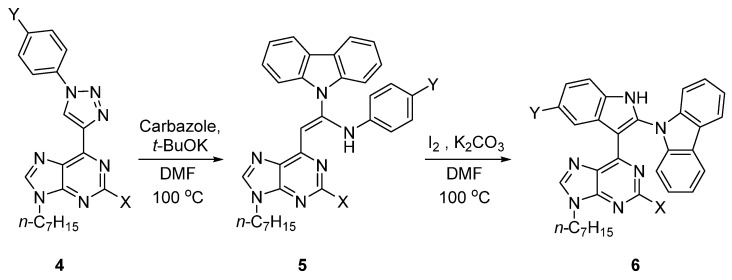
Synthesis of purine–indole–carbazole conjugates **6**.

Next, we applied the developed synthetic sequence to triazolyl purines **4**, creating aromatic ethene-1,1-diamines **5** that we subsequently cyclized into 1*H*-indole derivatives **6**. Both a stepwise sequence and a one-pot approach were tested (Scheme 3 and Table 1). In the reaction, we used triazolyl purines **4**, with or without a chlorine substituent at the purine *C*2 position. In the case of the dimethylamino group containing derivative **4e**, it was only possible to obtain ethene-1,1-diamine **5e** by lowering the temperature, yet this did not provide indole in the next step. In all cases, ethene-1,1-diamines **5** were formed as the only products. The total yields of the final products **6** are either significantly higher (Table 1, Entries 1, 4) or just slightly lower (Table 1, Entries 2, 3) in the case of one-pot reactions when compared to those obtained using the stepwise approach. After that, we decided to derivatize the starting material further and introduced another phenyl triazole moiety at the purine C2 position. Thus, we synthesized starting material **4h** and applied standard triazole ring-opening conditions using carbazole as a nucleophile. The resulting product was compound **5h**, with only one triazole being opened at the purine C6 position (Table 1, Entry 8).

We can conclude that triazole ring-opening—carbazole addition proceeds with purine–triazole conjugates containing aryl groups with either electron-donating or electron-withdrawing substituents. On the other hand, further cyclization to indole is not possible with the dimethylaminophenyl substituent under the given reaction conditions (Entry 5). Also, it is evident that at least one sufficiently strong electron-withdrawing substituent (e.g., purine or a COOMe group) should be present at the triazole ring.

In transformations from **4** to **5**, another side reaction is theoretically possible. In the presence of a base, carbazole could provide the S_N_Ar product with the used 2-chloropurine starting materials (Entries 1–5). However, the observed results prove that the Dimroth–Wolff cascade, followed by *N*-nucleophile addition, is proceeding at a higher rate than the S_N_Ar process. It is worth mentioning that 5-nitro-indole derivative **6b** was successfully synthesized using our approach, while such a substitution pattern may provide synthetic difficulties for other methodologies [16].

**Figure 2:**
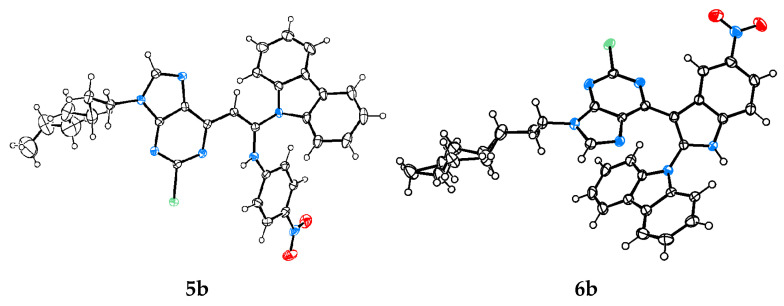
X-ray crystallographic analyses of compounds **5b** and **6b**.

**Figure 3:**
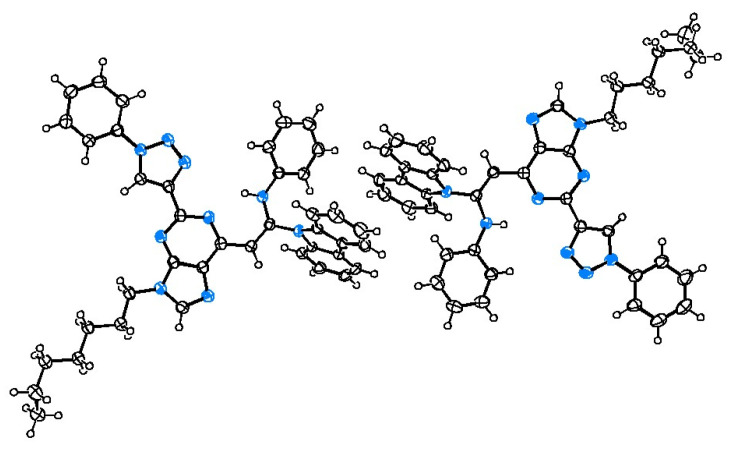
X-ray crystallographic analysis of compound **5h**.

In order to prove the double bond configuration of the formed ethene-1,1-diamines and to verify the molecular structure of the target purine–indole conjugate, single crystal X-ray crystallographic analysis was performed for compounds **5b** and **6b** (Figure 2). On the other hand, X-ray analysis of the mono triazole ring-opened product **5h** proved the reaction selectivity for the triazole ring at the purine C6 position (Figure 3).

**Scheme 4:**
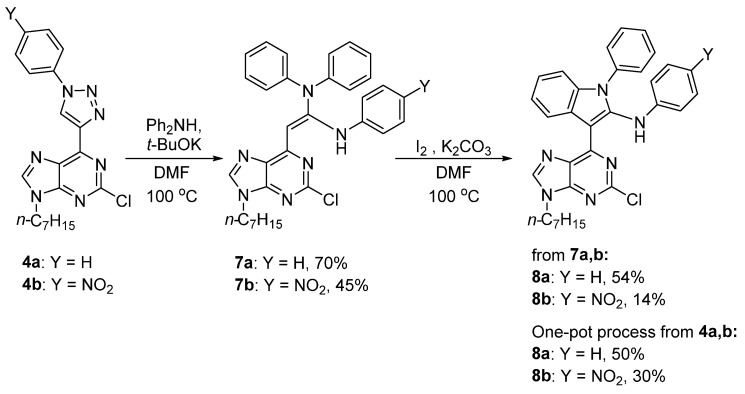
Synthesis of *N*-phenyl-containing indole derivatives **8** from triazolyl purines **4**.

Next, we have explored our methodology through the use of diphenylamine, applying the previously developed conditions to starting materials **4a** and **4b**. The expected ethene-1,1-diamines **7a**,**b** were obtained in 70% and 45% yields, respectively. Interestingly, products **7a**,**b** underwent the cyclization at the phenyl group of the incoming diphenylamine nucleophile. Thus, the formation of *N*-phenyl indole products **8** was observed (Scheme 4). As with previous reactions, the total yields of one-pot synthesis were proven to be higher than those obtained using the same reactions in a stepwise approach.

**Figure 4:**
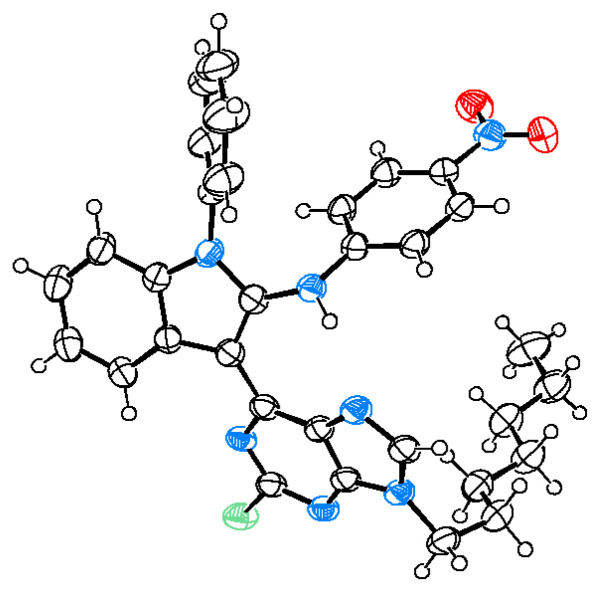
X-ray crystallographic analysis of compound **8b**.

The chemical structure of products **8a**,**b**, which featured changed cyclization regioselectivity, were established by standard spectroscopic techniques and were additionally proved by crystallographic analysis, in the case of compound **8b** (Figure 4).

**Scheme 5:**
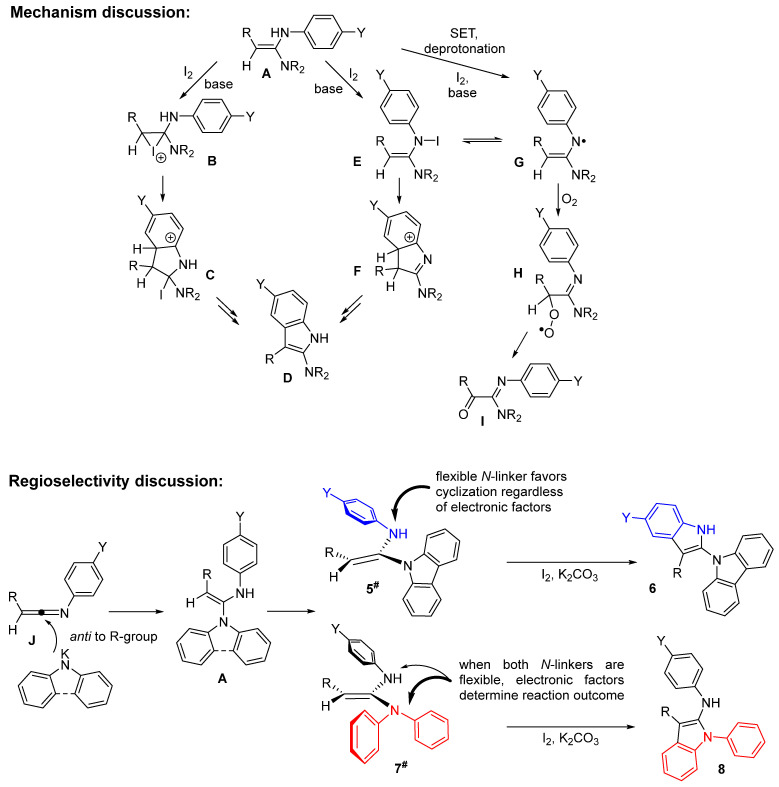
Proposed reaction mechanism and regioselectivity model for indolization of ethene-1,1-diamines **A**.

Cyclization of compound **7a**, which contained three phenyl rings, without any substituents, and formed selectively product **8a**, proves that steric hindrance or linker flexibility plays a more important role than do the electronic effects (Scheme 5). Thus, in the cyclization **5**→**6**, which is featured by conformation **5^#^** (Scheme 5) arylamine moiety, with a flexible NH-linker, ensures cyclization, even of such electron-deficient substituents as the nitrophenyl or cyanophenyl groups (formation of compounds **6b**, **6d**, **6g**, Table 1). On the other hand, when both *N*-linkers are equally flexible, which is featured by conformation **7^#^** (Scheme 5) and cyclization **7**→**8**, both the statistical accessibility of the aryl group and their electronic density determined the reaction outcome, i.e., cyclization occurred at the phenyl ring instead of the nitrophenyl ring (formation of product **8b**, Scheme 4).

Based on the observed product and byproduct formation, we propose reaction mechanisms for both indole **3**, **6**, and **8,** as well as acyclic compound **2′** formation. For the indole formation, two distinct pathways are plausible: (1) **A→B→C→D** [62], and (2) **A→E→F→D**, which differ regarding the electrophilic iodine attachment site. However, the pathway **A→E→F→D** better explains the observed reaction outcomes, depending on the electronic nature of the substituents, and permits the coupling of the proposed early intermediate **E** with the side product formation pathway **E→G→H→I**. Thus, iodoamine **E** formation can occur either via a nucleophilic attack of arylamine moiety or via a SET process [63,64,65]. The rate and efficiency of the cyclization of **E**→**F** most probably depends on electron density in the ethene-1,1-diamine system, for which the electron-withdrawing substituents R (-COOMe or purine) favor the attack of the aromatic ring as π-nucleophile. On the other hand, the electron-richer ethene-1,1-diamine systems meet with difficulties in accepting the incoming π-nucleophile. Therefore, intermediate **E** can engage in homolytic N-I bond cleavage and form acyclic oxidation products of type **I** in the presence of oxygen traces [66].

## 3. Materials and Methods

### 3.1. General Information

Compounds **1a**–**c, 1e**–**f,** and **1h** were prepared according to the procedures outlined in the literature [47,48,49,50,51,67]. Compounds **1d** and **1g** were synthesized in a similar way as was **1a**–**c**, and their characterization is found in the Supplementary Materials. Compounds **4d**–**h** were synthesized using known procedures [32], and their descriptions and characterization are found in the Supplementary Materials.

Crystallographic data for **2c′**, **5b**, **5h**, **6b**, and **8b** have been deposited with the Cambridge Crystallographic Data Center as supplementary publications No. CCDC-2391861, CCDC-2391852, CCDC-2391858, CCDC-2391859, and CCDC-2392586, respectively.

### 3.2. General Methods

General method **A** for triazole ring-opening: in an inert atmosphere, to a mixture of triazole derivative (1.0 eq.) in dry DMF (1 mL/0.15 mmol of triazole derivative), KO*t*Bu (1.1 eq.) and nucleophile (1.1 eq.) were added, and the reaction mixture was stirred at a temperature of 100 °C for 1 h. Then, it was evaporated and dried in a vacuum. The resulting solid was suspended in DCM (20 mL) and washed with 5% aqueous LiCl solution (2 × 20 mL) and brine (20 mL). The organic phase was dried over anhydrous Na_2_SO_4_, filtered, and evaporated, followed by silica gel column chromatography.

General method **B** for cyclization into indole: in an inert atmosphere, to a mixture of enamine derivative (1.0 eq.) in dry DMF (1 mL/0.05 mmol of enamine derivative), K_2_CO_3_ (1.1 eq.) and I_2_ (1.2 eq.) were added, and the reaction mixture was stirred at a temperature of 100 °C for 1 h. Then, it was evaporated and dried in a vacuum. The resulting solid was suspended in DCM (20 mL) and washed with 5% aqueous LiCl solution (2 × 20 mL), 20% aqueous Na_2_S_2_O_3_·5H_2_O solution (20 mL), and brine (20 mL). The organic phase was dried over anhydrous Na_2_SO_4_, filtered, and evaporated, followed by silica gel column chromatography.

General method **C** for one-pot reactions: in an inert atmosphere, to a mixture of triazole derivative (1.0 eq.) in dry DMF (1 mL/0.10 mmol of triazole derivative), KO*t*Bu (1.1 eq.) and nucleophile (1.1 eq.) were added, and the reaction mixture was stirred at a temperature 100 °C for 1 h. Then, K_2_CO_3_ (1.2 eq.) and I_2_ (1.2 eq.) were quickly added, and the reaction mixture was stirred at a temperature of 100 °C for 1 h. Then, it was evaporated and dried in a vacuum. The resulting solid was suspended in DCM (20 mL) and washed with 5% aqueous LiCl solution (2 × 20 mL), 20% aqueous Na_2_S_2_O_3_·5H_2_O solution (20 mL), and brine (20 mL). The organic phase was dried over anhydrous Na_2_SO_4_, filtered, and evaporated, followed by silica gel column chromatography.

### 3.3. Characterization Data for Products

Methyl (*E*)-3-(9*H*-carbazol-9-yl)-3-[(4-nitrophenyl)amino]acrylate (**2a**)

Method **A**, 250 mg of **1a**, with carbazole as the nucleophile. Silica gel column chromatography (Hex/DCM, gradient 0%→100%) provided product **2a** (yield: 252 mg, 64%) as an orange amorphous solid. R_f_ = 0.67 (DCM = 100%). HPLC: t*_R_* = 7.23 min, eluent E_2_. IR (neat) ν (cm^−1^): 1659, 1635, 1588, 1479, 1327, 1253, 1205, 1159, 1109, 1046, 844, 741. ^1^H-NMR (500 MHz, CDCl_3_) δ (ppm): 10.53 (s, 1H, (-NH)), 8.03 (d, 2H, ^3^*J* = 7.9 Hz, 2 × H-C(carbazole)), 7.77 (d, 2H, ^3^*J* = 9.1 Hz, 2 × H-C(Ar)), 7.57 (d, 2H, ^3^*J* = 7.9 Hz, 2 × H-C(carbazole)), 7.38 (t, 2H, ^3^*J* = 7.9 Hz, 2 × H-C(carbazole)), 7.29 (t, 2H, ^3^*J* = 7.9 Hz, 2 × H-C(carbazole)), 6.54 (d, 2H, ^3^*J* = 9.1 Hz, 2 × H-C(Ar)), 5.39 (s, 1H, H-C(vinyl)), 3.83 (s, 3H, (-OMe)). ^13^C-NMR (126 MHz, CDCl_3_) δ (ppm): 170.5, 147.7, 144.8, 142.8, 138.3, 126.9, 125.3, 124.5, 121.9, 120.6, 118.4, 111.5, 90.1, 51.5. HRMS (ESI) *m*/*z*: [M − H]^−^ Calcd for C_22_H_16_N_3_O_4_ 386.1146; Found 386.1149 (0.78 ppm).

(*E*)-4-(2-(9*H*-Carbazol-9-yl)-2-((4-nitrophenyl)imino)acetyl)benzonitrile (**2b′**)

Method **C**, 150 mg of **1b**, with carbazole as the nucleophile. Silica gel column chromatography (DCM = 100%) provided product **2b′** (yield: 46 mg, 20%) as an orange amorphous solid. R_f_ = 0.70 (DCM = 100%). HPLC: t*_R_* = 7.34 min, eluent E_2_. IR (neat) ν (cm^−1^): 2227, 1679, 1610, 1582, 1508, 1445, 1381, 1332, 1222, 1206, 1163, 1103, 854, 751, 721. ^1^H-NMR (500 MHz, CDCl_3_) δ (ppm): 8.09–8.04 (m, 4H, 2 × H-C(Ar-NO_2_), 2 × H-C(carbazole)), 7.93–7.86 (m, 4H, 2 × H-C(Ar-CN), 2 × H-C(carbazole)), 7.68 (d, 2H, ^3^*J* = 8.2 Hz, 2 × H-C(Ar-CN)), 7.41 (t, 2H, ^3^*J* = 7.7 Hz, 2 × H-C(carbazole)), 7.37 (t, 2H, ^3^*J* = 7.7 Hz, 2 × H-C(carbazole)), 6.95 (d, 2H, ^3^*J* = 8.7 Hz, 2 × H-C(Ar-NO_2_). ^13^C-NMR (126 MHz, CDCl_3_) δ (ppm): 188.3, 152.0, 144.3, 138.1, 137.0, 133.4, 129.6, 127.6, 127.5, 126.4, 124.9, 124.2, 121.6, 120.6, 118.7, 117.2, 114.9. HRMS (ESI) *m*/*z*: [M + H]^+^ Calcd for C_27_H_17_N_4_O_3_ 445.1295; Found 445.1297 (0.45 ppm).

(*E*)-2-(9*H*-Carbazol-9-yl)-2-((4-nitrophenyl)imino)-1-(pyridin-2-yl)ethan-1-one (**2c′**)

Method **C**, 200 mg of **1c**, with carbazole as the nucleophile. Silica gel column chromatography (DCM/EtOH, gradient 0%→2%) provided product **2c′** (yield: 31 mg, 10%) as an orange solid. m_p_ = 201–202 °C (crystallized from DCM:Hex = 1:40). R_f_ = 0.70 (DCM/MeCN = 20:1). HPLC: t*_R_* = 7.32 min, eluent E_2_. IR (neat) ν (cm^−1^): 1698, 1581, 1505, 1443, 1380, 1328, 1221, 1204, 1166, 1104, 1007, 939. ^1^H-NMR (500 MHz, CDCl_3_) δ (ppm): 8.58–8.54 (m, 1H, H-C(Pyr)), 8.06–8.02 (m, 2H, 2 × H-C(carbazole)), 7.99 (d, 2H, ^3^*J* = 8.4 Hz, 2 × H-C(Ar)), 7.94–7.90 (m, 3H, H-C(Pyr), 2 × H-C(carbazole)), 7.82–7.75 (m, 1H, H-C(Pyr)), 7.44–7.40 (m, 1H, H-C(Pyr)), 7.38–7.32 (m, 4H, 4 × H-C(carbazole)), 6.96 (d, 2H, ^3^*J* = 8.4 Hz, 2 × H-C(Ar)). ^13^C-NMR (126 MHz, CDCl_3_) δ (ppm): 190.4, 153.1, 152.2, 151.7, 150.1, 143.9, 138.7, 137.7, 128.6, 127.1, 126.2, 124.5, 123.5, 123.4, 121.5, 120.2, 114.8. HRMS (ESI) *m*/*z*: [M + H]^+^ Calcd for C_25_H_17_N_4_O_3_ 421.1295; Found 421.1291 (0.95 ppm).

(*E*)-2-(9*H*-Carbazol-9-yl)-2-((4-nitrophenyl)imino)-1-(pyrimidin-5-yl)ethan-1-one (**2d′**)

Method **C**, 150 mg of **1d**, with carbazole as the nucleophile. Silica gel column chromatography (Hex/EtOAc, gradient 0%→50%) provided product **2d′** (yield: 49 mg, 21%) as a yellow amorphous solid. R_f_ = 0.80 (Hex/EtOAc = 1:1). HPLC: t*_R_* = 6.84 min, eluent E_2_. IR (neat) ν (cm^−1^): 1683, 1624, 1583, 1574, 1510, 1445, 1332, 1310, 1223, 1100, 932, 873, 751, 723, 677. ^1^H-NMR (500 MHz, CDCl_3_) δ (ppm): 9.31 (s, 1H, H-C(pyrimidine)), 9.05 (s, 2H, 2 × H-C(pyrimidine)), 8.09 (d, 2H, ^3^*J* = 8.4 Hz, 2 × H-C(Ar)), 8.07–8.03 (m, 2H, 2 × H-C(carbazole)), 7.95–7.83 (m, 2H, 2 × H-C(carbazole)), 7.49–7.34 (m, 4H, 4 × H-C(carbazole)), 6.96 (d, 2H, ^3^*J* = 8.4 Hz, 2 × H-C(Ar)). ^13^C-NMR (126 MHz, CDCl_3_) δ (ppm): 187.0, 162.7, 157.6, 151.7, 149.4, 144.5, 137.9, 127.7, 127.6, 126.4, 125.1, 124.4, 121.5, 120.7, 114.6. HRMS (ESI) *m*/*z*: [M + H]^+^ Calcd for C_24_H_16_N_5_O_3_ 422.1248; Found 422.1246 (0.47 ppm).

Methyl 2-(9*H*-carbazol-9-yl)-5-nitro-1*H*-indole-3-carboxylate (**3a**)

Method **B**, 75 mg of **2a**; silica gel column chromatography (DCM = 100%) provided product **3a** (yield: 31 mg, 41%) as an orange amorphous solid. R_f_ = 0.39 (DCM = 100%). HPLC: t*_R_* = 6.45 min, eluent E_2_. IR (neat) ν (cm^−1^): 3203, 1670, 1520, 1471, 1340, 1227, 1200, 1096, 787, 740, 720. ^1^H-NMR (500 MHz, CDCl_3_) δ (ppm): 13.52 (s, 1H, (-NH)), 9.01 (d, 1H, ^4^*J* = 2.4 Hz, H-C(indole)), 8.28 (d, 2H, ^3^*J* = 7.7 Hz, 2 × H-C(carbazole)), 8.24 (dd, 1H, ^3^*J* = 9.0 Hz, ^4^*J* = 2.4 Hz, H-C(indole)), 7.71 (d, 1H, ^3^*J* = 9.0 Hz, H-C(indole)), 7.46 (t, 2H, ^3^*J* = 7.7 Hz, 2 × H-C(carbazole)), 7.38–7.31 (m, 4H, 4 × H-C(carbazole)), 3.56 (s, 3H, (-OMe)). ^13^C-NMR (126 MHz, CDCl_3_) δ (ppm): 162.6, 142.7, 140.2, 138.6, 136.9, 126.6, 125.2, 123.3, 121.2, 120.6, 118.8, 117.6, 113.2, 110.8, 102.7, 51.1. HRMS (ESI) *m*/*z*: [M − H]^−^ Calcd for C_22_H_14_N_3_O_4_ 384.0990; Found 384.0990.

Method **C**, 100 mg of **1a**, with carbazole as the nucleophile. Silica gel column chromatography (DCM = 100%) provided product **3a** (yield: 30 mg, 19%) as an orange amorphous solid.

(*E*)-*N*-[1-(9*H*-Carbazol-9-yl)-2-(2-chloro-9-heptyl-9*H*-purin-6-yl)vinyl]aniline (**5a**)

Method **A**, 300 mg of **4a** [32], with carbazole as the nucleophile. Silica gel column chromatography (DCM = 100%) provided product **5a** (yield: 254 mg, 63%) as a yellow amorphous solid. R_f_ = 0.52 (DCM = 100%). HPLC: t*_R_* = 10.36 min, eluent E_1_. IR (neat) ν (cm^−1^): 2925, 2854, 1632, 1575, 1553, 1440, 1309, 1250, 1224, 981, 746, 722. ^1^H-NMR (500 MHz, CDCl_3_) δ (ppm): 12.03 (s, 1H, -NH), 8.05 (d, 2H, ^3^*J* = 8.3 Hz, 2 × H-C(carbazole)), 7.87 (s, 1H, H-C(purine)), 7.68 (d, 2H, ^3^*J* = 8.3 Hz, 2 × H-C(carbazole)), 7.38 (t, 2H, ^3^*J* = 8.3 Hz, 2 × H-C(carbazole)), 7.27 (t, 2H, ^3^*J* = 8.3 Hz, 2 × H-C(carbazole)), 6.98 (t, 2H, ^3^*J* = 7.8 Hz, 2 × H-C(Ar)), 6.82 (t, 1H, ^3^*J* = 7.8 Hz, H-C(Ar)), 6.69 (d, 2H, ^3^*J* = 7.8 Hz, 2 × H-C(Ar)), 6.35 (s, 1H, H-C(vinyl)), 4.19 (t, 2H, ^3^*J* = 6.8 Hz, (-CH_2_-)), 1.94–1.87 (m, 2H, (-CH_2_-)), 1.36–1.20 (m, 8H, 4 × (-CH_2_-)), 0.89 (t, 3H, ^3^*J* = 6.8 Hz, (-CH_3_)). ^13^C-NMR (126 MHz, CDCl_3_) δ (ppm): 157.2, 152.9, 151.2, 146.1, 142.6, 139.2 (2C), 129.3, 128.0, 126.7, 124.4, 123.8, 121.3, 120.3, 119.8, 112.0, 90.0, 44.1, 31.7, 30.1, 28.8, 26.7, 22.7, 14.2. HRMS (ESI) *m*/*z*: [M + H]^+^ Calcd for C_32_H_32_ClN_6_ 535.2371; Found 535.2369 (0.37 ppm).

(*E*)-*N*-[1-(9*H*-Carbazol-9-yl)-2-(2-chloro-9-heptyl-9*H*-purin-6-yl)vinyl]-4-nitroaniline (**5b**)

Method **A**, 750 mg of **4b** [32], with carbazole as the nucleophile. Silica gel column chromatography (DCM = 100%) provided product **5b** (yield: 831 mg, 84%) as an orange solid. m_p_ = 230–231 °C (crystallized from DCM:Hex = 1:40). R_f_ = 0.48 (DCM = 100%). HPLC: t*_R_* = 8.12 min, eluent E_1_. IR (neat) ν (cm^−1^): 2925, 2857, 1640, 1557, 1448, 1304, 1260, 1110, 983, 851, 748, 721. ^1^H-NMR (500 MHz, CDCl_3_) δ (ppm): 12.36 (s, 1H, -NH), 8.08 (d, 2H, ^3^*J* = 7.6 Hz, 2 × H-C(carbazole)), 7.92 (s, 1H, H-C(purine)), 7.83 (d, 2H, ^3^*J* = 9.2 Hz, 2 × H-C(Ar)), 7.65 (d, 2H, ^3^*J* = 7.6 Hz, 2 × H-C(carbazole)), 7.39 (t, 2H, ^3^*J* = 7.6 Hz, 2 × H-C(carbazole)), 7.31 (t, 2H, ^3^*J* = 7.6 Hz, 2 × H-C(carbazole)), 6.57 (d, 2H, ^3^*J* = 9.2 Hz, 2 × H-C(Ar)), 6.55 (s, 1H, H-C(vinyl)), 4.22 (t, 2H, ^3^*J* = 7.3 Hz, (-CH_2_-)), 1.93–1.86 (m, 2H, (-CH_2_-)), 1.36–1.23 (m, 8H, 4 × (-CH_2_-)), 0.88 (t, 3H, ^3^*J* = 7.3 Hz, (-CH_3_)). ^13^C-NMR (126 MHz, CDCl_3_) δ (ppm): 156.1, 152.7, 151.8, 145.2, 143.7, 143.5, 142.5, 138.7, 128.5, 127.0, 125.6, 124.5, 121.9, 120.6, 118.0, 111.6, 93.5, 44.2, 31.7, 30.0, 28.8, 26.6, 22.6, 14.1. HRMS (ESI) *m*/*z*: [M − H]^−^ Calcd for C_32_H_29_ClN_7_O_2_ 578.2077; Found 578.2088 (1.9 ppm).

(*E*)-*N*-[1-(9*H*-Carbazol-9-yl)-2-(2-chloro-9-heptyl-9*H*-purin-6-yl)vinyl]-4-methoxyaniline (**5c**)

Method **A**, 300 mg of **4c** [32], with carbazole as the nucleophile. Silica gel column chromatography (DCM/MeCN, gradient 0%→4%) provided product **=** (yield: 275 mg, 69%) as an orange amorphous solid. R_f_ = (DCM/MeCN = 20:1). HPLC: t*_R_* = 8.60 min, eluent E_1_. IR (neat) ν (cm^−1^): 2927, 2855, 1631, 1575, 1556, 1507, 1446, 1293, 1224, 1030, 984, 748, 722. ^1^H-NMR (500 MHz, CDCl_3_) δ (ppm): 11.93 (s, 1H, -NH), 8.03 (d, 2H, ^3^*J* = 7.8 Hz, 2 × H-C(carbazole)), 7.84 (s, 1H, H-C(purine)), 7.68 (d, 2H, ^3^*J* = 7.8 Hz, 2 × H-C(carbazole)), 7.38 (t, 2H, ^3^*J* = 7.8 Hz, 2 × H-C(carbazole)), 7.26 (t, 2H, ^3^*J* = 7.8 Hz, 2 × H-C(carbazole)), 6.67 (d, 2H, ^3^*J* = 8.9 Hz, 2 × H-C(Ar)), 6.49 (d, 2H, ^3^*J* = 8.9 Hz, 2 × H-C(Ar)), 6.29 (s, 1H, H-C(vinyl)), 4.18 (t, 2H, ^3^*J* = 7.3 Hz, (-CH_2_-)), 3.56 (s, 3H, -OMe), 1.88 (quintet, 2H, ^3^*J* = 7.3 Hz, (-CH_2_-)), 1.38–1.24 (m, 8H, 4 × (-CH_2_-)), 0.89 (t, 3H, ^3^*J* = 7.3 Hz, (-CH_3_)). ^13^C-NMR (126 MHz, CDCl_3_) δ (ppm): 157.2, 156.2, 152.8, 150.9, 146.6, 142.3, 139.2, 132.2, 127.7, 126.6, 124.2, 121.6, 121.2, 120.2, 114.5, 111.9, 89.1, 55.3, 44.0, 31.7, 30.1, 28.8, 26.6, 22.6, 14.1. HRMS (ESI) *m*/*z*: [M − H]^−^ Calcd for C_33_H_32_ClN_6_O 563.2332; Found 563.2345 (2.31 ppm).

(*E*)-*N*-{[1-(9*H*-Carbazol-9-yl)-2-(2-chloro-9-heptyl-9*H*-purin-6-yl)vinyl]amino}benzonitrile (**5d**)

Method **A**, 500 mg of **4d**, with carbazole as the nucleophile. Silica gel column chromatography (DCM/MeCN, gradient 0%→4%) provided product **5d** (yield: 588 mg, 88%) as a yellow amorphous solid. R_f_ = 0.82 (DCM/MeCN = 20:1). HPLC: t*_R_* = 8.12 min, eluent E_2_. IR (neat) ν (cm^−1^): 2926, 2855, 2222, 1637, 1580, 1556, 1509, 1445, 1309, 1256, 1223, 982, 831, 748, 722. ^1^H-NMR (500 MHz, CDCl_3_) δ (ppm): 12.27 (s, 1H, (-NH)), 8.09 (d, 2H, ^3^*J* = 7.6 Hz, 2 × H-C(carbazole)), 7.94 (s, 1H, H-C(purine)), 7.64 (d, 2H, ^3^*J* = 8.7 Hz, 2 × H-C(Ar)), 7.40 (t, 2H, ^3^*J* = 7.6 Hz, 2 × H-C(carbazole)), 7.32 (t, 2H, ^3^*J* = 7.6 Hz, 2 × H-C(carbazole)), 7.24 (d, 2H, ^3^*J* = 7.6 Hz, 2 × H-C(carbazole)), 6.60 (d, 2H, ^3^*J* = 8.7 Hz, 2 × H-C(Ar)), 6.50 (s, 1H, H-C(vinyl)), 4.24 (t, 2H, ^3^*J* = 7.3 Hz, (-CH_2_-)), 1.96–1.86 (m, 2H, (-CH_2_-)), 1.38–1.22 (m, 8H, 4 × (-CH_2_-)), 0.88 (t, 3H, ^3^*J* = 7.3 Hz, (-CH_3_)). ^13^C-NMR (126 MHz, CDCl_3_) δ (ppm): 156.4, 152.8, 151.8, 144.2, 143.40, 143.36, 138.8, 133.7, 128.5, 127.0, 124.6, 121.9, 120.7, 119.0, 118.8, 111.8, 105.8, 92.8, 44.2, 31.8, 30.1, 28.8, 26.7, 22.7, 14.2. HRMS (ESI) *m*/*z*: [M − H]^−^ Calcd for C_33_H_29_ClN_7_ 558.2178; Found 558.2189 (1.97 ppm).

(*E*)-*N*^1^-[1-(9*H*-Carbazol-9-yl)-2-(2-chloro-9-heptyl-9*H*-purin-6-yl)vinyl]-*N*^4^,*N*^4^-dimethylbenzene-1,4-diamine (**5e**)

Method **A**, 405 mg of **4e**, with carbazole as the nucleophile. Silica gel column chromatography (DCM/MeCN, gradient 0%→5%) provided product **5e** (yield: 409 mg, 68%) as a red amorphous solid. R_f_ = 0.73 (DCM/MeCN = 20:1). HPLC: t*_R_* = 5.34 min, eluent E_1_. IR (neat) ν (cm^−1^): 2925, 2854, 1631, 1573, 1553, 1516, 1445, 1292, 1224, 984, 807, 748, 722. ^1^H-NMR (500 MHz, CDCl_3_) δ (ppm): 11.93 (s, 1H, (-NH)), 8.03 (d, 2H, ^3^*J* = 7.9 Hz, 2 × H-C(carbazole)), 7.83 (s, 1H, H-C(purine)), 7.66 (d, 2H, ^3^*J* = 7.9 Hz, 2 × H-C(carbazole)), 7.38 (t, 2H, ^3^*J* = 7.9 Hz, 2×H-C(carbazole)), 7.25 (t, 2H, ^3^*J* = 7.8 Hz, 2 × H-C(carbazole)), 6.65 (d, 2H, ^3^*J* = 8.8 Hz, 2 × H-C(Ar)), 6.35 (d, 2H, ^3^*J* = 8.8 Hz, 2 × H-C(Ar)), 6.20 (s, 1H, H-C(vinyl)), 4.20 (t, 2H, ^3^*J* = 7.3 Hz, (-CH_2_-)), 2.74 (s, 6H, (-NMe_2_)), 1.89 (quintet, 2H, ^3^*J* = 7.3 Hz, (-CH_2_-)), 1.35–1.26 (m, 8H, 4 × (-CH_2_-)), 0.88 (t, 3H, ^3^*J* = 7.3 Hz, (-CH_3_)). ^13^C-NMR (126 MHz, CDCl_3_) δ (ppm): 157.4, 153.0, 150.8, 147.6, 146.9, 142.1, 139.3, 128.7, 127.6, 126.6, 124.3, 121.6, 121.0, 120.2, 113.2, 112.0, 88.4, 44.0, 40.7, 31.8, 30.2, 28.9, 26.7, 22.7, 14.2. HRMS (ESI) *m*/*z*: [M − H]^−^ Calcd for C_34_H_35_ClN_7_ 576.2648; Found 576.2664 (2.78 ppm).

(*E*)-*N*-(1-(9*H*-Carbazol-9-yl)-2-(9-heptyl-2-(1-phenyl-1*H*-1,2,3-triazol-4-yl)-9*H*-purin-6-yl)vinyl)aniline (**5h**)

Method **A**, 303 mg of **4h**, with carbazole as a nucleophile. Silica gel column chromatography (DCM/EtOH, gradient 0%→2%) provided product **5h** (yield: 130 mg, 33%) as a yellow solid. m_p_ = 185–186 °C (crystallized from DCM:Hex = 1:40). R_f_ = 0.7 (DCM/EtOH = 20:1). IR (neat) ν (cm^−1^): 3056, 2919, 2853, 1646, 1587, 1560, 1499, 1439, 1316, 1255, 1028, 746. ^1^H-NMR (500 MHz, CDCl_3_) δ (ppm): 13.19 (s, 1H, -NH), 8.70 (s, 1H, H-C(triazole)), 8.08 (d, 2H, ^3^*J* = 7.7 Hz, 2 × H-C(carbazole)), 7.94 (s, 1H, H-C(purine)), 7.87 (d, 2H, ^3^*J* = 7.7 Hz, 2 × H-C(carbazole)), 7.77 (d, 2H, ^3^*J* = 8.2 Hz, H-C(Ph)), 7.58 (t, 2H, ^3^*J* = 7.7 Hz, 2 × H-C(carbazole)), 7.49 (t, 1H, ^3^*J* = 8.2 Hz, H-C(Ph)), 7.39 (t, 2H, ^3^*J* = 7.7 Hz, 2 × H-C(carbazole)), 7.28 (t, 2H, ^3^*J* = 8.2 Hz, H-C(Ph)), 7.00 (t, 2H, ^3^*J* = 7.8 Hz, H-C(Ph)), 6.89 (d, 2H, ^3^*J* = 7.8 Hz, H-C(Ph)), 6.80 (t, 1H, ^3^*J* = 7.8 Hz, H-C(Ph)), 6.46 (s, 1H, H-C(vinyl)), 4.31 (t, 2H, ^3^*J* = 6.9 Hz, (-CH_2_-)), 2.04–1.92 (m, 2H, (-CH_2_-)), 1.44–1.34 (m, 4H, 2 × (-CH_2_-)), 1.34–1.24 (m, 4H, 2 × (-CH_2_-)), 0.88 (t, 3H, ^3^*J* = 6.9 Hz, (-CH_3_)). ^13^C-NMR (126 MHz, CDCl_3_) δ (ppm): 155.8, 151.4, 150.6, 148.9, 145.4, 142.7, 139.9, 139.4, 137.2, 130.0, 129.3, 129.1, 128.4, 126.6, 124.3, 123.1, 122.2, 121.1, 120.8, 120.3, 119.5, 112.1, 90.4, 43.9, 31.8, 30.2, 28.9, 26.8, 22.7, 14.2. HRMS (ESI) *m*/*z*: [M + H]^+^ Calcd for C_40_H_38_N_9_ 644.3245; Found 644.3234 (1.70 ppm).

9-[3-(2-Chloro-9-heptyl-9*H*-purin-6-yl)-1*H*-indol-2-yl]-9*H*-carbazole (**6a**)

Method **B**, 100 mg of **5a**. Silica gel column chromatography (DCM/EtOH, gradient 0%→0.5%) provided product **6a** (yield: 84 mg, 84%) as an orange amorphous solid. R_f_ = 0.79 (DCM/EtOH = 20:1). HPLC: t*_R_* = 5.96 min, eluent E_1_. IR (neat) ν (cm^−1^): 3365, 2926, 2855, 1567, 1444, 1313, 1227, 1153, 845, 745, 721. ^1^H-NMR (500 MHz, CDCl_3_) δ (ppm): 9.38 (s, 1H, (-NH)), 8.52 (d, 1H, ^3^*J* = 8.0 Hz, H-C(indole)), 7.87 (d, 2H, ^3^*J* = 7.7 Hz, 2 × H-C(carbazole)), 7.34 (t, 1H, ^3^*J* = 8.0 Hz, H-C(indole)), 7.22 (t, 1H, ^3^*J* = 8.0 Hz, H-C(indole)), 7.09–7.00 (m, 5H, 1 × H-C(indole), 4 × H-C(carbazole)), 6.94 (d, 2H, ^3^*J* = 7.7 Hz, 2 × H-C(carbazole)), 6.86 (s, 1H, H-C(purine)), 3.72 (t, 2H, ^3^*J* = 7.2 Hz, (-CH_2_-)), 1.50 (quintet, 2H, ^3^*J* = 7.2 Hz, (-CH_2_-)), 1.28–1.20 (m, 2H, (-CH_2_-)), 1.20–1.12 (m, 4H, 2×(-CH_2_-)), 1.01–0.93 (m, 2H, (-CH_2_-)), 0.87 (t, 3H, ^3^*J* = 7.2 Hz, (-CH_3_)). ^13^C-NMR (126 MHz, CDCl_3_) δ (ppm): 153.8, 153.5, 152.9, 143.3, 140.7, 134.1, 133.2, 129.4, 126.4, 126.0, 123.8, 123.7, 122.10, 122.07, 120.4, 119.9, 111.4, 110.2, 106.2, 43.5, 31.6, 29.4, 28.6, 26.3, 22.6, 14.1. HRMS (ESI) *m*/*z*: [M − H]^−^ Calcd for C_32_H_28_ClN_5_ 531.2069; Found 531.2078 (1.69 ppm).

Method **C**, 200 mg of **4a**, with carbazole as the nucleophile. Silica gel column chromatography (DCM/EtOH, gradient 0%→0.5%) provided product **6a** (yield: 169 mg, 63%) as an orange amorphous solid.

9-[3-(2-Chloro-9-heptyl-9*H*-purin-6-yl)-5-nitro-1*H*-indol-2-yl]-9*H*-carbazole (**6b**)

Method **B**, 200 mg of **5b**. Silica gel column chromatography (DCM/MeCN, gradient 0%→3%) provided product **6b** (yield: 120 mg, 60%) as a red solid. m_p_ = 242–243 °C (crystallized from acetone: MeCN = 1:40). R_f_ = 0.59 (DCM/EtOH = 20:1). HPLC: t*_R_* = 5.47 min, eluent E_1_. IR (neat) ν (cm^−1^): 2925, 2855, 1568, 1446, 1314, 1216, 1152, 838, 741, 722. ^1^H-NMR (500 MHz, CDCl_3_) δ (ppm): 9.56 (s, 1H, (-NH)), 9.38 (d, 1H, ^4^*J* = 2.3 Hz, H-C(indole)), 8.16 (dd, 1H, ^3^*J* = 8.9 Hz, ^4^*J* = 2.3 Hz, H-C(indole)), 7.97–7.91 (m, 2H, 2 × H-C(carbazole)), 7.46 (s, 1H, H-C(purine)), 7.22 (d, 1H, ^3^*J* = 8.9 Hz, H-C(indole)), 7.15–7.07 (m, 4H, 4 × H-C(carbazole)), 7.06–7.00 (m, 2H, 2 × H-C(carbazole)), 4.03 (t, 2H, ^3^*J* = 7.0 Hz, (-CH_2_-)), 1.73 (quintet, 2H, ^3^*J* = 7.0 Hz, (-CH_2_-)), 1.28–1.20 (m, 6H, 3 × (-CH_2_-)), 1.13 (quintet, 2H, ^3^*J* = 7.0 Hz, (-CH_2_-)), 0.87 (t, 3H, ^3^*J* = 7.0 Hz, (-CH_3_)). ^13^C-NMR (126 MHz, CDCl_3_) δ (ppm): 153.9, 153.6, 152.3, 144.3, 143.5, 140.1, 136.9, 135.6, 129.7, 126.2, 125.9, 124.3, 121.1, 120.4, 119.7, 119.4, 111.3, 109.8, 107.4, 44.0, 31.7, 29.7, 28.7, 26.5, 22.7, 14.2. HRMS (ESI) *m*/*z*: [M − H]^−^ Calcd for C_32_H_27_ClN_7_O_2_ 576.1920; Found 576.1930 (1.74 ppm).

Method **C**, 220 mg of **4b**, with carbazole as the nucleophile. Silica gel column chromatography (DCM/MeCN, gradient 0%→3%) provided product **6b** (yield: 139 mg, 48%) as a red amorphous solid.

9-[3-(2-Chloro-9-heptyl-9*H*-purin-6-yl)-5-methoxy-1*H*-indol-2-yl]-9*H*-carbazole (**6c**)

Method **B**, 200 mg of **5c**. Silica gel column chromatography (DCM/MeCN, gradient 0%→5%) provided product **6c** (yield: 149 mg, 75%) as a red amorphous solid. R_f_ = 0.88 (DCM/MeCN = 20:1). HPLC: t*_R_* = 5.74 min, eluent E_1_. IR (neat) ν (cm^−1^): 3362, 2927, 2855, 1568, 1450, 1313, 1215, 1143, 1029, 858, 800, 747, 721. ^1^H-NMR (500 MHz, CDCl_3_) δ (ppm): 9.44 (s, 1H, (-NH)), 8.09 (d, 1H, ^4^*J* = 2.3 Hz, H-C(indole)), 7.83 (d, 2H, ^3^*J* = 7.4 Hz, 2 × H-C(carbazole)), 7.02 (t, 2H, ^3^*J* = 7.4 Hz, 2 × H-C(carbazole)), 6.99 (t, 2H, ^3^*J* = 7.4 Hz, 2 × H-C(carbazole)), 6.91 (d, 2H, ^3^*J* = 7.4 Hz, 2 × H-C(carbazole)), 6.90 (d, 1H, ^3^*J* = 8.8 Hz, H-C(indole)), 6.85 (dd, 1H, ^3^*J* = 8.8 Hz, ^4^*J* = 2.3 Hz, H-C(indole)), 6.80 (s, 1H, H-C(purine)), 3.94 (s, 3H, (-OMe)), 3.70 (t, 2H, ^3^*J* = 7.2 Hz, (-CH_2_-)), 1.48 (quintet, 2H, ^3^*J* = 7.2 Hz, (-CH_2_-)), 1.27–1.20 (m, 2H, (-CH_2_-)), 1.20–1.13 (m, 4H, 2 × (-CH_2_-)), 0.94 (quintet, 2H, ^3^*J* = 7.2 Hz, (-CH_2_-)), 0.87 (t, 3H, ^3^*J* = 7.0 Hz, (-CH_3_)). ^13^C-NMR (126 MHz, CDCl_3_) δ (ppm): 155.7, 153.9, 153.5, 152.8, 143.1, 140.6, 133.6, 129.1, 129.0, 127.0, 125.9, 123.6, 120.3, 119.8, 113.8, 112.3, 110.1, 105.9, 103.9, 55.8, 43.4, 31.5, 29.3, 28.5, 26.2, 22.5, 14.1. HRMS (ESI) *m*/*z*: [M − H]^−^ Calcd for C_33_H_30_ClN_6_O 561.2175; Found 561.2184 (1.6 ppm).

Method **C**, 212 mg of **4c**, with carbazole as the nucleophile. Silica gel column chromatography (DCM/MeCN, gradient 0%→5%) provided product **6c** (yield: 133 mg, 48%) as a red amorphous solid.

2-(9*H*-Carbazol-9-yl)-3-(2-chloro-9-heptyl-9*H*-purin-6-yl)-1*H*-indole-5-carbonitrile (**6d**)

Method **B**, 100 mg of **5d**. After workup, the resulting solid was suspended in EtOAc (4 mL) and filtered, providing product **6d** (yield: 57 mg, 57%) as a beige amorphous solid. IR (neat) ν (cm^−1^): 2927, 2856, 2217, 1571, 1479, 1452, 1314, 1214, 1160, 933, 795, 802, 747, 723. ^1^H-NMR (500 MHz, DMSO-*d_6_*) δ (ppm): 13.37 (s, 1H, (-NH)), 8.91 (s, 1H, H-C(indole)), 8.20 (d, 2H, ^3^*J* = 7.7 Hz, 2 × H-C(carbazole)), 8.14 (s, 1H, H-C(purine)), 7.75–7.70 (m, 2H, 2 × H-C(indole)), 7.28 (t, 2H, ^3^*J* = 7.7 Hz, 2 × H-C(carbazole)), 7.24 (t, 2H, ^3^*J* = 7.7 Hz, 2 × H-C(carbazole)), 7.19 (d, 2H, ^3^*J* = 7.7 Hz, 2 × H-C(carbazole)), 4.04 (t, 2H, ^3^*J* = 6.9 Hz, (-CH_2_-)), 1.66 (quintet, 2H, ^3^*J* = 6.9 Hz, (-CH_2_-)), 1.25–1.12 (m, 6H, 3 × (-CH_2_-)), 0.99 (quintet, 2H, ^3^*J* = 6.9 Hz, (-CH_2_-)), 0.82 (t, 3H, ^3^*J* = 6.9 Hz, (-CH_3_)). ^13^C-NMR (126 MHz, DMSO-*d_6_*) δ (ppm): 153.3, 152.13, 152.10, 146.1, 139.9, 136.5, 135.8, 129.2, 127.2, 126.1, 125.9, 125.8, 123.6, 120.7, 120.5, 120.4, 113.5, 110.2, 104.7, 103.3, 43.1, 31.1, 28.7, 28.0, 25.6, 22.0, 13.9. HRMS (ESI) *m*/*z*: [M − H]^−^ Calcd for C_33_H_27_ClN_7_ 556.2022; Found 556.2029 (1.26 ppm).

Method **C**, 210 mg of **4d**, with carbazole as the nucleophile. Silica gel column chromatography (DCM/EtOH, gradient 0%→1%) provided product **6d** (yield: 194 mg, 70%) as a red amorphous solid.

9-[3-(9-Heptyl-9*H*-purin-6-yl)-1*H*-indol-2-yl]-9*H*-carbazole (**6f**)

Method **C**, 100 mg of **4f**, with carbazole as the nucleophile. Silica gel column chromatography (DCM/MeCN, gradient 0%→8%) provided product **6f** (yield: 48 mg, 35%) as a brown amorphous solid. R_f_ = 0.45 (DCM/MeCN = 20:1). HPLC: t*_R_* = 3.49 min, eluent E_1_. IR (neat) ν (cm^−1^): 3365, 2927, 2855, 1579, 1448, 1322, 1228, 841, 745, 722. ^1^H-NMR (500 MHz, CDCl_3_) δ (ppm): 9.26 (s, 1H, (-NH)), 8.78 (s, 1H, H-C(purine)), 8.40 (d, 1H, ^3^*J* = 7.9 Hz, H-C(indole)), 7.98–7.93 (m, 2H, 2 × H-C(carbazole)), 7.34–7.29 (m, 1H, H-C(indole)), 7.28–7.22 (m, 2H, 2 × H-C(indole)), 7.17 (s, 1H, H-C(purine)), 7.16–7.10 (m, 6H, 6 × H-C(carbazole)), 3.92 (t, 2H, ^3^*J* = 7.1 Hz, (-CH_2_-)), 1.63 (quintet, 2H, ^3^*J* = 7.1 Hz, (-CH_2_-)), 1.27–1.13 (m, 6H, 3 × (-CH_2_-)), 1.04 (quintet, 2H, ^3^*J* = 7.1 Hz, (-CH_2_-)), 0.86 (t, 3H, ^3^*J* = 7.1 Hz, (-CH_3_)). ^13^C-NMR (126 MHz, CDCl_3_) δ (ppm): 152.5, 152.2, 151.4, 143.2, 140.7, 134.2, 132.4, 131.1, 126.8, 126.1, 123.8, 123.6, 121.9, 121.8, 120.5, 120.0, 111.3, 110.3, 107.1, 43.6, 31.7, 29.7, 28.7, 26.4, 22.6, 14.2. HRMS (ESI) *m*/*z*: [M − H]^−^ Calcd for C_32_H_29_N_6_ 497.2459; Found 497.2466 (1.41 ppm).

9-[3-(9-Heptyl-9*H*-purin-6-yl)-5-nitro-1*H*-indol-2-yl]-9*H*-carbazole (**6g**)

Method **C**, 100 mg of **4g**, with carbazole as the nucleophile. Silica gel column chromatography (DCM/MeCN, gradient 0%→8%) provided product **6g** (yield: 74 mg, 55%) as a brown amorphous solid. R_f_ = 0.60 (DCM/MeCN = 20:1). HPLC: t*_R_* = 4.19 min, eluent E_1_. IR (neat) ν (cm^−1^): 2924, 2857, 1580, 1449, 1322, 1225, 1003, 838, 747, 723. ^1^H-NMR (500 MHz, DMSO-*d_6_*) δ (ppm): 13.34 (s, 1H, (-NH)), 9.34 (d, 1H, ^4^*J* = 2.3 Hz, H-C(indole)), 8.72 (s, 1H, H-C(purine)), 8.23 (dd, 1H, ^3^*J* = 9.0 Hz, ^4^*J* = 2.3 Hz, H-C(indole)), 8.19 (d, 2H, ^3^*J* = 7.5 Hz, 2 × H-C(carbazole)), 8.12 (s, 1H, H-C(purine)), 7.72 (d, 1H, ^3^*J* = 9.0 Hz, H-C(indole)), 7.29–7.15 (m, 6H, 6 × H-C(carbazole)), 4.10 (t, 2H, ^3^*J* = 6.9 Hz, (-CH_2_-)), 1.69 (quintet, 2H, ^3^*J* = 6.9 Hz, (-CH_2_-)), 1.25–1.12 (m, 6H, 3 × (-CH_2_-)), 0.99 (quintet, 2H, ^3^*J* = 6.9 Hz, (-CH_2_-)), 0.82 (t, 3H, ^3^*J* = 6.9 Hz, (-CH_3_)). ^13^C-NMR (126 MHz, DMSO-*d_6_*) δ (ppm): 151.6, 151.5, 150.3, 145.4, 141.9, 139.8, 137.9, 135.4, 130.3, 126.1, 125.7, 123.5, 120.6, 120.4, 118.6, 118.1, 112.6, 110.2, 106.9, 42.9, 31.1, 28.9, 28.0, 25.7, 22.0, 13.9. HRMS (ESI) *m*/*z*: [M − H]^−^ Calcd for C_32_H_28_N_7_O_2_ 542.2310; Found 542.2318 (1.48 ppm).

(*E*)-2-(2-Chloro-9-heptyl-9*H*-purin-6-yl)-*N*,*N*,*N’*-triphenylethene-1,1-diamine (**7a**)

Method **A**, 200 mg of **4a** [32], with diphenylamine as the nucleophile. Silica gel column chromatography (DCM/EtOH, gradient 0%→2%) provided product **7a** (yield: 215 mg, 70%) as a yellow amorphous solid. R_f_ = 0.71 (DCM/EtOH = 20:1). HPLC: t*_R_* = 6.72 min, eluent E_2_. IR (neat) ν (cm^−1^): 2926, 2855, 1619, 1545, 1489, 1366, 1241, 976, 748, 691. ^1^H-NMR (500 MHz, CDCl_3_) δ (ppm): 11.68 (s, 1H, (-NH)), 7.73 (s, 1H, H-C(purine)), 7.18 (t, 4H, ^3^*J* = 7.8 Hz, 4 × H-C(Ph)), 7.13–7.06 (m, 8H, 8 × H-C(Ph)), 7.00 (t, 2H, ^3^*J* = 7.8 Hz, 2 × H-C(Ph)), 6.90 (t, 1H, ^3^*J* = 6.6 Hz, H-C(Ph)), 5.73 (s, 1H, H-C(vinyl)), 4.14 (t, 2H, ^3^*J* = 7.0 Hz, (-CH_2_-)), 1.85 (quintet, 2H, ^3^*J* = 7.0 Hz, (-CH_2_-)), 1.37–1.20 (m, 8H, 4 × (-CH_2_-)), 0.87 (t, 3H, ^3^*J* = 7.0 Hz, (-CH_3_)). ^13^C-NMR (126 MHz, CDCl_3_) δ (ppm): 157.8, 156.6, 152.8, 150.2, 145.1, 141.2, 139.0, 129.2, 128.7, 126.7, 124.9, 124.5, 123.9, 122.2, 86.6, 43.9, 31.8, 30.2, 28.9, 26.7, 22.7, 14.2. HRMS (ESI) *m*/*z*: [M − H]^−^ Calcd for C_32_H_32_ClN_6_ 535.2382; Found 535.2396 (2.62 ppm).

(*E*)-2-(2-Chloro-9-heptyl-9*H*-purin-6-yl)-*N*-(4-nitrophenyl)-*N’*,*N’*-diphenylethene-1,1-diamine (**7b**)

Method **A**, 200 mg of **4b** [32], with diphenylamine as the nucleophile. Silica gel column chromatography (DCM/MeCN, gradient 0%→3%) provided product **7b** (yield: 120 mg, 45%) as a brown amorphous solid. R_f_ = 0.63 (DCM/MeCN = 20:1). HPLC: t*_R_* = 8.63 min, eluent E_2_. IR (neat) ν (cm^−1^): 2923, 2855, 1629, 1550, 1454, 1303, 1245, 1107, 977, 844, 754, 693. ^1^H-NMR (500 MHz, CDCl_3_) δ (ppm): 12.10 (s, 1H, (-NH)), 8.00 (d, 2H, ^3^*J* = 9.0 Hz, 2 × H-C(Ar-NO_2_)), 7.80 (s, 1H, H-C(purine)), 7.28–7.23 (m, 6H, 2 × H-C(Ar-NO_2_), 4 × H-C(Ph)), 7.17 (d, 4H, ^3^*J* = 8.4 Hz, 4 × H-C(Ph)), 7.08 (t, 2H, ^3^*J* = 8.4 Hz, 2 × H-C(Ph)), 5.87 (s, 1H, H-C(vinyl)), 4.16 (t, 2H, ^3^*J* = 7.3 Hz, (-CH_2_-)), 1.86 (quintet, 2H, ^3^*J* = 7.3 Hz, (-CH_2_-)), 1.33–1.19 (m, 8H, 4 × (-CH_2_-)), 0.87 (t, 3H, ^3^*J* = 7.3 Hz, (-CH_3_)). ^13^C-NMR (126 MHz, CDCl_3_) δ (ppm): 157.0, 154.0, 152.6, 151.0, 145.2, 144.6, 142.4, 142.2, 129.6, 127.4, 125.2, 125.1, 124.8, 119.1, 89.9, 44.1, 31.7, 30.1, 28.8, 26.7, 22.7, 14.2. HRMS (ESI) *m*/*z*: [M − H]^−^ Calcd for C_32_H_31_ClN_7_O_2_ 580.2233; Found 580.2245 (2.07 ppm).

3-(2-Chloro-9-heptyl-9*H*-purin-6-yl)-*N*,1-diphenyl-1*H*-indol-2-amine (**8a**)

Method **B**, 100 mg of **7a**. Silica gel column chromatography (DCM/EtOH, gradient 0%→1%) provided product **8a** (yield: 54 mg, 54%) as a yellow amorphous solid. R_f_ = 0.92 (DCM/EtOH = 20:1). HPLC: t*_R_* = 9.35 min, eluent E_1_. IR (neat) ν (cm^−1^): 2926, 2859, 1633, 1559, 1466, 1435, 1310, 1225, 1074, 1029, 883, 744, 691. ^1^H-NMR (500 MHz, CDCl_3_) δ (ppm): 11.30 (s, 1H, (-NH)), 8.90 (d, 1H, ^3^*J* = 8.0 Hz, H-C(indole)), 7.95 (s, 1H, H-C(purine)), 7.45 (d, 2H, ^3^*J* = 7.6 Hz, 2 × H-C(Ph)), 7.35 (t, 1H, ^3^*J* = 8.0 Hz, H-C(indole)), 7.30 (t, 2H, ^3^*J* = 7.6 Hz, 2 × H-C(Ph)), 7.23–7.16 (m, 3H, H-C(Ph), 2 × H-C(indole)), 6.95 (d, 2H, ^3^*J* = 7.7 Hz, 2 × H-C(Ph)), 6.75 (d, 2H, ^3^*J* = 7.7 Hz, 2 × H-C(Ph)), 6.71 (t, 1H, ^3^*J* = 7.7 Hz, 2 × H-C(Ph)), 4.21 (t, 2H, ^3^*J* = 7.2 Hz, (-CH_2_-)), 1.95–1.86 (m, 2H, (-CH_2_-)), 1.40–1.22 (m, 8H, 4 × (-CH_2_-)), 0.88 (t, 3H, ^3^*J* = 7.2 Hz, (-CH_3_)). ^13^C-NMR (126 MHz, CDCl_3_) δ (ppm): 155.4, 154.3, 152.3, 146.2, 142.2, 140.6, 136.8, 136.6, 129.1, 128.6, 127.7, 127.4, 127.1, 126.7, 122.7, 122.4, 122.1, 121.7, 119.3, 109.8, 100.9, 44.1, 31.7, 30.0, 28.8, 26.7, 22.6, 14.1. HRMS (ESI) *m*/*z*: [M + H]^+^ Calcd for C_32_H_32_ClN_6_ 535.2371; Found 535.2366 (0.93 ppm).

Method **C**, 200 mg of **4a**, with diphenylamine as the nucleophile. Silica gel column chromatography (DCM/EtOH, gradient 0%→1%) provided product **8a** (yield: 136 mg, 50%) as a yellow amorphous solid.

3-(2-Chloro-9-heptyl-9*H*-purin-6-yl)-*N*-(4-nitrophenyl)-1-phenyl-1*H*-indol-2-amine (**8b**)

Method **B**, 84 mg of **7b**. Silica gel column chromatography (DCM/EtOH, gradient 0%→2%) provided product **8b** (yield: 12 mg, 14%) as a yellow solid. m_p_ = 135–136 °C (crystallized from DCM:Hex = 1:40). R_f_ = 0.91 (DCM/EtOH = 20:1). HPLC: t*_R_* = 8.52 min, eluent E_1_. IR (neat) ν (cm^−1^): 2924, 2851, 1567, 1488, 1327, 1248, 1111, 1027, 935, 849, 745, 683. ^1^H-NMR (500 MHz, CDCl_3_) δ (ppm): 11.45 (s, 1H, (-NH)), 8.81, (d, 1H, ^3^*J* = 8.0 Hz, H-C(indole)), 8.09 (s, 1H, H-C(purine)), 7.82 (d, 2H, ^3^*J* = 8.9 Hz, 2 × H-C(Ar-NO_2_)), 7.52, (d, 2H, ^3^*J* = 7.9 Hz, 2 × H-C(Ph)), 7.42–7.36 (m, 3H, 2 × H-C(Ph), H-C(indole)), 7.33 (d, 1H, ^3^*J* = 8.0 Hz, H-C(indole)), 7.30–7.26 (m, 2H, 1×H-C(Ph), 1 × H-C(indole)), 6.68 (d, 2H, ^3^*J* = 8.9 Hz, 2 × H-C(Ar-NO_2_)), 4.27 (t, 2H, ^3^*J* = 7.0 Hz, (-CH_2_-)), 1.97–1.89 (m, 2H, (-CH_2_-)), 1.40–1.31 (m, 4H, 2 × (-CH_2_-)), 1.30–1.23 (m, 4H, 2 × (-CH_2_-)), 0.87 (t, 3H, ^3^*J* = 7.0 Hz, (-CH_3_)). ^13^C-NMR (126 MHz, CDCl_3_) δ (ppm): 154.8, 154.7, 152.6, 148.9, 141.5, 141.3, 140.9, 136.3, 136.2, 129.6, 128.3, 127.6, 127.0, 126.1, 125.2, 123.6, 123.3, 122.4, 115.8, 110.3, 103.6, 44.4, 31.7, 30.0, 28.8, 26.8, 22.7, 14.2. HRMS (ESI) *m*/*z*: [M − H]^−^ Calcd for C_32_H_29_ClN_7_O_2_ 578.2077; Found 578.2088 (1.9 ppm).

Method **C**, 220 mg of **4b**, with diphenylamine as the nucleophile. Silica gel column chromatography (DCM/EtOH, gradient 0%→1%) provided product **8b** (yield: 88 mg, 30%) as a yellow amorphous solid.

## 4. Conclusions

We have developed a new metal-free synthetic approach to convert 1-aryl-1*H*-1,2,3-triazoles into indoles by transforming triazole carbon atoms C4 and C5 into indole C2 and C3 atoms. The reaction sequence in the first step involves triazole ring-opening and nitrogen extrusion, followed by Wolff rearrangement. The formed ketenimine intermediate is susceptible to *N*-nucleophile attack and forms ethene-1,1-diamines in a 33–88% yield. The highest yields for this step are observed for the substrates bearing aryl groups with electron-withdrawing substituents such as -NO_2_ (84%) and -CN (88%) groups. Purine, as an electron deficient entity at the C4 position of the triazole ring, facilitates this transformation better than does the -COOMe moiety. In the second step, the oxidative cyclization in the presence of the I_2_/K_2_CO_3_ system provided indoles. Depending on the conformational flexibility of the *N*-substituents at the ethene-1,1-diamines, cyclization occurs in two distinct ways: (1) substrates containing a conformationally non-flexible carbazole ring undergo indolization with the initial triazole’s aryl group in 57–84% yields; (2) substrates containing flexible aryl substituents arising from diarylamine nucleophile undergo indolization with the latter substituent in 14–54% yields. The developed synthetic sequence may also be performed in a one-pot fashion, reaching an indole yield of up to 70%, in some cases. Our findings demonstrate that the purinyl group activates the triazole ring in a similar fashion as that previously reported for electron-withdrawing substituents, such as the -Ts, -CF_2_CF_3_, and -COOMe groups. Moreover, during the indole-forming oxidative cyclization step, the steric factors play a more important role than does the electronic nature of the substituents, as indoles **6b** and **6d** bearing the -NO_2_ and -CN groups were obtained in good yields. Given that the synthesis of 1,2,3-triazoles is well developed, and indoles are important structural motifs in medicinal chemistry, the further expansion of this methodology is expected.

## Figures and Tables

**Table 1:** Synthesis of purine–indole–carbazole conjugates **6** from triazolyl purines **4**.

Entry	Starting Material 4a–h	Substituents		Yield of Comp. 5, % for the Transformation 4→5	Yield of Comp. 6, % for the Transformation 5→6	Yield of Comp. 6, % for the One-Pot Process 4→[5]→6
		X	Y			
1	**4a**	Cl	H	**5a**, 63	**6a**, 84	**6a**, 63
2	**4b**	Cl	NO_2_	**5b**, 84	**6b**, 60	**6b**, 48
3	**4c**	Cl	OMe	**5c**, 69	**6c**, 75	**6c**, 48
4	**4d**	Cl	CN	**5d**, 88	**6d**, 57	**6d**, 70
5	**4e**	Cl	NMe_2_	**5e**, 68 *	-	-
6	**4f**	H	H	-	-	**6f**, 35
7	**4g**	H	NO_2_	-	-	**6g**, 55
8	**4h**	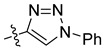	H	**5h**, 33	-	-

* the reaction performed at 25 °C.

## Data Availability

The original contributions presented in this study are included in the article/Supplementary Materials. Further inquiries can be directed to the corresponding authors.

## Supplementary Materials

The following supporting information can be downloaded at: https://www.mdpi.com/article/10.3390/molecules30020337/s1, synthesis procedures, ^1^H- and ^13^C-NMR spectra of starting materials **1d, 1g**, **4d**–**h**; ^1^H- and ^13^C-NMR spectra of compounds **2a**, **2b′**–**d′**, **3a**, **5a**–**e**,**h**, **6a**–**d**, **6f**–**g**, **7a**–**b**, and **8a**–**b**. X-ray crystallography data of compounds **2c′**, **5b**, **5h**, **6b**, and **8b**. Checkcif and Cif files for compounds **2c′**, **5b**, **5h**, **6b**, and **8b**. Starting materials in the Supplementary Materials part were prepared according to the procedures outlined in the literature [32,47,68,69].
