# Supplementary material for: Synthesis of Indole Derivatives via Aryl Triazole Ring-Opening and Subsequent Cyclization
Source: Molecules. 2025 Jan 16;30(2):337. doi: 10.3390/molecules30020337 (PMC11767399; doi:10.3390/molecules30020337)
Supplement: Supplementary file 1 [file molecules-30-00337-s001.zip › molecules-3414132-supplementary.pdf]

Supplementary Materials

Synthesis of Indole Derivatives via Aryl Triazole Ring-Opening and Subsequent Cyclization

Aleksejs Burcevs [1], Armands Sebris [1], Irina Novosjolova [1*], Anatoly Mishnev [2] and Māris Turks [1*]

[1] Institute of Chemistry and Chemical Technology, Faculty of Natural Sciences and Technology, Riga Technical University, P. Valdena Str. 3, LV-1048 Riga, Latvia; aleksejs.burcevs@rtu.lv (A.B.); armands.sebris_1@rtu.lv (A.S.)
[2] Latvian Institute of Organic Synthesis, Aizkraukles Str. 21, LV-1006 Riga, Latvia; mishnevs@osi.lv (A.M.)
* Correspondence: irina.novosjolova@rtu.lv (I.N.); maris.turks@rtu.lv (M.T.)

Table of Contents

1. General information S3
2. Synthesis of 5-(1-(4-nitrophenyl)-1*H*-1,2,3-triazol-4-yl)pyrimidine (**1d**) S4
3. Synthesis of 4-(1-(4-nitrophenyl)-1*H*-1,2,3-triazol-4-yl)butanenitrile (**1g**) S6
4. Synthesis of 2-chloro-6-triazolyl-9-heptylpurines **4d-e** S8
5. Synthesis of 6-triazolyl-9-heptylpurines **4f-g** S12
6. Synthesis of 9-heptyl-2,6-bis(1-phenyl-1*H*-1,2,3-triazol-4-yl)-9*H*-purine (**4h**) S20
7. Spectral data of compounds **2a**, **2b'-d'**, **3a**, **5a-e**,**h**, **6a-d**, **6f-g**, **7a-b**, and **8a-b** S22
8. X-ray crystallography data S43
9. References S84

1. General information

NMR spectra were recorded on Bruker Avance 500 spectrometer. ^{1}H NMR spectra were recorded at 500 MHz with internal references from nondeuterated solvents (δ = 7.26 for $CDCl_3$, δ = 2.50 for DMSO-d_6). ^{13}C NMR spectra were recorded at 125.7 MHz with internal references from nondeuterated solvents (δ = 77.16 for $CDCl_3$, δ = 39.52 for DMSO-d_6). Coupling constants are reported in Hz, chemical shifts of signals are given in ppm, and standard abbreviations were used for multiplicity assignments. Fourier transform infrared (FTIR) spectra were recorded using a Thermo Scientific Nicolet™ iSTM50 (Thermo Fisher, Waltham, MA, USA) spectrometer in the Attenuated Total Reflectance (ATR) mode. Spectra were obtained over a range of wavenumbers from 400 to 4000 cm^{-1}, co-adding 64 scans at 4 cm^{-1} resolution. Before every measurement, a background spectrum was taken and deducted from the sample spectrum. An Orbitrap Exploris 120 (Thermo Scientific, Waltham, MA, USA) mass spectrometer was used for high-resolution mass spectra (ESI). Analytical thin-layer chromatography (TLC) was performed on Merck 60 Å silica gel F_{254} plates. Column chromatography was performed on Merck 40–60 μm 60 Å silica gel or *Licroprep C18* (25–40 μm) silica gel. HPLC analyses were performed using Agilent Technologies 1200 Series system equipped with *XBridge C18* column, 4.6×150 mm, particle size 3.5 μm, with a flow rate of 1 mL/min, using 0.1% TFA/H_2O and MeCN for the mobile phase. The wavelength of detection was 260 nm. Eluent A – 0.1% TFA aqueous solution with 5% v/v MeCN, eluent B – MeCN. Eluent E_1 – gradient 20–95% B 5 min, 95% B 5 min, 95–20% B 2 min. Eluent E_2 – gradient 30–95% B 5 min, 95% B 5 min, 95–30% B 2 min.

2. Synthesis of 5-(1-(4-nitrophenyl)-1*H*-1,2,3-triazol-4-yl)pyrimidine (1d)

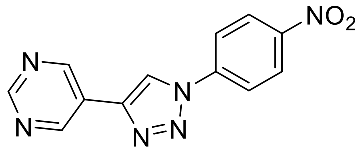

5-((Trimethylsilyl)ethynyl)pyrimidine (1.66 g, 9.42 mmol, 1.0 eq.), 1-azido-4-nitrobenzene [47] (2.32 g, 14.13 mmol, 1.5 eq.), $CuSO_4$•$5H_2O$ (0.45 g, 2.83 mmol, 0.3 eq.) and sodium ascorbate (1.12 g, 5.65 mmol, 0.6 eq.) were suspended in a mixture of *t*-BuOH/acetone/10 w% of AcOH in water (75:19:19 mL) and the reaction mixture was stirred for 1 h at 50 °C temperature and overnight at 25 °C. Then the reaction mixture was poured in H_2O (500 mL), filtered and washed with H_2O (200 mL), DCM (50 mL), and dried in a vacuum, providing product **1d** (yield: 2.07 g, 82%) as a brown amorphous solid. ^{1}H-NMR (500 MHz, DMSO-d$_6$) δ (ppm): 9.75 (s, 1H, H-C(triazole)), 9.35 (s, 2H, 2×H-C(pyrimidine)), 9.26 (s, 1H, H-C(pyrimidine)), 8.54 (d, 2H, ^3J = 8.6 Hz, 2×H-C(Ar)), 8.26 (d, 2H, ^3J = 8.6 Hz, 2×H-C(Ar)). ^{13}C-NMR (126 MHz, DMSO-d$_6$) δ (ppm): 158.2, 153.6, 147.0, 142.2, 140.6, 125.9, 124.4, 121.8, 120.8.

^{1}H-NMR (500 MHz, DMSO-d$_6$) spectrum of compound 1d:

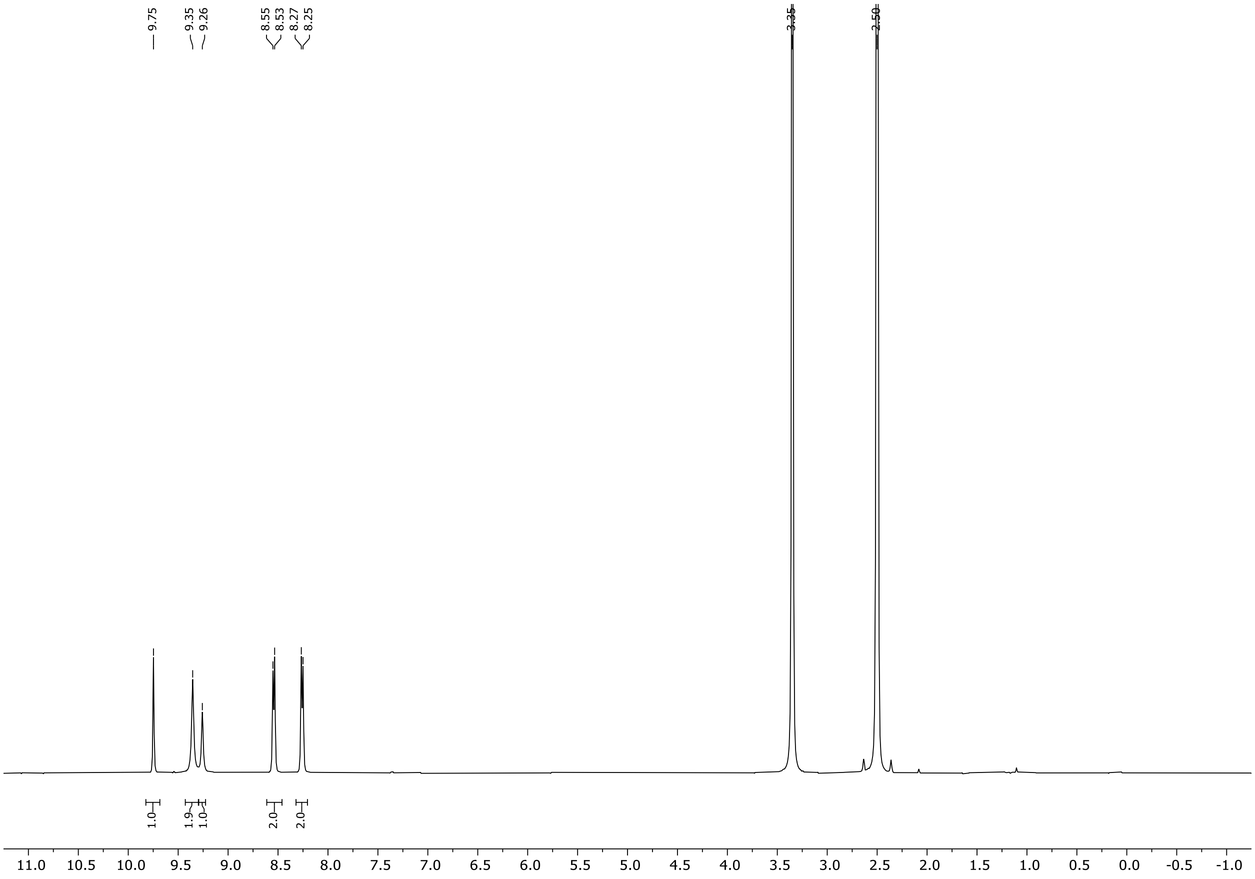

^{13}C-NMR (126 MHz, DMSO-d$_6$) spectrum of compound 1d:

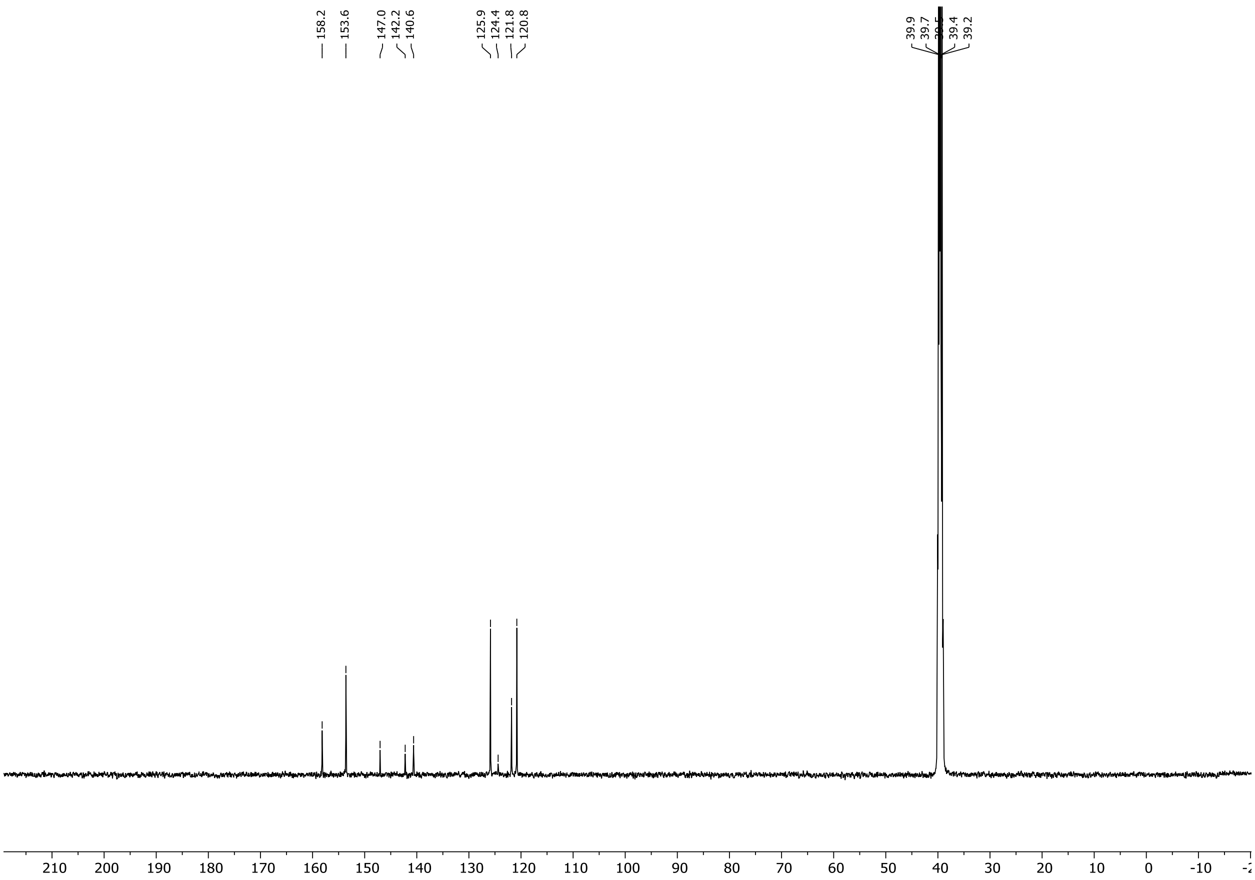

3. Synthesis of 4-(1-(4-nitrophenyl)-1*H*-1,2,3-triazol-4-yl)butanenitrile (1g)

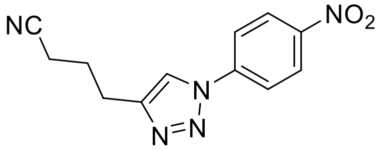

Hex-5-ynenitrile (0.51 mL, ρ = 0.89 g/cm^3, 4.83 mmol, 1.5 eq.), 1-azido-4-nitrobenzene [47] (529 mg, 3.22 mmol, 1.0 eq.), $CuSO_4$•$5H_2O$ (154 mg, 0.97 mmol, 0.3 eq.) and sodium ascorbate (383 mg, 1.93 mmol, 0.6 eq.) were dissolved in DMF (18 mL) and the reaction mixture was stirred at 60 °C temperature overnight. Then, the reaction mixture was poured in H_2O (250 mL), filtered and washed with H_2O (100 mL), and dried in a vacuum, providing product **1g** (yield: 732 mg, 88%) as a grey amorphous solid. ^{1}H-NMR (500 MHz, DMSO-d$_6$) δ (ppm): 8.83 (s, 1H, H-C(triazole)), 8.41 (d, 2H, ^3J = 8.7 Hz, 2×H-C(Ar)), 8.16 (d, 2H, ^3J = 8.7 Hz, 2×H-C(Ar)), 2.83 (t, 2H, ^3J = 7.4 Hz, (-CH_2-)), 2.61 (t, 2H, ^3J = 7.4 Hz, (-CH_2-)), 1.98 (quintet, 2H, ^3J = 7.4 Hz, (-CH_2-)). ^{13}C-NMR (126 MHz, DMSO-*d$_6$*) δ (ppm): 147.2, 146.5, 141.0, 125.6, 121.1, 120.5, 120.3, 24.5, 24.0, 15.8.

^{1}H-NMR (500 MHz, DMSO-d$_6$) spectrum of compound 1g:

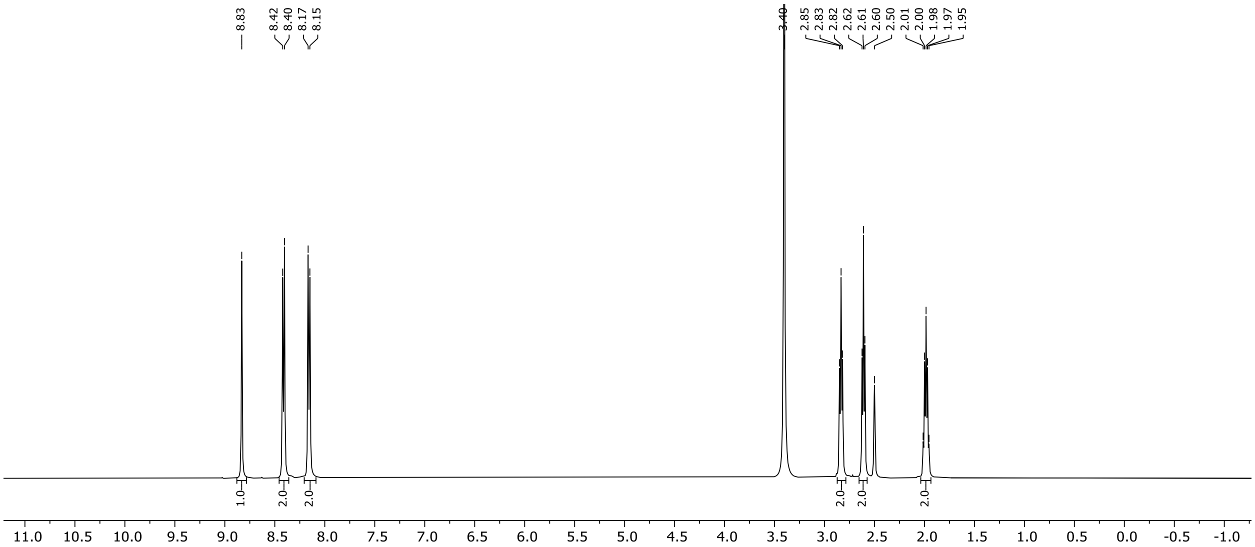

^{13}C-NMR (126 MHz, DMSO-d6) spectrum of compound 1g:

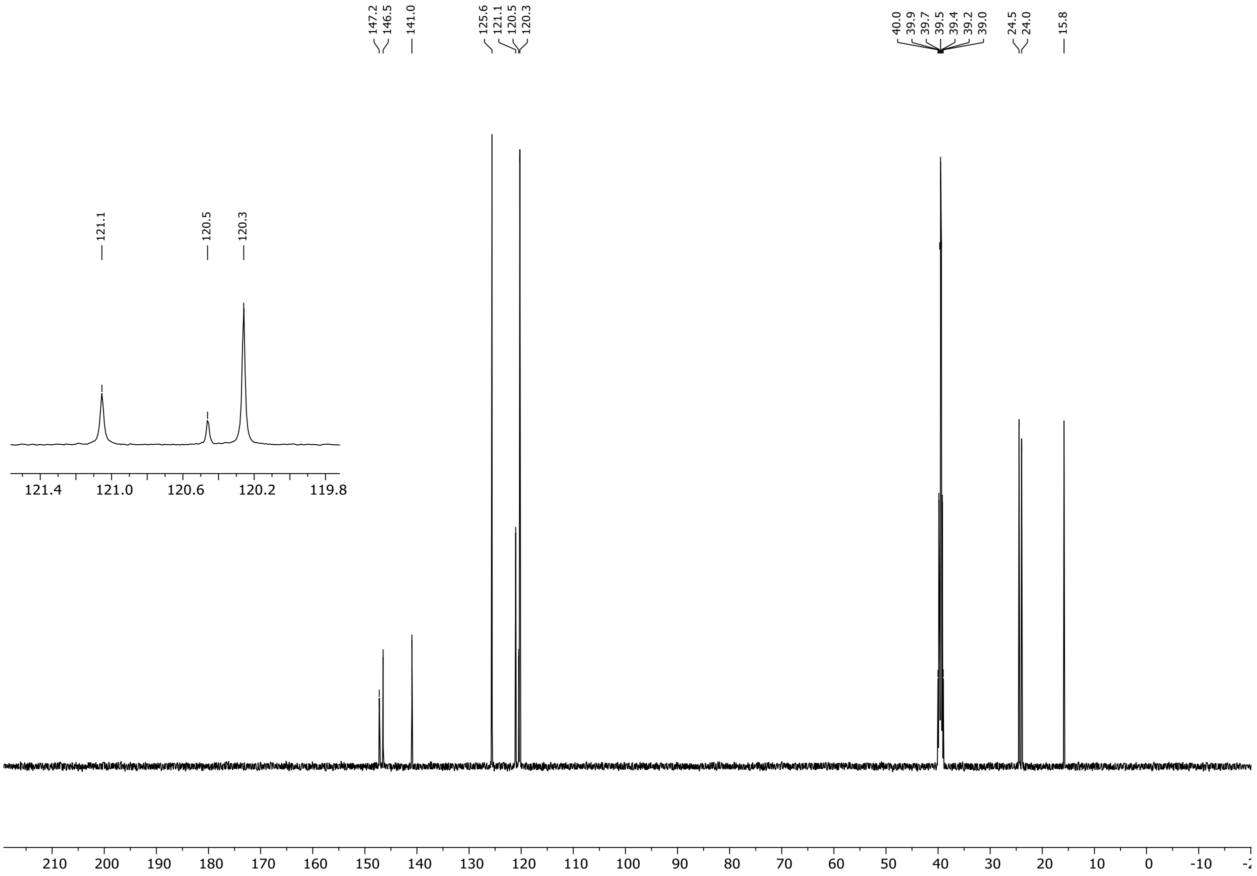

4. Synthesis of 2-chloro-6-triazolyl-9-heptylpurines 4d-e

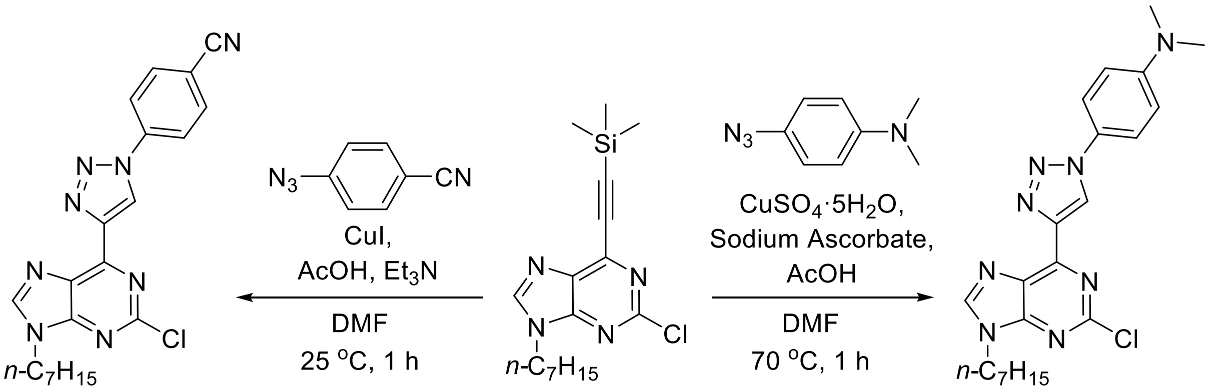

4d **I** **4e**

Scheme S1. Synthesis of 2-chloro-6-triazolyl-9-heptylpurines **4d** and **4e**

4-[4-(2-Chloro-9-heptyl-9*H*-purin-6-yl)-1*H*-1,2,3-triazol-1-yl]benzonitrile (4d)

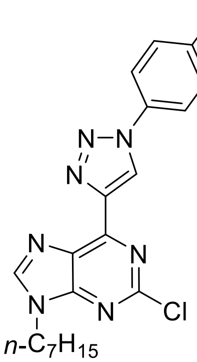

General method D for preparations of triazolyl purines: To a solution of compound **I** [32] (1.47 g, 4.21 mmol, 1.0 eq.), CuI (128 mg, 0.67 mmol, 0.16 eq.), AcOH (0.26 mL, ρ = 1.05 g/cm^3, 4.63 mmol, 1.1 eq.) and Et_3N (0.64 mL, ρ = 0.73 g/cm^3, 4.63 mmol, 1.1 eq.) in DMF (50 mL) 4-azidobenzonitrile [47] (35.1 mL, C = 0.18 M (in DCM), 6.32 mmol, 1.5 eq.) was added and the reaction mixture was stirred isolated from the daylight for 1 h at 25 °C temperature. Then the reaction mixture was poured into water (20 mL) and extracted with DCM (3×10 mL). Combined organic phase was washed with aqueous NaHS (10 mL) and sat. NaCl (10 mL), dried over anhydrous Na_2SO_4, filtered and evaporated. Silica gel column chromatography (DCM/EtOH, gradient 0% → 5%) provided product **4d** (yield: 1.41 g, 79%) as a beige amorphous solid. R_f = 0.48 (DCM/EtOH = 20:1). HPLC: t_R = 6.88 min, eluent E_2. IR (neat) ν (cm^{-1}): 2930, 2854, 2232, 1518, 1333, 1246, 1158, 1092, 1038, 988, 942, 844, 800. ^{1}H-NMR (500 MHz, $CDCl_3$) δ (ppm): 9.22 (s, 1H, H-C(triazole)), 8.16 (s, 1H, H-C(purine)), 8.08 (d, 2H, ^3J = 8.5 Hz, 2×H-C(Ar)), 7.90 (d, 2H, ^3J = 8.5 Hz, 2×H-C(Ar)), 4.30 (t, 2H, ^3J = 7.3 Hz, (-CH_2-)), 2.01–1.90 (m, 2H, (-CH_2-)), 1.42–1.32 (m, 4H, 2×(-CH_2-)), 1.32–1.25 (m, 4H, 2×(-CH_2-)), 0.88 (t, 3H, ^3J = 7.3 Hz, (-CH_3)). ^{13}C-NMR (126 MHz, $CDCl_3$) δ (ppm): 154.7, 154.3, 148.3, 146.1, 144.7, 139.6, 134.2, 129.2, 124.6, 121.1, 117.7, 113.1, 44.4, 31.7, 30.0, 28.8, 26.7, 22.6, 14.2. HRMS (ESI) m/z: [M+H]$^+$ Calcd for $C_{21}H_{22}ClN_8$ 421.1650; Found 421.1651 (0.24 ppm).

^{1}H-NMR (500 MHz, $CDCl_3$) spectrum of compound 4d:

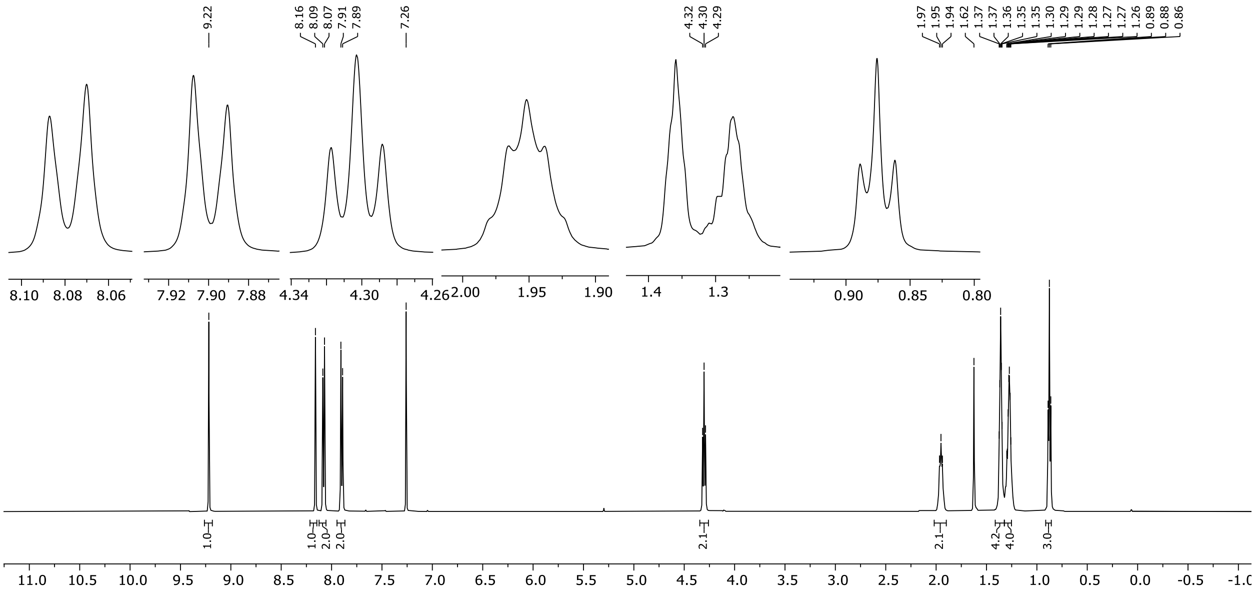

^{13}C-NMR (126 MHz, $CDCl_3$) spectrum of compound 4d:

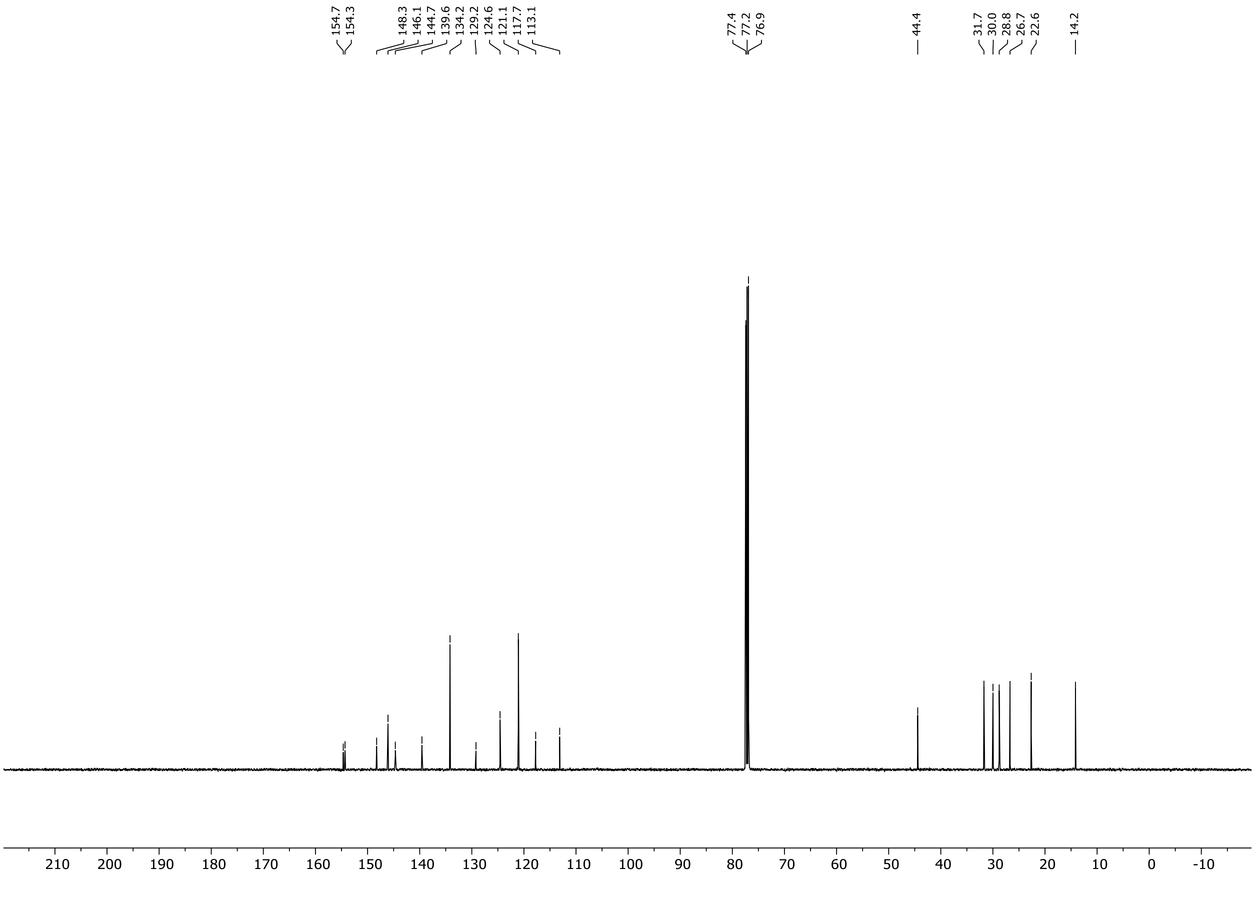

4-[4-(2-Chloro-9-heptyl-9*H*-purin-6-yl)-1*H*-1,2,3-triazol-1-yl]-*N*,*N*-dimethylaniline (4e)

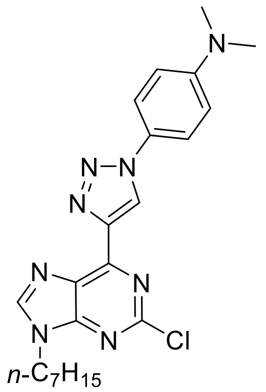

To a solution of compound **I** (922 mg, 2.64 mmol, 1.0 eq.), $CuSO_4 \cdot 5H_2O$ (67 mg, 0.42 mmol, 0.16 eq.) and sodium ascorbate (166 mg, 0.84 mmol, 0.32 eq.) in DMF (33 mL) 10 w-% AcOH solution (10.5 mL) and 4-azido-*N*,*N*-dimethylaniline [68] (26.4 mL, C = 0.15 M (in DCM), 3.96 mmol, 1.5 eq.) were added and the reaction mixture was stirred isolated from the daylight for 1 h at 70 °C temperature. Then, it was evaporated and dried in a vacuum. Water (30 mL) was added, and the organic phase was extracted with DCM (2×20 mL). The combined organic phase was washed with aqueous NaHS (2×40 mL) and sat. NaCl (2×40 mL), dried over anhydrous Na_2SO_4, filtered and evaporated. Silica gel column chromatography (DCM/EtOH, gradient 0% → 4%) provided product **4e** (yield: 0.94 g, 81%) as a light brown amorphous solid. R_f = 0.55 (DCM/EtOH = 20:1). HPLC: t_R = 7.09 min, eluent E_2. IR (neat) ν (cm^{-1}): 2926, 2854, 1592, 1530, 1358, 1331, 1248, 1158, 1090, 1038, 1002, 943, 855, 832. ^{1}H-NMR (500 MHz, $CDCl_3$) δ (ppm): 8.98 (s, 1H, H-C(triazole)), 8.13 (s, 1H, H-C(purine)), 7.67 (d, 2H, ^3J = 8.7 Hz, 2×H-C(Ar)), 6.79 (d, 2H, ^3J = 8.7 Hz, 2×H-C(Ar)), 4.29 (t, 2H, ^3J = 7.0 Hz, (-CH_2-)), 3.04 (s, 6H, (-NMe_2)), 1.98–1.90 (m, 2H, (-CH_2-)), 1.39–1.32 (m, 4H, 2×(-CH_2-)), 1.32–1.21 (m, 4H, 2×(-CH_2-)), 0.87 (t, 3H, ^3J = 7.0 Hz, (-CH_3)). ^{13}C-NMR (126 MHz, $CDCl_3$) δ (ppm): 154.6, 154.1, 151.0, 149.4, 145.6, 143.5, 129.0, 126.3, 124.7, 122.3, 112.4, 44.3, 40.6, 31.7, 30.0, 28.8, 26.7, 22.7, 14.2. HRMS (ESI) m/z: $[M+H]^+$ Calcd for $C_{22}H_{28}ClN_8$ 439.2120; Found 439.2117 (0.68 ppm).

^{1}H-NMR (500 MHz, $CDCl_3$) spectrum of compound 4e:

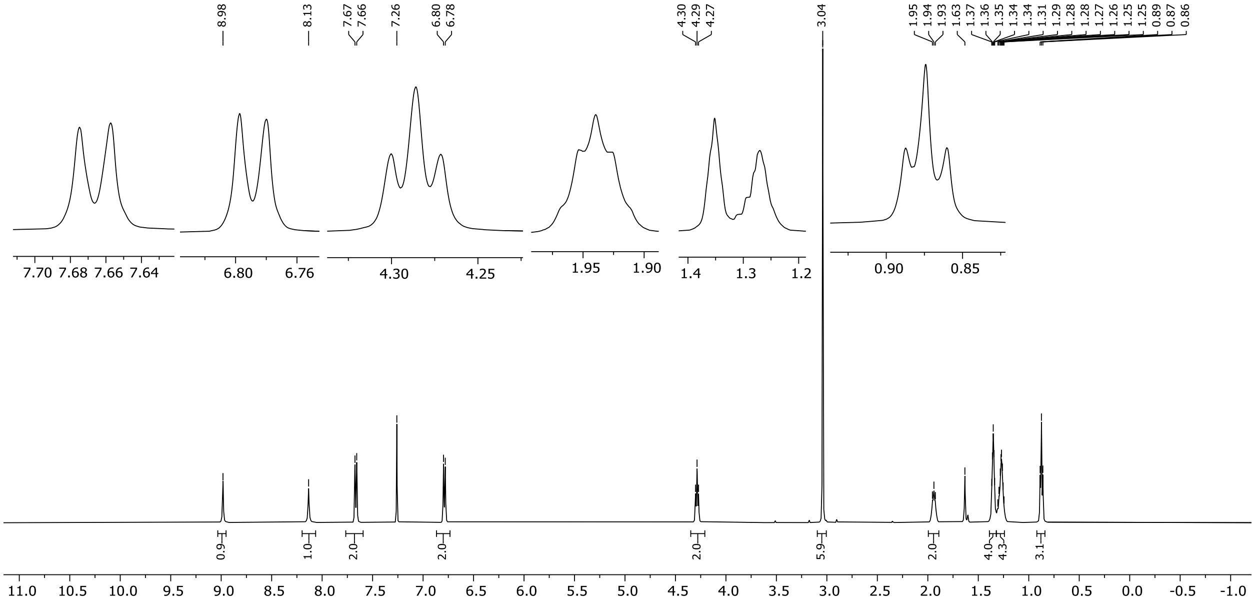

^{13}C-NMR (126 MHz, $CDCl_3$) spectrum of compound 4e:

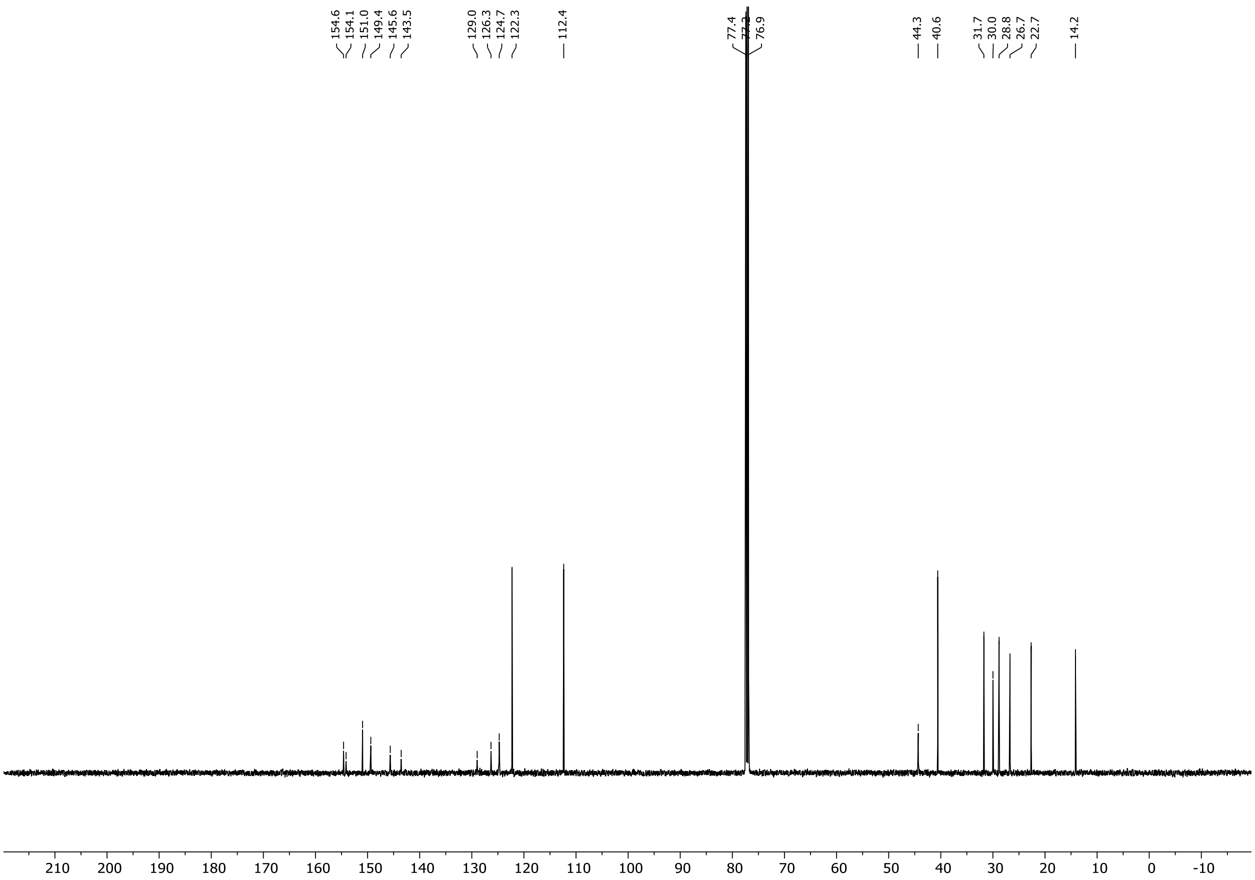

5. Synthesis of 6-triazolyl-9-heptylpurines 4f-g

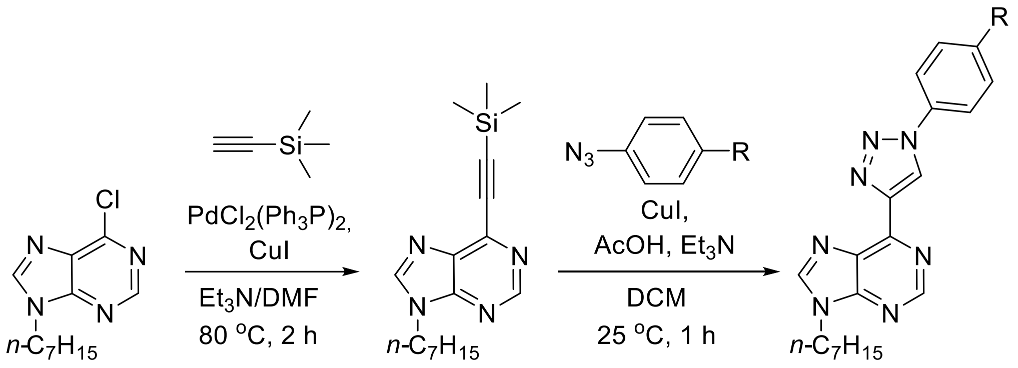

II **III** **4f-g**

Scheme S2. Synthesis of 6-triazolyl-9-heptylpurines **4f-g**

6-Chloro-9-heptyl-9*H*-purine (II)

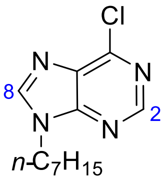

In an inert atmosphere to 6-chloropurine (5.0 g, 32.35 mmol, 1.0 eq.), *n*-heptanol (5.5 mL, ρ = 0.82 g/cm^3, 38.82 mmol, 1.2 eq.), and Ph_3P (11.02 g, 42.06 mmol, 1.3 eq.) dry THF (70 mL) was added and the reaction mixture was cooled to 0 °C temperature. Then DIAD (8.26 mL, ρ = 1.03 g/cm^3, 42.06 mmol, 1.3 eq.) was slowly added dropwise over 1 h, and the reaction mixture was stirred for 1 h at 20 °C temperature. Then it was evaporated to dryness and EtOH (20 mL) was added, and the resulting mixture was left at −10 °C temperature for 18 h to form a precipitate of Ph_3PO, which was filtered out, and the filtrate was evaporated. Reverse phase silica gel column chromatography (MeOH/H_2O, gradient 50 → 95%) provided product **II** (yield: 6.37 g, 71%) as a slightly yellow oil. R_f = 0.30 (Tol/MeCN = 5:1). HPLC: t_R = 5.92 min, eluent E_2. IR (neat) ν (cm^{-1}): 2927, 2857, 1591, 1557, 1401, 1331, 1212, 1178, 1145, 939, 857, 792. ^{1}H-NMR (500 MHz, $CDCl_3$) δ (ppm): 8.75 (s, 1H, H-C(purine C2)),[1] 8.12 (s, 1H, H-C(purine C8)),[1] 4.29 (t, 2H, ^3J = 7.3 Hz, (-CH_2-)), 1.92 (q, 2H, ^3J = 7.3 Hz, (-CH_2-)), 1.38–1.30 (m, 8H, 4×(-CH_2-)), 0.86 (t, 3H, ^3J = 7.3 Hz, (-CH_3)). ^{13}C-NMR (126 MHz, $CDCl_3$) δ (ppm): 152.04, 152.01, 151.2, 145.2, 131.8, 44.7, 31.7, 30.0, 28.8, 26.7, 22.6, 14.1. HRMS (ESI) m/z: [M+H]$^+$ Calcd for $C_{12}H_{18}ClN_4$ 253.1215; Found 253.1212 (1.19 ppm).

[1] The signal was assigned from ^{1}H-^{13}C HSQC and ^{1}H-^{13}C HMBC spectra.

^{1}H-NMR (500 MHz, $CDCl_3$) spectrum of compound II:

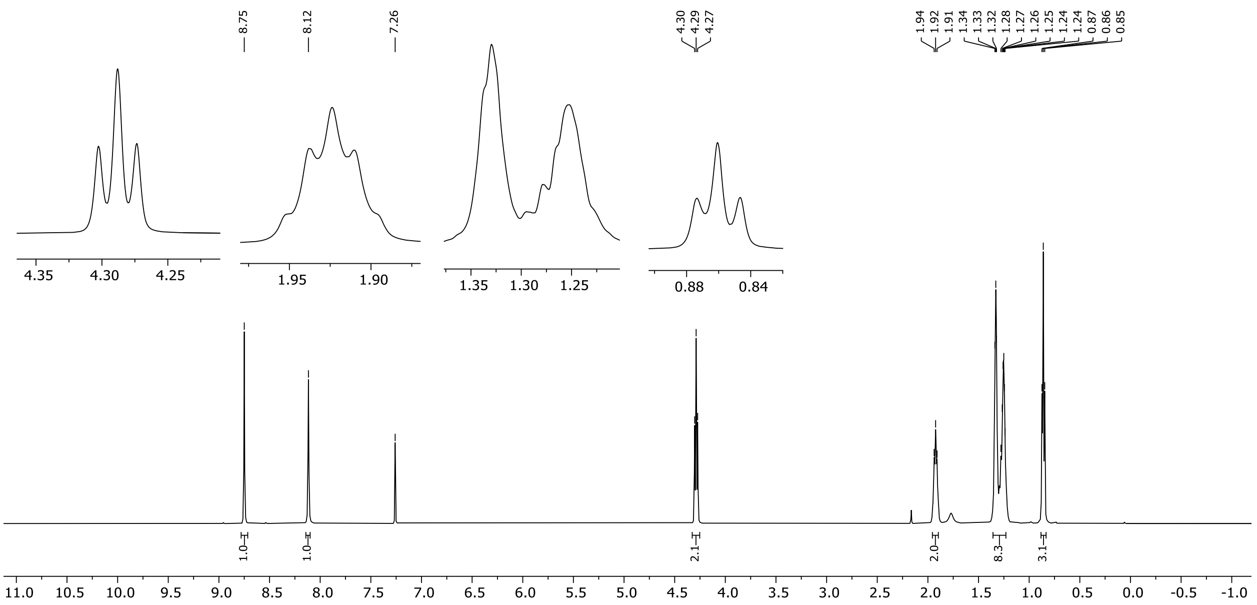

^{13}C-NMR (126 MHz, $CDCl_3$) spectrum of compound II:

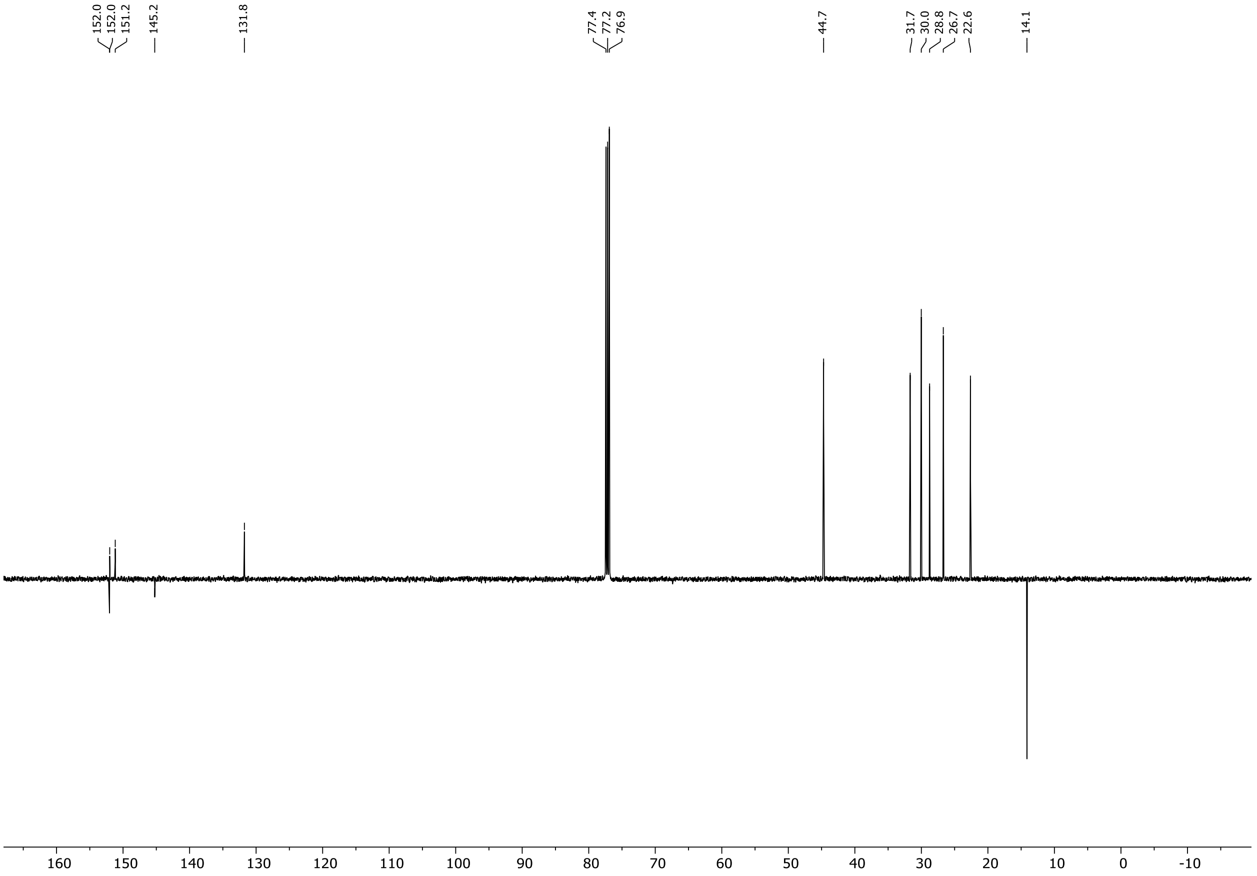

^{1}H-^{13}C HSQC spectrum of compound II

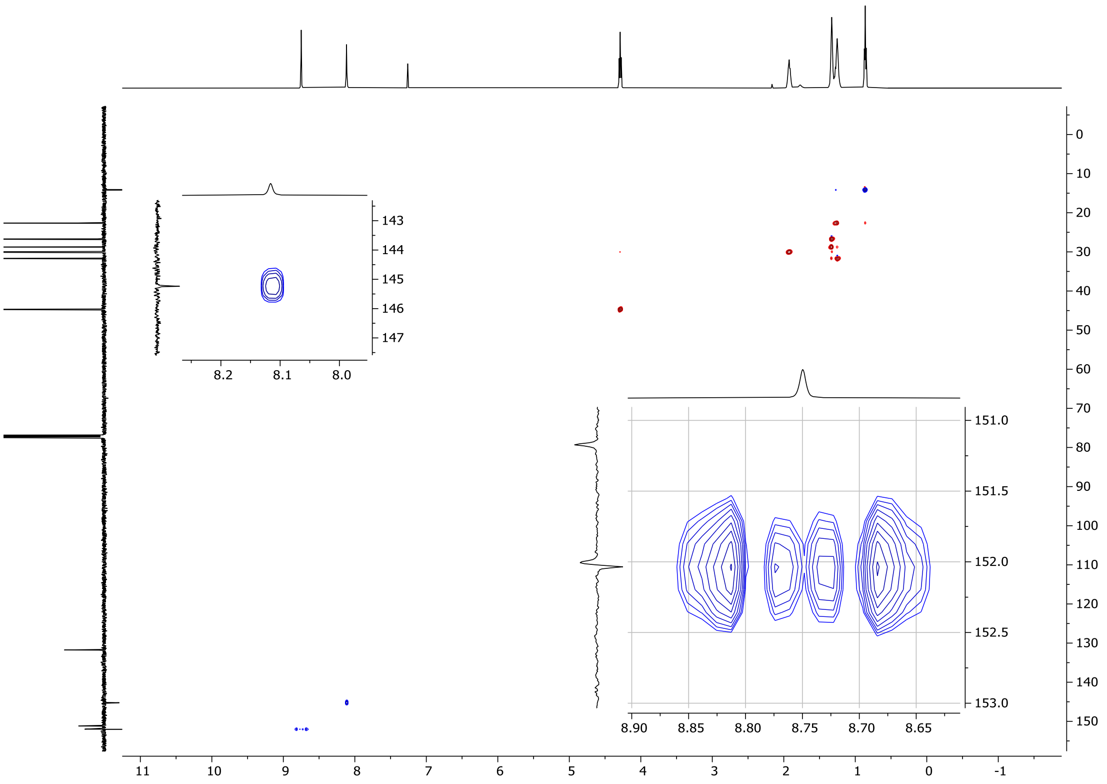

^{1}H-^{13}C HMBC spectrum of compound II

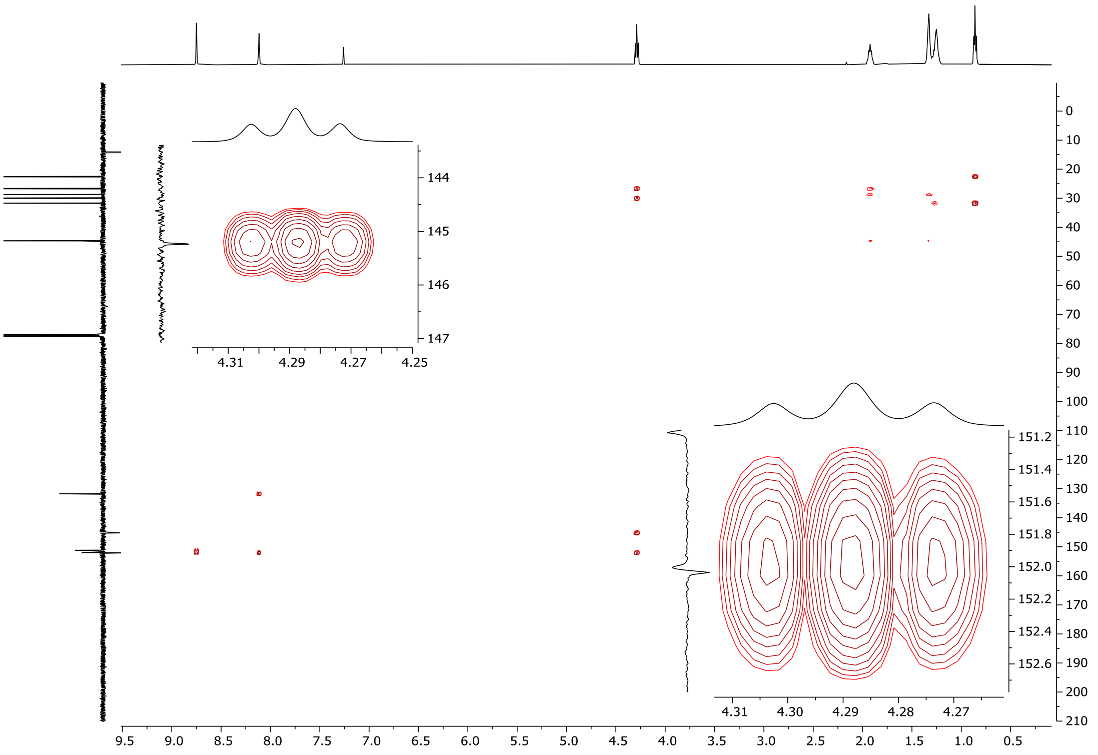

9-Heptyl-6-[(trimethylsilyl)ethynyl]-9*H*-purine (III)

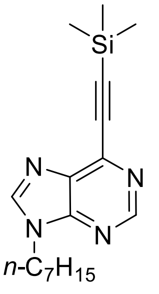

To a solution of compound **II** (500 mg, 1.98 mmol, 1.0 eq.), $PdCl_2(Ph_3P)_2$ (126 mg, 0.18 mmol, 0.09 eq.), CuI (57 mg, 0.30 mmol, 0.15 eq.) in dry DMF/Et_3N mixture (1:1, 4 mL) trimethylsilylacetylene (0.42 mL, ρ = 0.69 g/cm^3, 2.97 mmol, 1.5 eq.) was added and the reaction mixture was stirred at 80 ^oC temperature for 2 h. Then it was evaporated to dryness, and the reaction mixture was suspended in DCM (10 mL) and washed with 5 % aqueous LiCl solution (2×10 mL), 20 % aqueous NaHS solution (10 mL), and brine (10 mL). The organic phase was dried over anhydrous Na_2SO_4, filtered, and evaporated. Silica gel column chromatography (DCM/EtOH, gradient 0% → 5%) provided product **III** (yield: 356 mg, 57%) as a dark red oil. R_f = 0.66 (DCM/EtOH = 20:1). HPLC: t_R = 7.27 min, eluent E_2. IR (neat) ν (cm^{-1}): 2956, 2928, 2857, 1575, 1436, 1401, 1325, 1249, 1196, 1002, 841, 760, 641. ^{1}H-NMR (500 MHz, $CDCl_3$) δ (ppm): 8.91 (s, 1H, H-C(purine)), 8.11 (s, 1H, H-C(purine)), 4.27 (t, 2H, ^3J = 7.3 Hz, (-CH_2-)), 1.90 (q, 2H, ^3J = 7.3 Hz, (-CH_2-)), 1.32–1.20 (m, 8H, 4×(-CH_2-)), 0.85 (t, 3H, ^3J = 7.3 Hz, (-CH_3)), 0.32 (s, 9H, 3×(-CH_3)). ^{13}C-NMR (126 MHz, $CDCl_3$) δ (ppm): 152.4, 151.8, 145.5, 141.1, 134.3, 105.3, 98.5, 44.1, 31.6, 29.9, 28.6, 26.6, 22.5, 14.0, -0.4. HRMS (ESI) m/z: [M+H]$^+$ ^{f}Calcd for $C_{17}H_{27}N_4Si$ 315.1999; Found 315.1997 (0.63 ppm).

^{1}H-NMR (500 MHz, $CDCl_3$) spectrum of compound III:

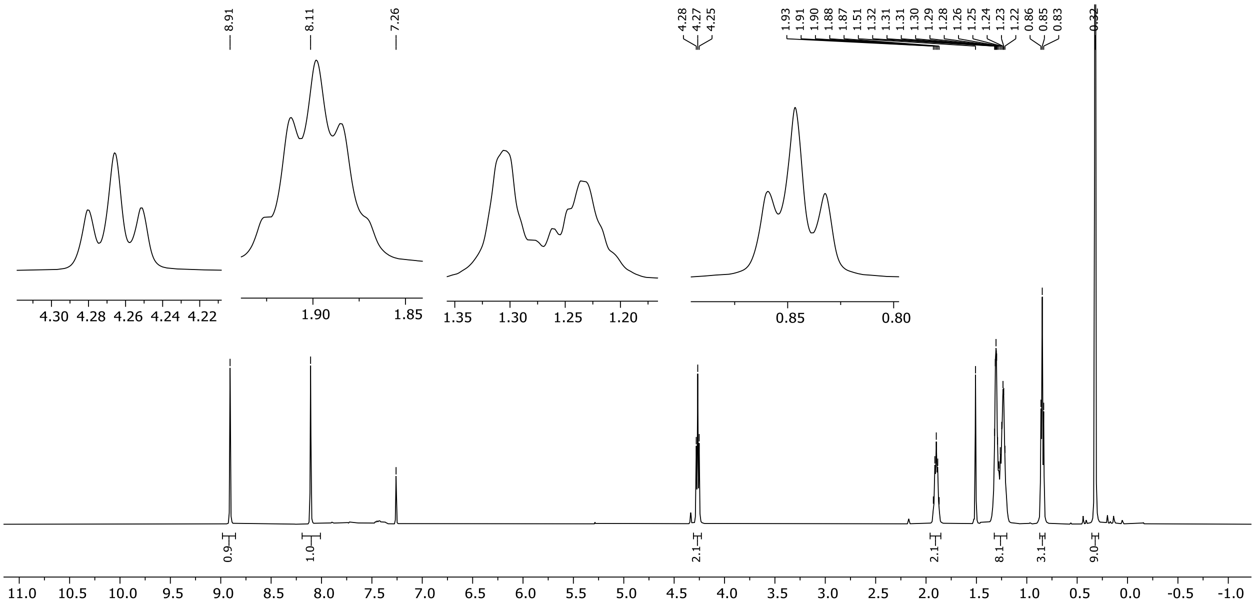

^{13}C-NMR (126 MHz, $CDCl_3$) spectrum of compound III:

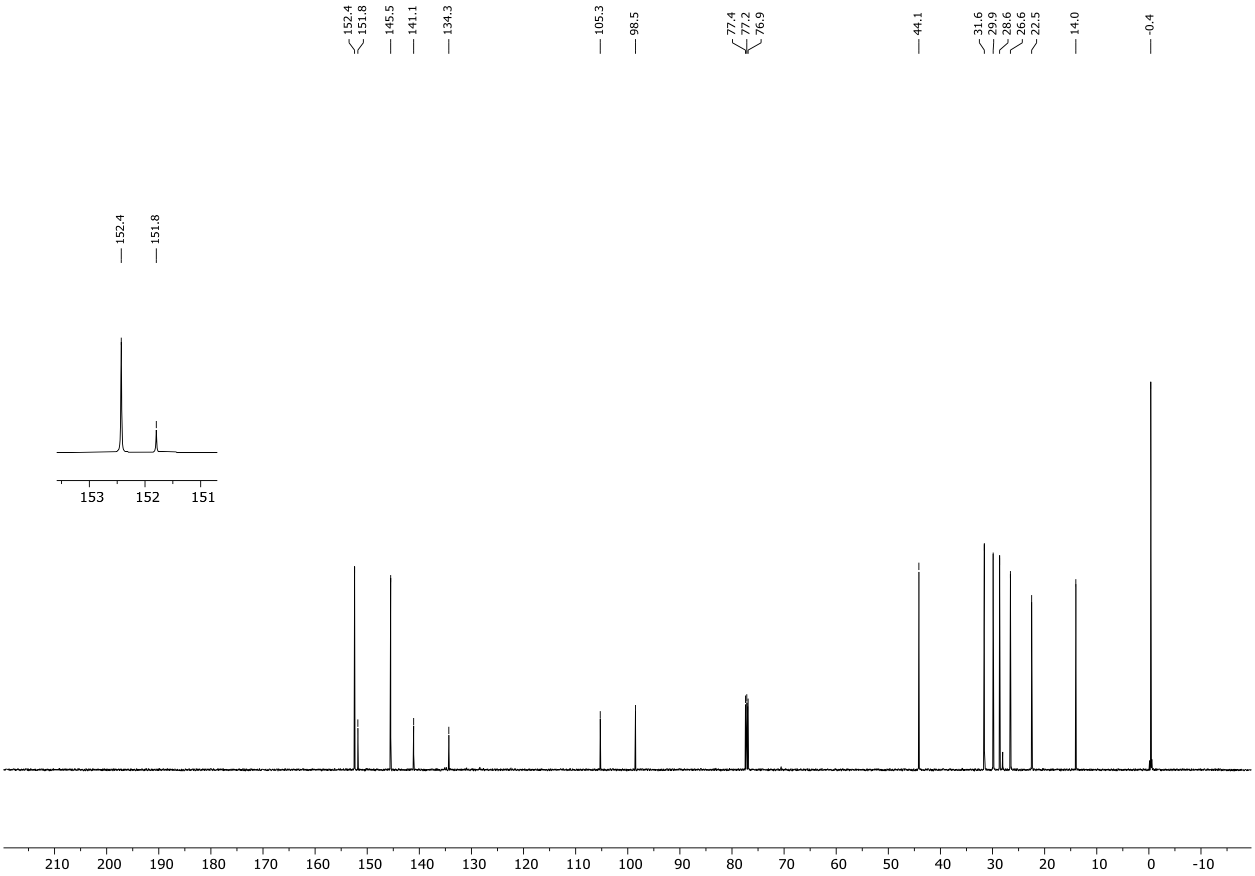

9-Heptyl-6-(1-phenyl-1*H*-1,2,3-triazol-4-yl)-9*H*-purine (4f)

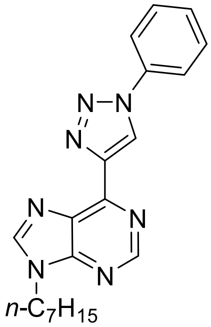

Prepared according to general method D: Compound **III** (578 mg, 1.83 mmol, 1.0 eq.), CuI (56 mg, 0.29 mmol, 0.16 eq.), AcOH (115 µL, ρ = 1.05 g/cm^3, 2.01 mmol, 1.1 eq.), Et_3N (0.28 mL, ρ = 0.73 g/cm^3, 2.01 mmol, 1.1 eq.) and azidobenzene [47] (7.24 mL, C = 0.38 M (in DCM), 2.75 mmol, 1.5 eq.), DMF (50 mL), 25 °C, 1 h. Silica gel column chromatography (DCM/EtOH, gradient 0% → 3%) provided product **4f** (yield: 505 mg, 65%) as a light brown amorphous solid. R_f = 0.54 (DCM/EtOH = 20:1). HPLC: t_R = 6.01 min, eluent E_2. IR (neat) ν (cm^{-1}): 2920, 2854, 1586, 1508, 1465, 1323, 1251, 1145, 1037, 849, 806, 755, 697, 643. ^{1}H-NMR (500 MHz, $CDCl_3$) δ (ppm): 9.22 (s, 1H, H-C(triazole)), 9.09 (s, 1H, H-C(purine)), 8.15 (s, 1H, H-C(purine)), 7.90 (d, 2H, ^3J = 7.8 Hz, 2×H-C(Ph)), 7.58 (t, 2H, ^3J = 7.8 Hz, 2×H-C(Ph)), 7.49 (t, 1H, ^3J = 7.8 Hz, H-C(Ph)), 4.34 (t, 2H, ^3J = 7.3 Hz, (-CH_2-)), 2.01–1.93 (m, 2H, (-CH_2-)), 1.40–1.32 (m, 4H, 2×(-CH_2-)), 1.32–1.22 (m, 4H, 2×(-CH_2-)), 0.87 (t, 3H, ^3J = 7.3 Hz, (-CH_3)). ^{13}C-NMR (126 MHz, $CDCl_3$) δ (ppm): 153.0, 152.4, 147.2, 145.0, 144.5, 137.0, 130.1, 130.0, 129.3, 124.8, 121.0, 44.3, 31.7, 30.1, 28.8, 26.8, 22.6, 14.2. HRMS (ESI) m/z: [M+H]$^+$ Calcd for $C_{20}H_{24}N_7$ 362.2088; Found 362.2086 (0.55 ppm).

^{1}H-NMR (500 MHz, $CDCl_3$) spectrum of compound 4f:

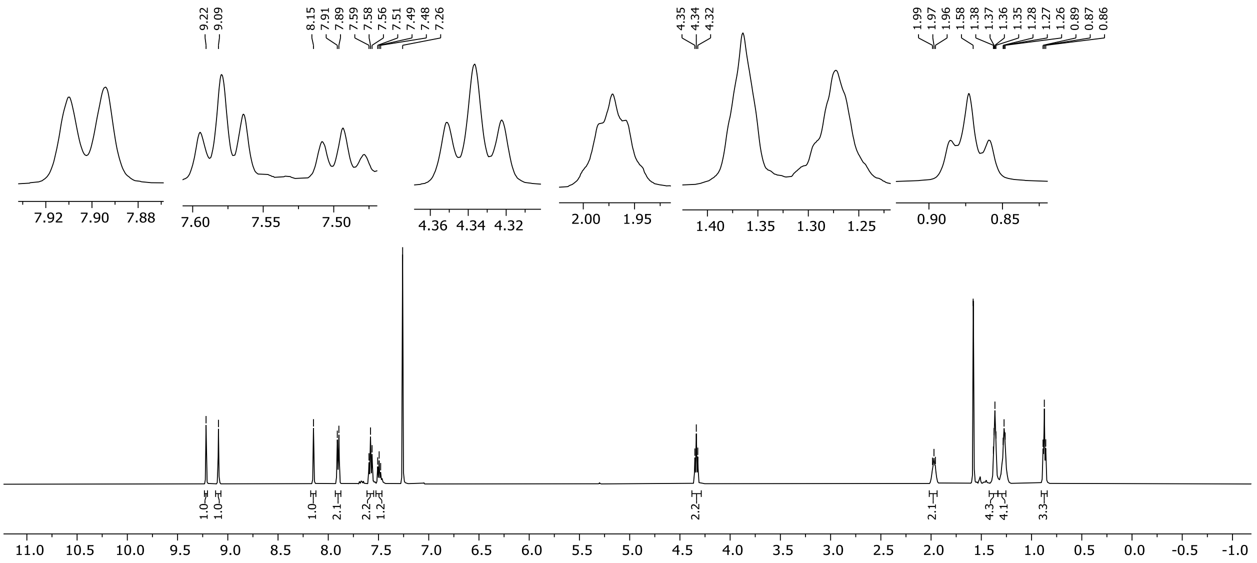

^{13}C-NMR (126 MHz, $CDCl_3$) spectrum of compound 4f:

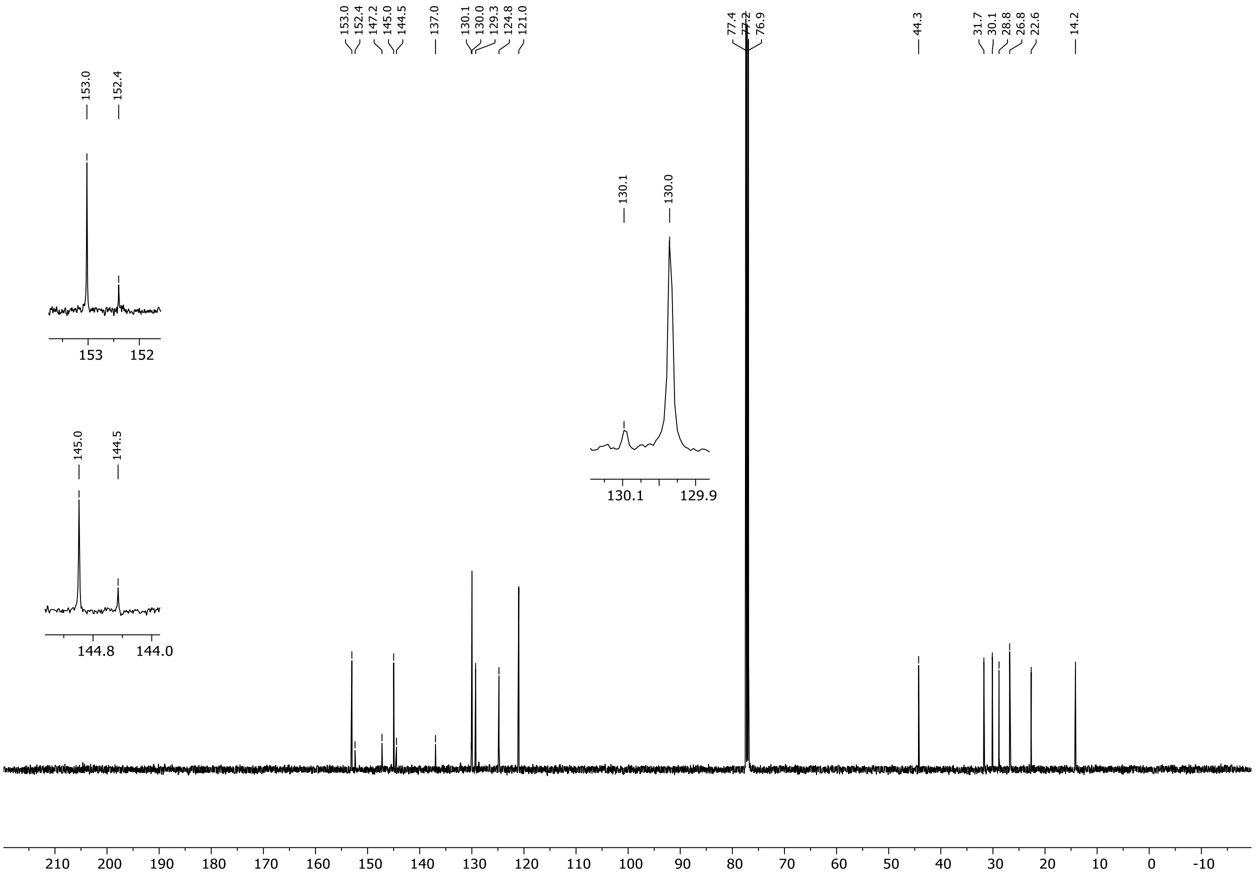

9-Heptyl-6-[1-(4-nitrophenyl)-1*H*-1,2,3-triazol-4-yl]-9*H*-purine (4g)

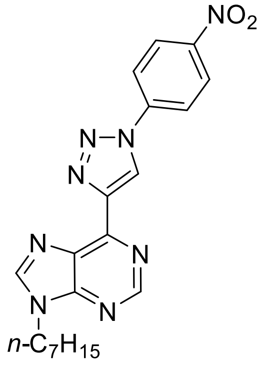

Prepared according to general method D: Compound **III** (611 mg, 1.94 mmol, 1.0 eq.), CuI (59 mg, 0.31 mmol, 0.16 eq.), AcOH (0.12 mL, ρ = 1.05 g/cm^3, 2.13 mmol, 1.1 eq.), Et_3N (0.30 mL, ρ = 0.73 g/cm^3, 2.13 mmol, 1.1 eq.) and 1-azido-4-nitrobenzene [47] (468 mg, 2.85 mmol, 1.5 eq.), DCM (14 mL), 25 °C, 1 h. Silica gel column chromatography (DCM/EtOH, gradient 0% → 4%) provided product **4g** (yield: 466 mg, 59%) as a brown amorphous solid. R_f = 0.46 (DCM/EtOH = 20:1). HPLC: t_R = 6.06 min, eluent E_2. IR (neat) ν (cm^{-1}): 2927, 2855, 1593, 1505, 1340, 1323, 1239, 1141, 1037, 851, 747. ^{1}H-NMR (500 MHz, $CDCl_3$) δ (ppm): 9.34 (s, 1H, H-C(triazole)), 9.11 (s, 1H, H-C(purine)), 8.48 (d, 2H, ^3J = 8.8 Hz, 2×H-C(Ar)), 8.17 (s, 1H, H-C(purine)), 8.16 (d, 2H, ^3J = 8.8 Hz, 2×H-C(Ar)), 4.35 (t, 2H, ^3J = 7.1 Hz, (-CH_2-)), 2.02–1.94 (m, 2H, (-CH_2-)), 1.41–1.32 (m, 4H, 2×(-CH_2-)), 1.32–1.21 (m, 4H, 2×(-CH_2-)), 0.87 (t, 3H, ^3J = 7.1 Hz, (-CH_3)). ^{13}C-NMR (126 MHz, $CDCl_3$) δ (ppm): 153.0, 152.5, 147.6, 146.4, 145.3, 145.2, 141.0, 130.2, 125.7, 124.5, 120.9, 44.3, 31.7, 30.1, 28.8, 26.8, 22.6, 14.1. HRMS (ESI) m/z: [M+H]$^+$ Calcd for $C_{20}H_{23}N_8O_2$ 407.1938; Found 407.1937 (0.25 ppm).

^{1}H-NMR (500 MHz, $CDCl_3$) spectrum of compound 4g:

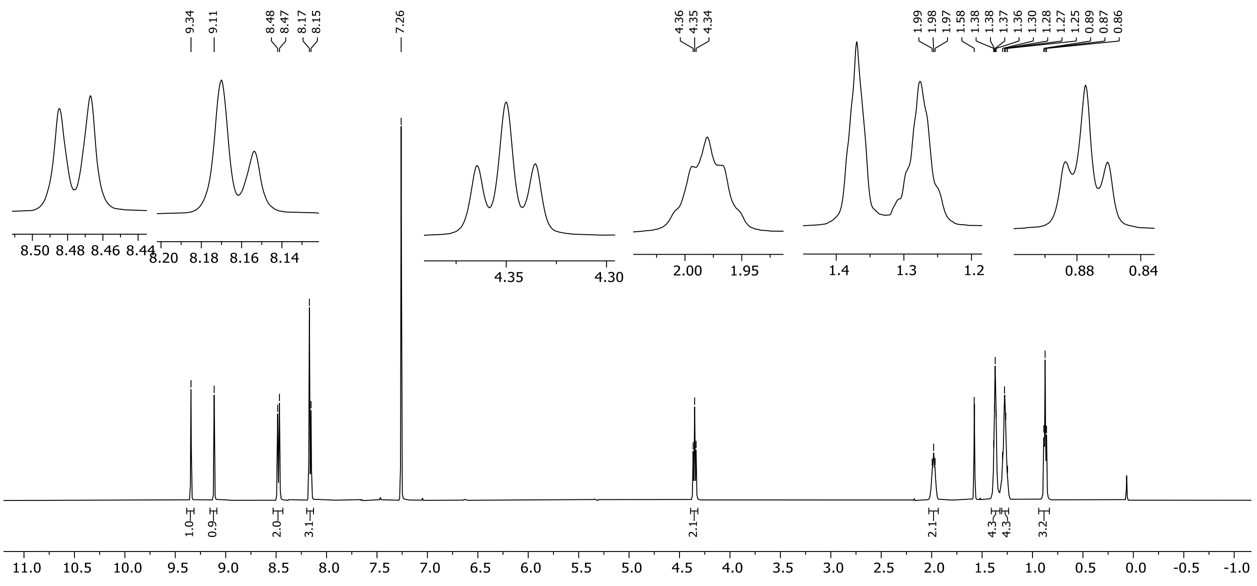

^{13}C-NMR (126 MHz, CDCl$_3$) spectrum of compound 4g:

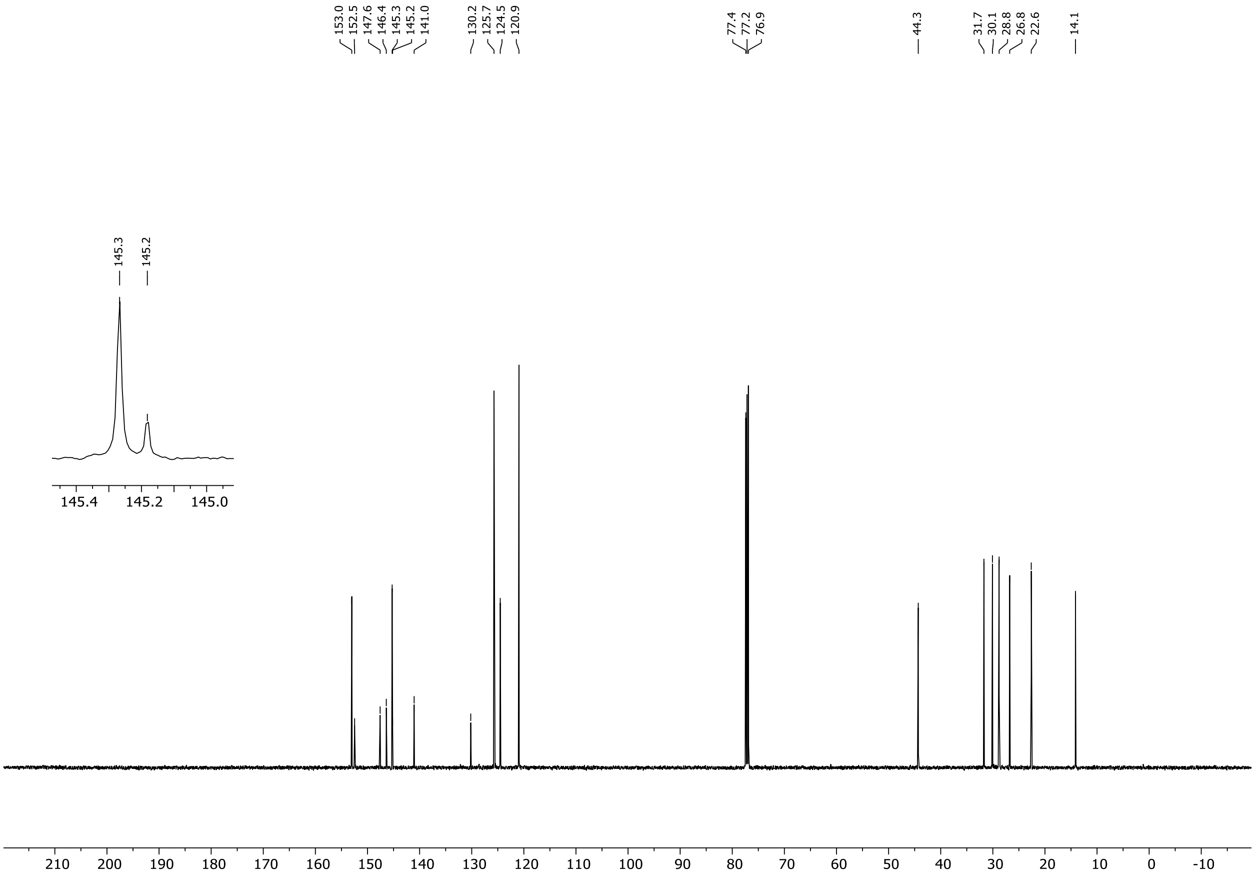

6. Synthesis of 9-heptyl-2,6-bis(1-phenyl-1*H*-1,2,3-triazol-4-yl)-9*H*-purine (4h)

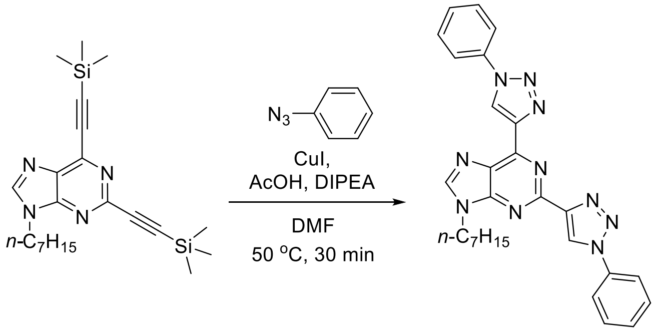

IV **4h**

Scheme S3. Synthesis of 2,6-bistriazolyl-9-heptylpurine **4h**

Prepared according to general method D: Compound **IV** [2] (2.12 g, 5.16 mmol, 1.0 eq.), CuI (295 mg, 1.55 mmol, 0.3 eq.), AcOH (0.65 mL, ρ = 1.05 g/cm^3, 11.35 mmol, 2.2 eq.), DIPEA (1.98 mL, ρ = 0.74 g/cm^3, 11.00 mmol, 2.2 eq.) and azidobenzene [47] (40.7 mL, C = 0.38 M (in DCM), 15.48 mmol, 3.0 eq.), DMF (40 mL), 50 °C, 30 min. Silica gel column chromatography (EtOAc/EtOH, gradient 0% → 4%) provided product **4h** (yield: 1.22 g, 47 %) as a brown amorphous solid. IR (neat) ν (cm^{-1}): 2927, 2858, 1592, 1567, 1503, 1378, 1249, 1212, 1036, 929, 853, 760. ^{1}H-NMR (500 MHz, $CDCl_3$) δ (ppm): 9.28 (s, 1H, H-C(triazole)), 8.96 (s, 1H, H-C(triazole)), 8.17 (s, 1H, H-C(purine)), 7.90 (d, 2H, ^3J = 7.8 Hz, H-C(Ph)), 7.88 (d, 2H, ^3J = 7.8 Hz, H-C(Ph)), 7.57 (t, 2H, ^3J = 7.8 Hz, H-C(Ph)), 7.55 (t, 2H, ^3J = 7.8 Hz, H-C(Ph)), 7.48 (t, 1H, ^3J = 7.8 Hz, H-C(Ph)), 7.46 (t, 1H, ^3J = 7.8 Hz, H-C(Ph)), 4.43 (t, 2H, ^3J = 7.4 Hz, (-CH_2-)), 2.01 (t, 2H, ^3J = 7.4 Hz, (-CH_2-)), 1.44–1.32 (m, 4H, 2×(-CH_2-)), 1.32–1.22 (m, 4H, 2×(-CH_2-)), 0.86 (t, 3H, ^3J = 7.4 Hz, (-CH_3)). ^{13}C-NMR (126 MHz, $CDCl_3$) δ (ppm): 153.4, 153.2, 148.5, 147.4, 145.4, 144.5, 137.1, 136.9, 129.95, 129.91, 129.3, 129.2, 129.1, 125.1, 123.6, 121.0, 120.8, 44.1, 31.7, 30.1, 28.9, 26.8, 22.6, 14.1. HRMS (ESI) m/z: [M+H]$^+$ Calcd for $C_{28}H_{29}N_{10}$ 505.2571; Found 505.2560 (2.18 ppm).

^{1}H-NMR (500 MHz, $CDCl_3$) spectrum of compound 4h:

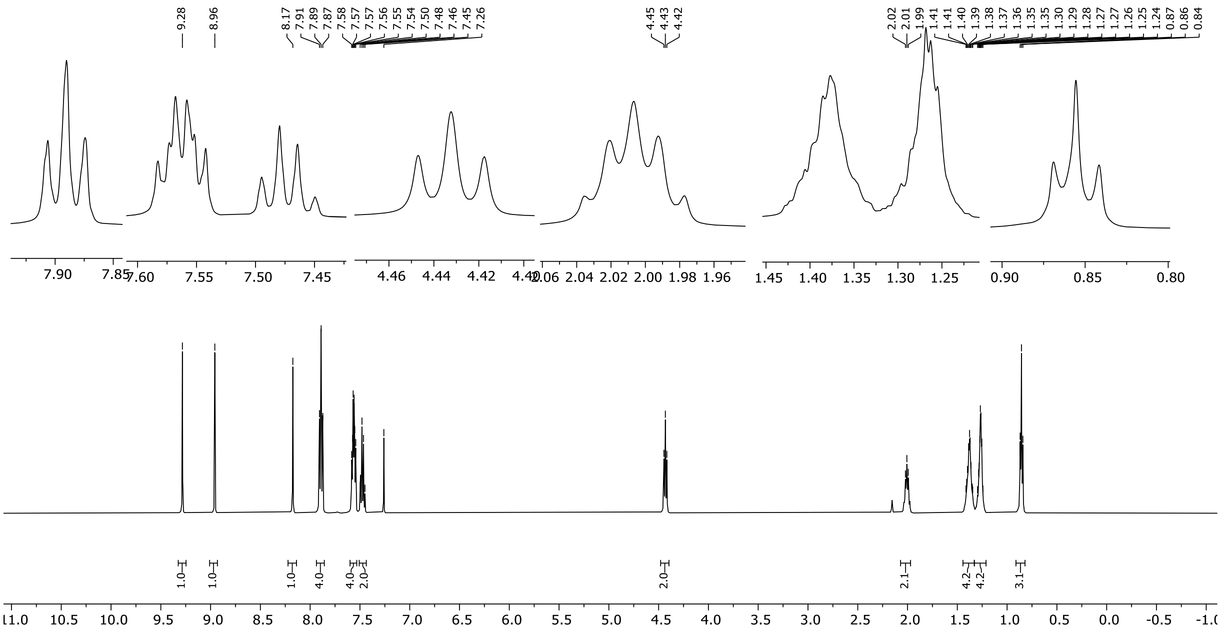

^{13}C-NMR (126 MHz, $CDCl_3$) spectrum of compound 4h:

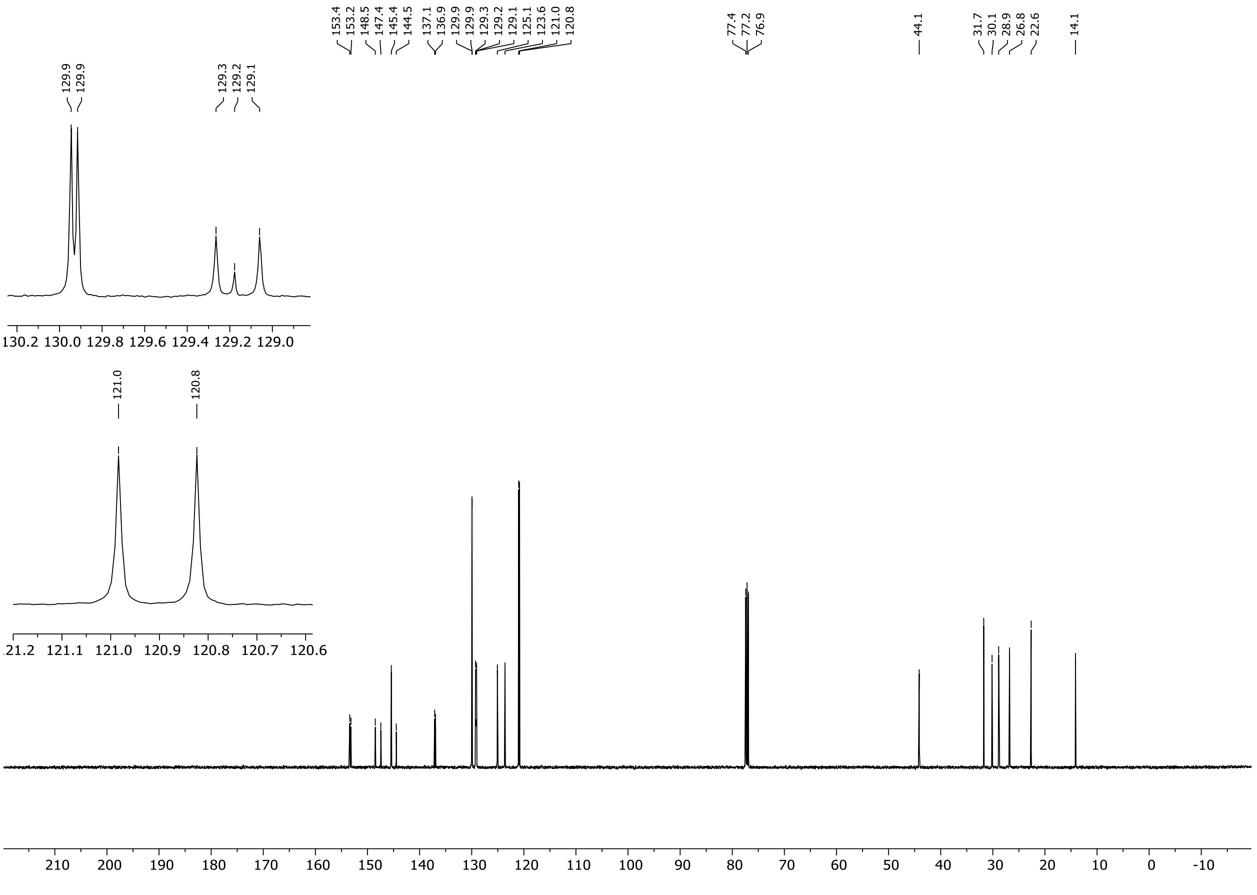

7. Spectral data of compounds 2a, 2b'-d', 3a, 5a-e,h, 6a-d, 6f-g, 7a-b, and 8a-b

Methyl (*E*)-3-(9*H*-carbazol-9-yl)-3-[(4-nitrophenyl)amino]acrylate (2a)

^{1}H-NMR (500 MHz, $CDCl_3$) spectrum of compound 2a:

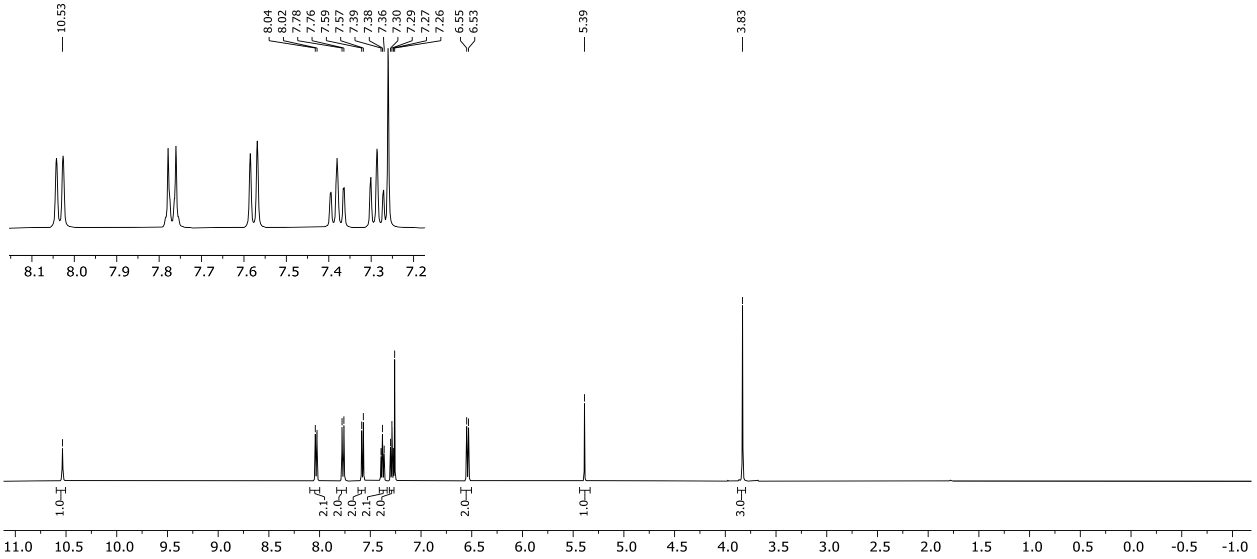

^{13}C-NMR (126 MHz, $CDCl_3$) spectrum of compound 2a:

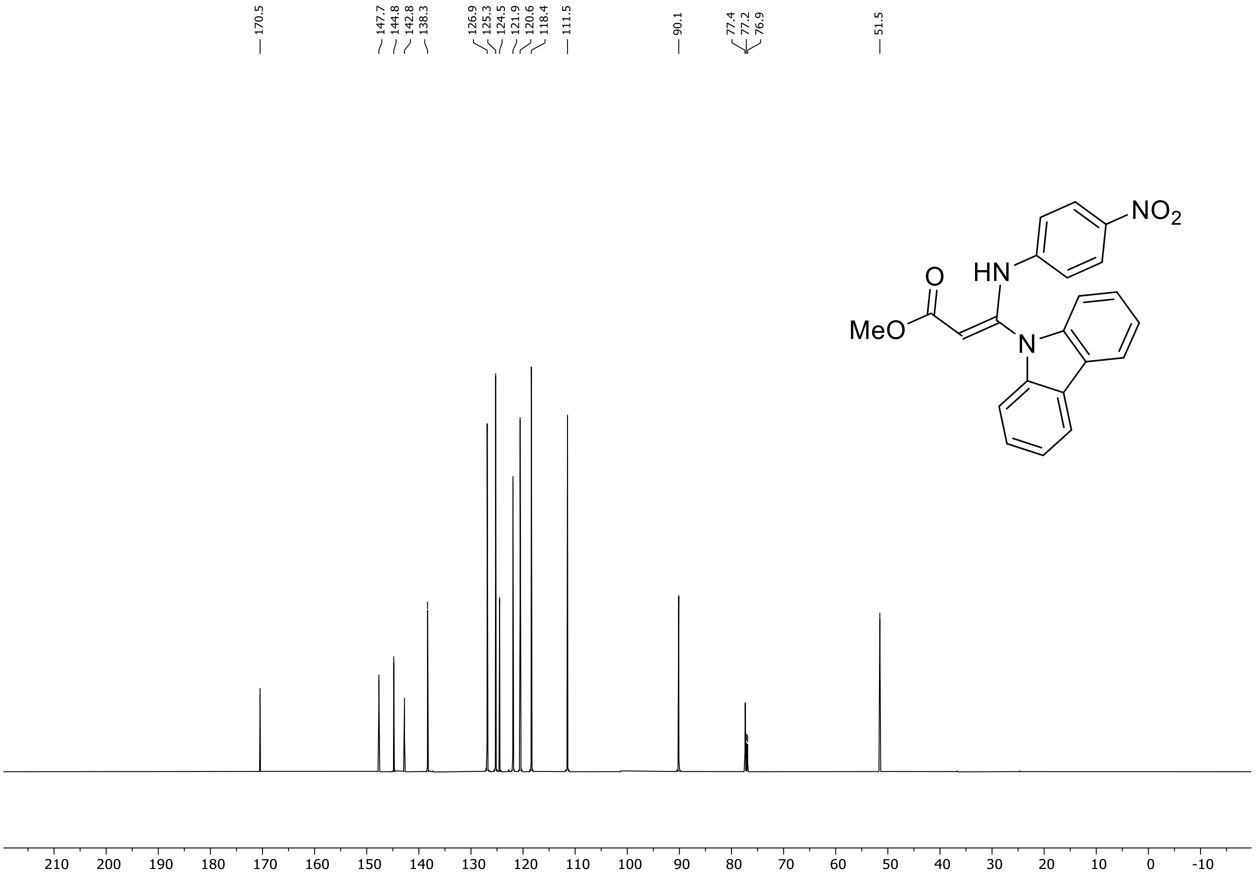

(*E*)-4-(2-(9*H*-Carbazol-9-yl)-2-((4-nitrophenyl)imino)acetyl)benzonitrile (2b')

^{1}H-NMR (500 MHz, $CDCl_3$) spectrum of compound 2b':

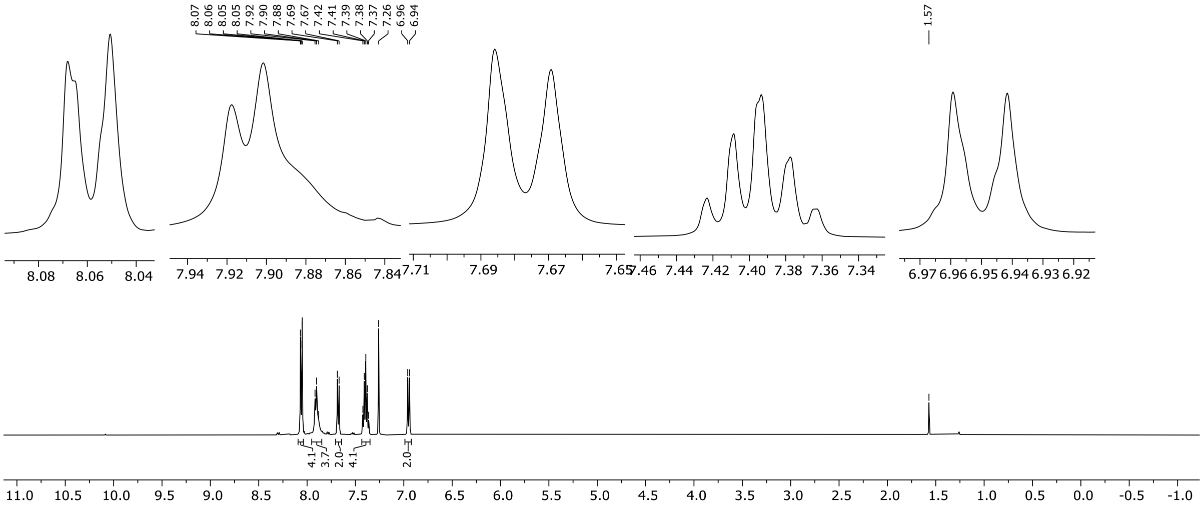

^{13}C-NMR (126 MHz, $CDCl_3$) spectrum of compound 2b':

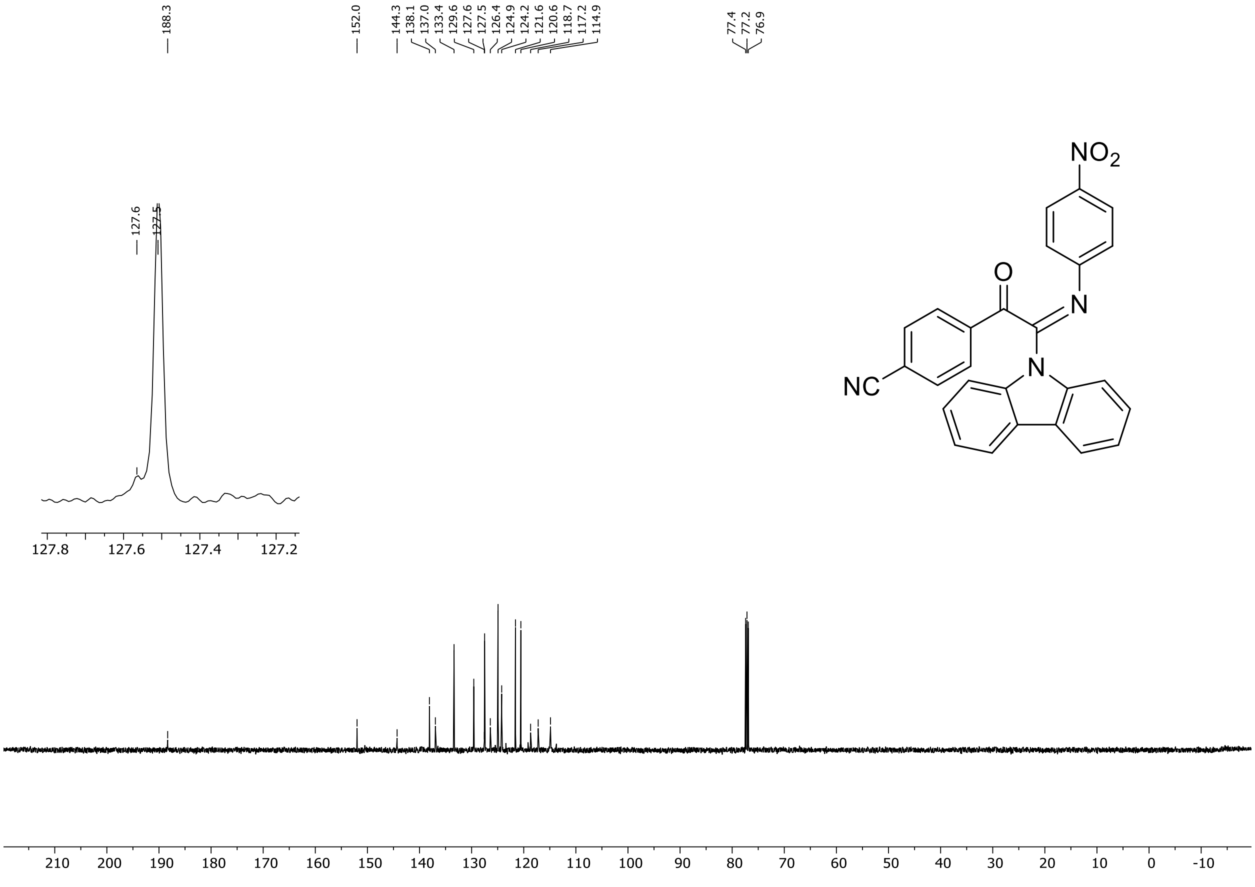

(*E*)-2-(9*H*-Carbazol-9-yl)-2-((4-nitrophenyl)imino)-1-(pyridin-2-yl)ethan-1-one (2c')

^{1}H-NMR (500 MHz, $CDCl_3$) spectrum of compound 2c':

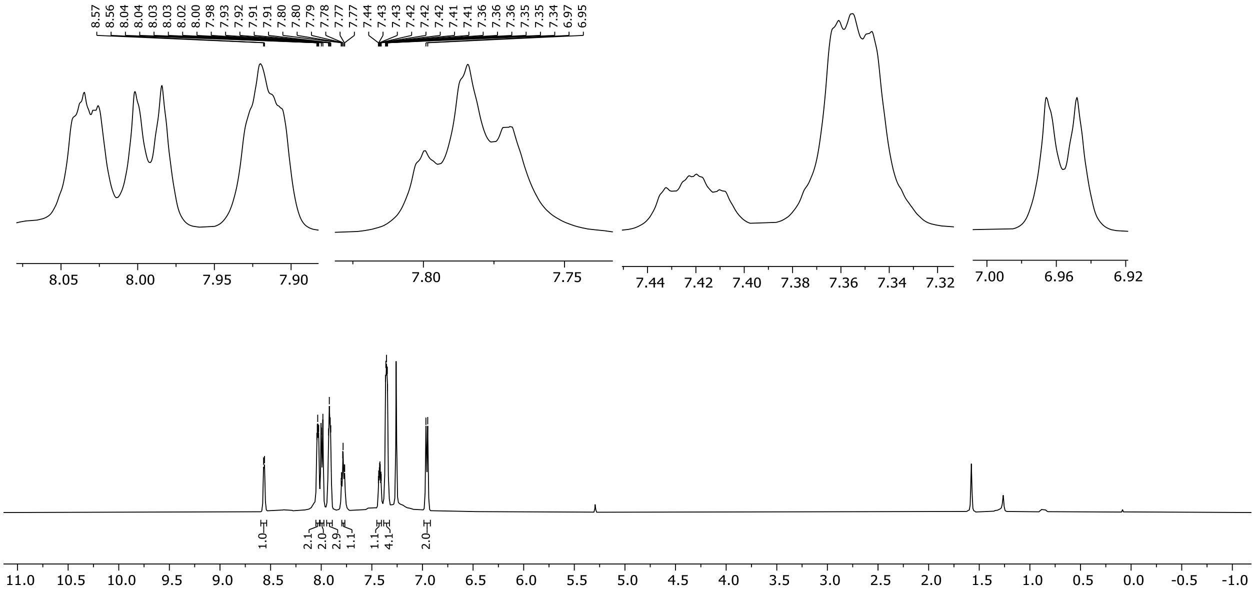

^{13}C-NMR (126 MHz, $CDCl_3$) spectrum of compound 2c':

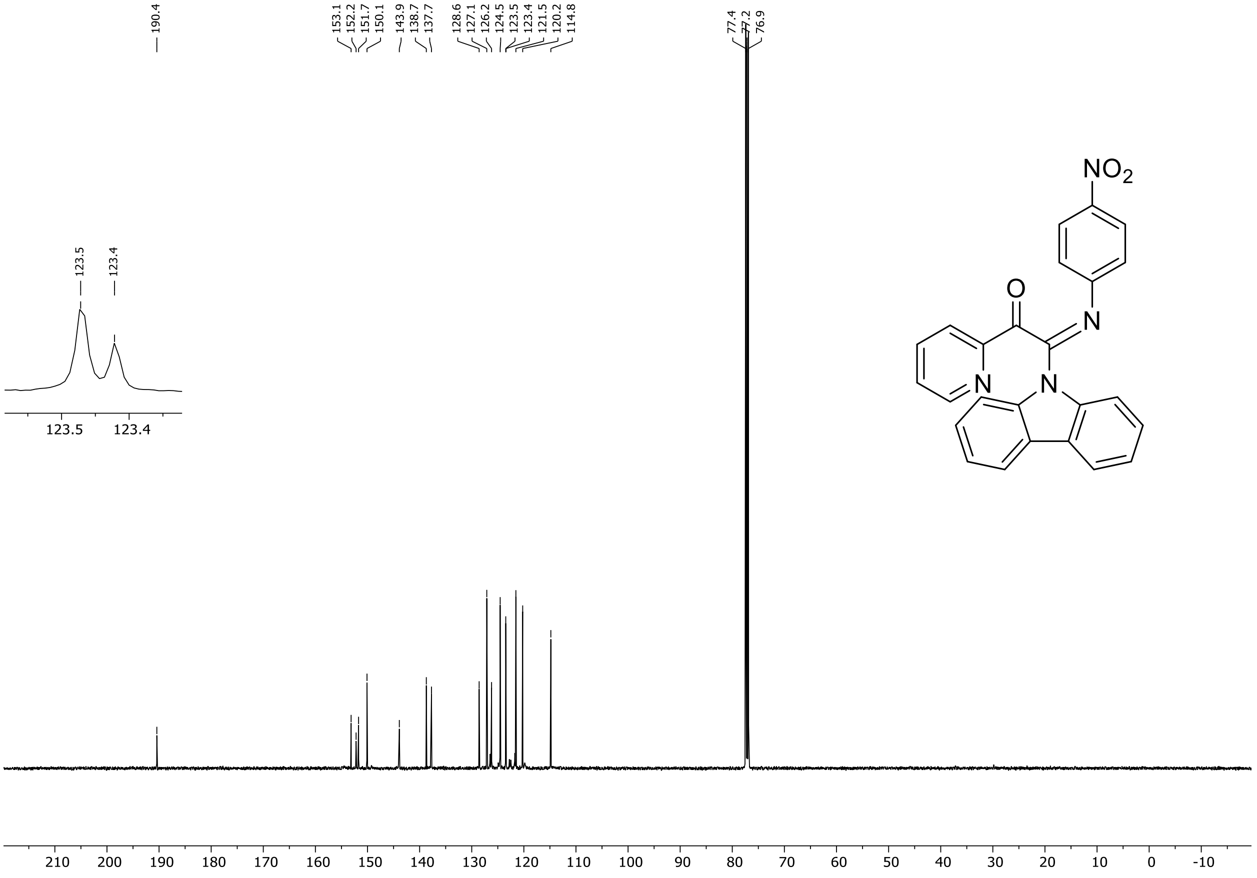

(*E*)-2-(9*H*-Carbazol-9-yl)-2-((4-nitrophenyl)imino)-1-(pyrimidin-5-yl)ethan-1-one (2d’)

^{1}H-NMR (500 MHz, $CDCl_3$) spectrum of compound 2d’:

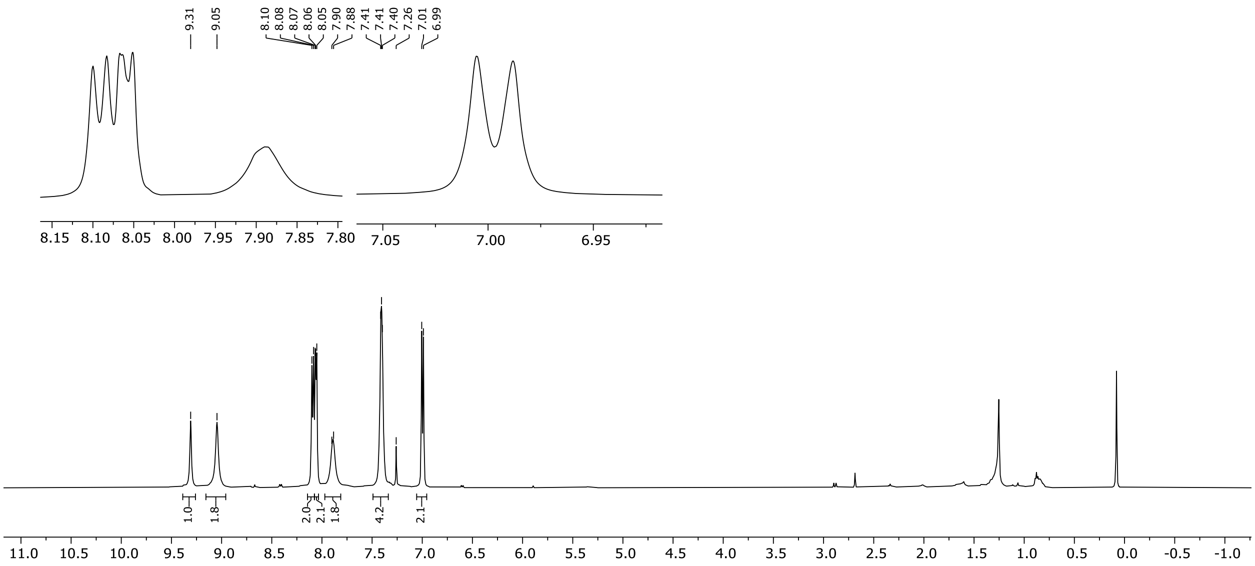

^{13}C-NMR (126 MHz, $CDCl_3$) spectrum of compound 2d’:

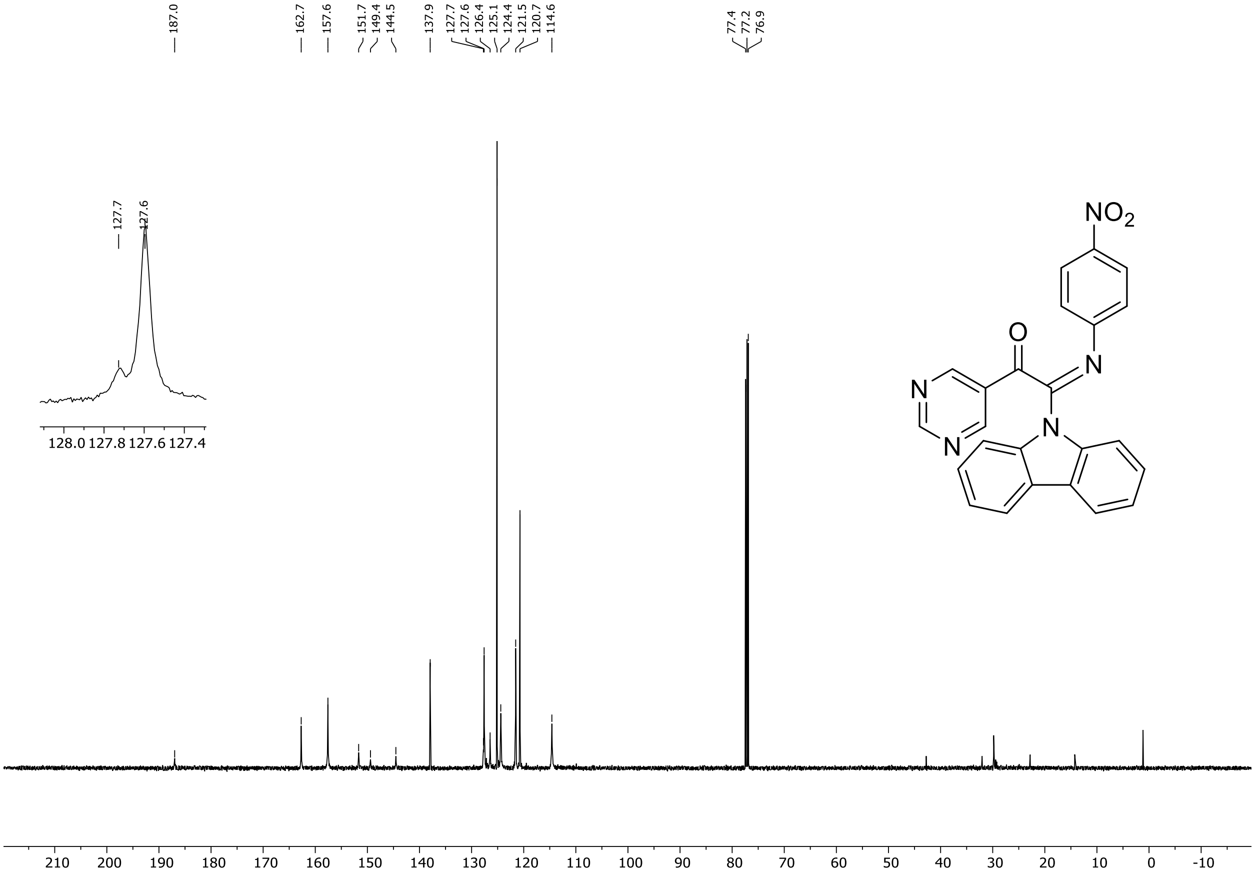

Methyl 2-(9*H*-carbazol-9-yl)-5-nitro-1*H*-indole-3-carboxylate (3a)

^{1}H-NMR (500 MHz, DMSO-d$_6$) spectrum of compound 3a:

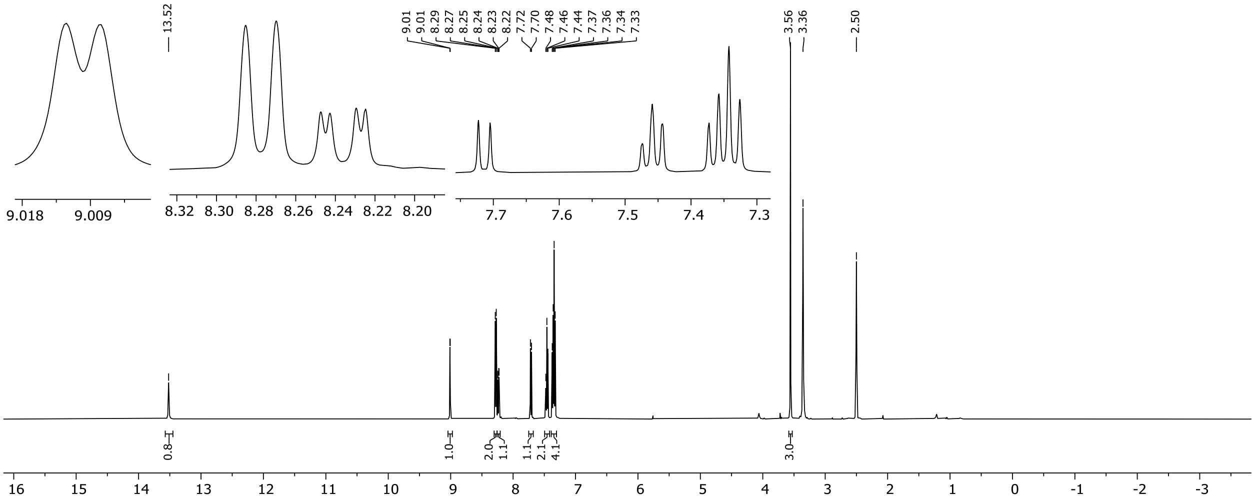

^{13}C-NMR (126 MHz, DMSO-d$_6$) spectrum of compound 3a:

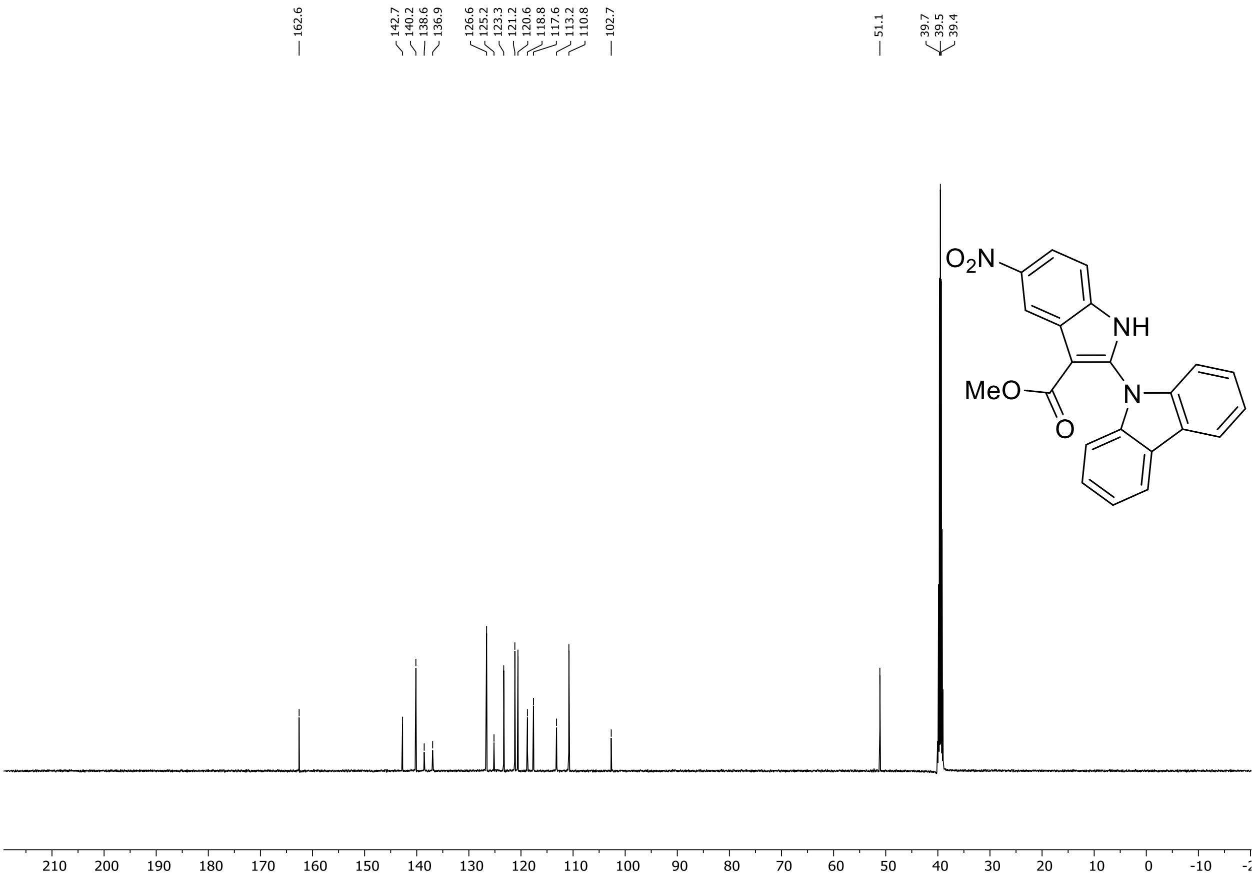

(*E*)-*N*-[1-(9*H*-Carbazol-9-yl)-2-(2-chloro-9-heptyl-9*H*-purin-6-yl)vinyl]aniline (5a)

^{1}H-NMR (500 MHz, $CDCl_3$) spectrum of compound 5a:

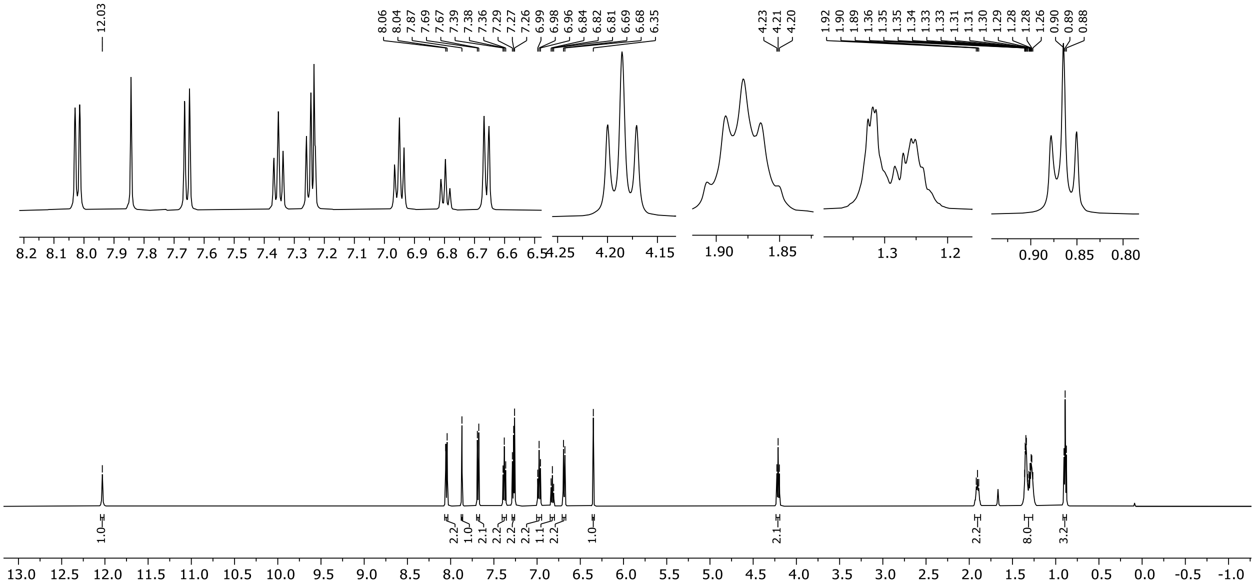

^{13}C-NMR (126 MHz, $CDCl_3$) spectrum of compound 5a:

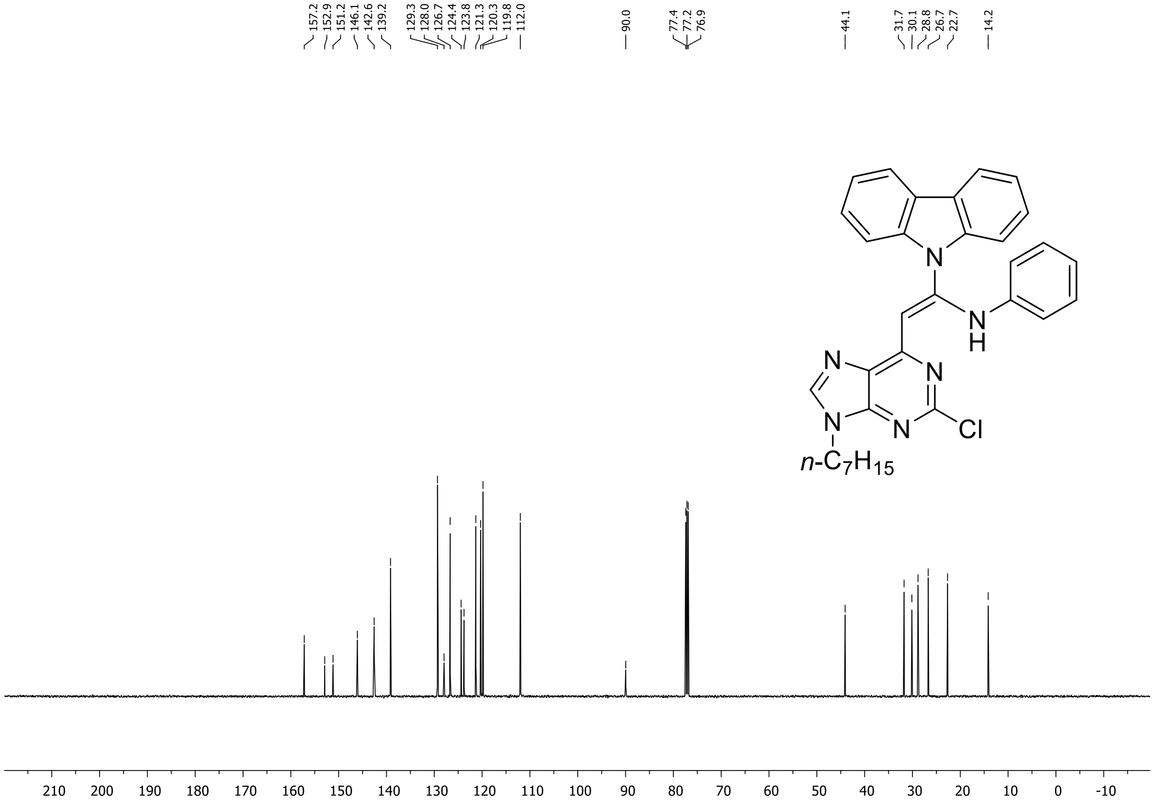

(*E*)-*N*-[1-(9*H*-Carbazol-9-yl)-2-(2-chloro-9-heptyl-9*H*-purin-6-yl)vinyl]-4-nitroaniline (5b)

^{1}H-NMR (500 MHz, $CDCl_3$) spectrum of compound 5b:

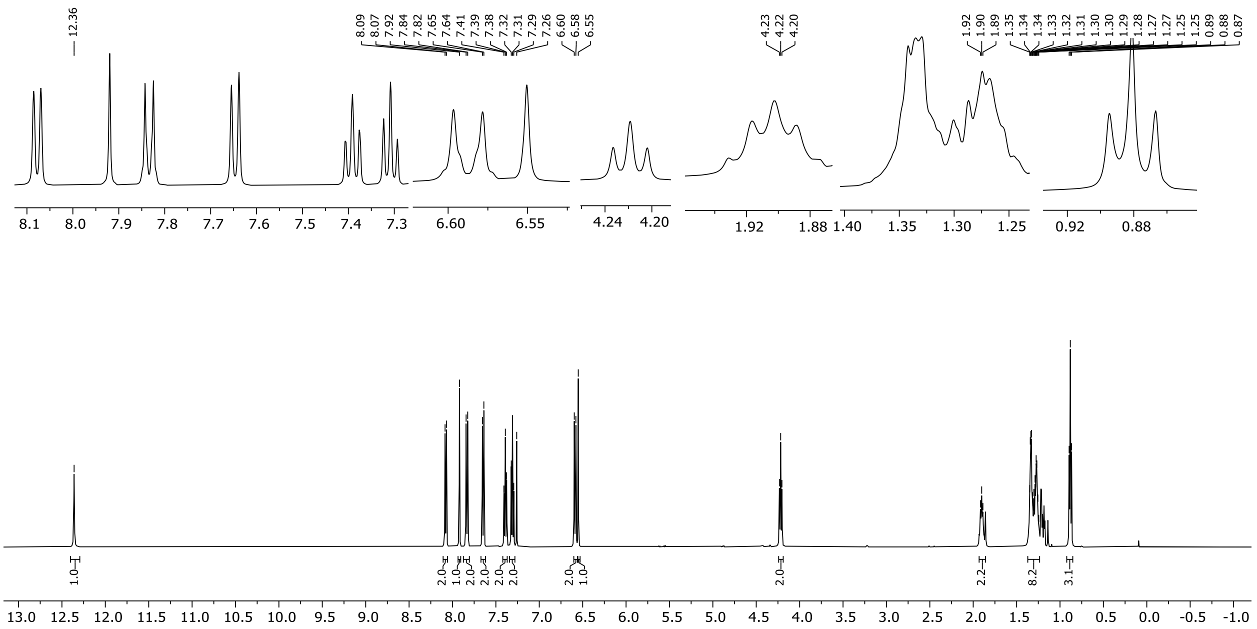

^{13}C-NMR (126 MHz, $CDCl_3$) spectrum of compound 5b:

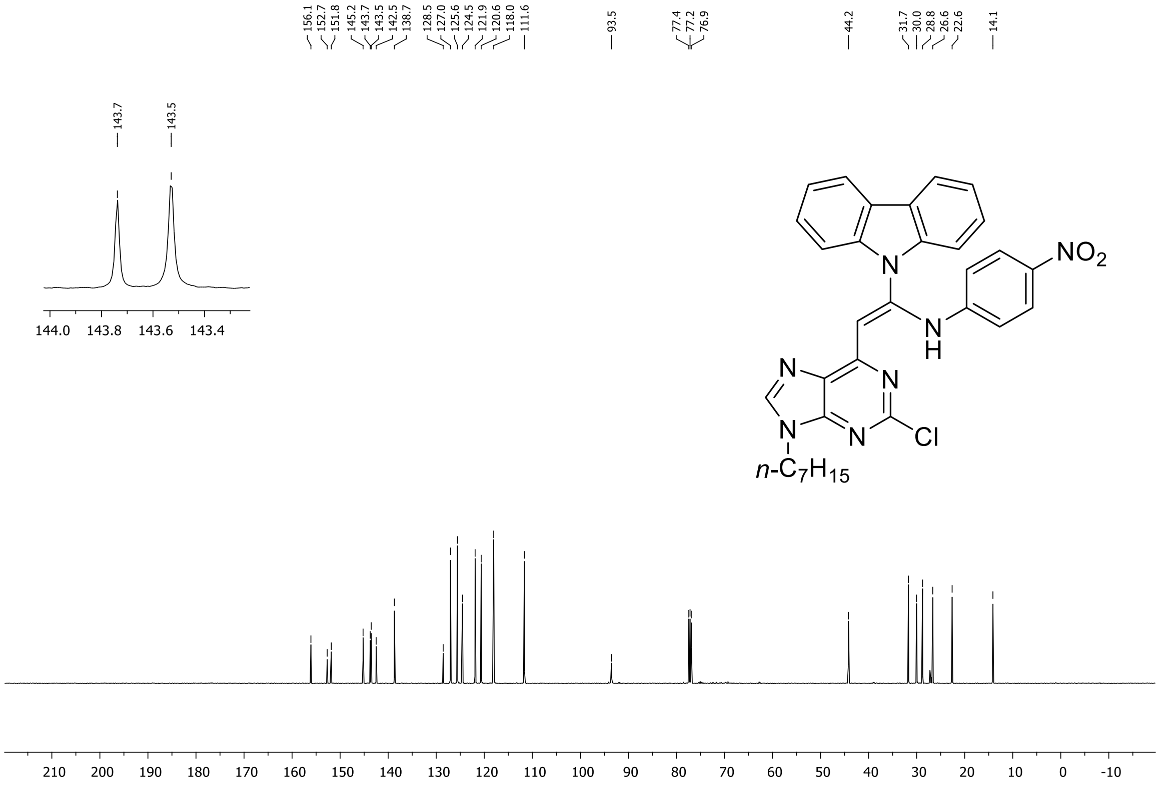

(*E*)-*N*-[1-(9*H*-Carbazol-9-yl)-2-(2-chloro-9-heptyl-9*H*-purin-6-yl)vinyl]-4-methoxyaniline (5c)

^{1}H-NMR (500 MHz, CDCl$_3$) spectrum of compound 5c:

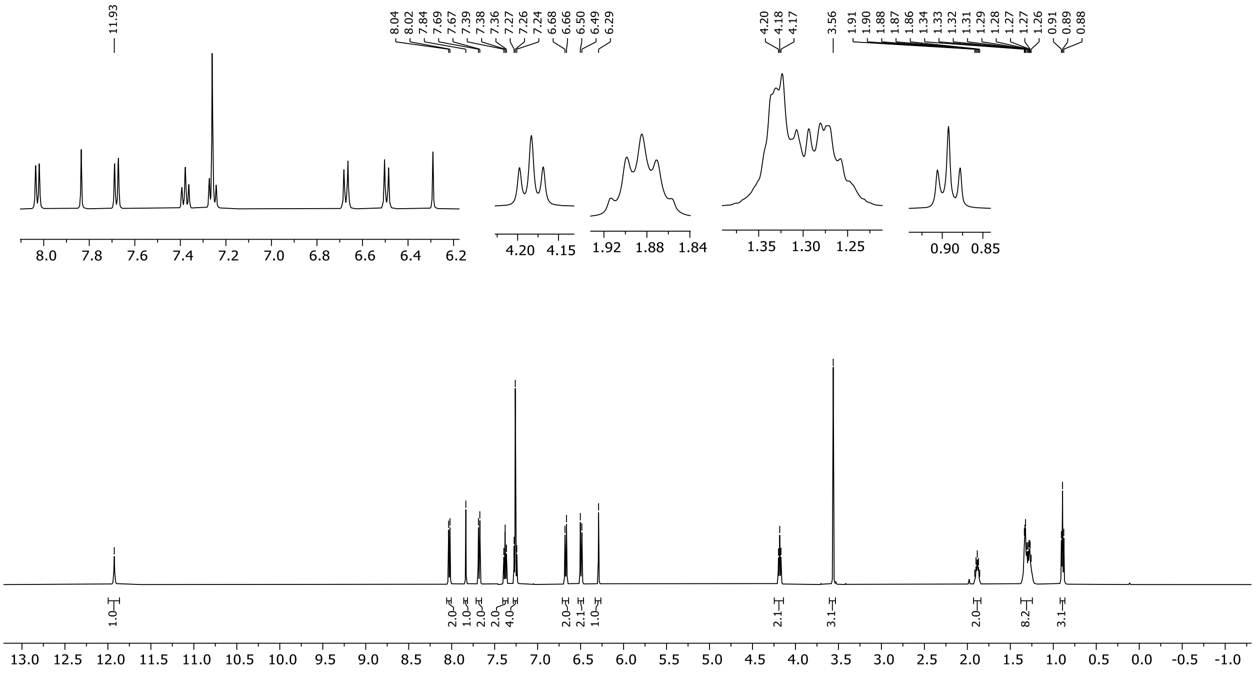

^{13}C-NMR (126 MHz, CDCl$_3$) spectrum of compound 5c:

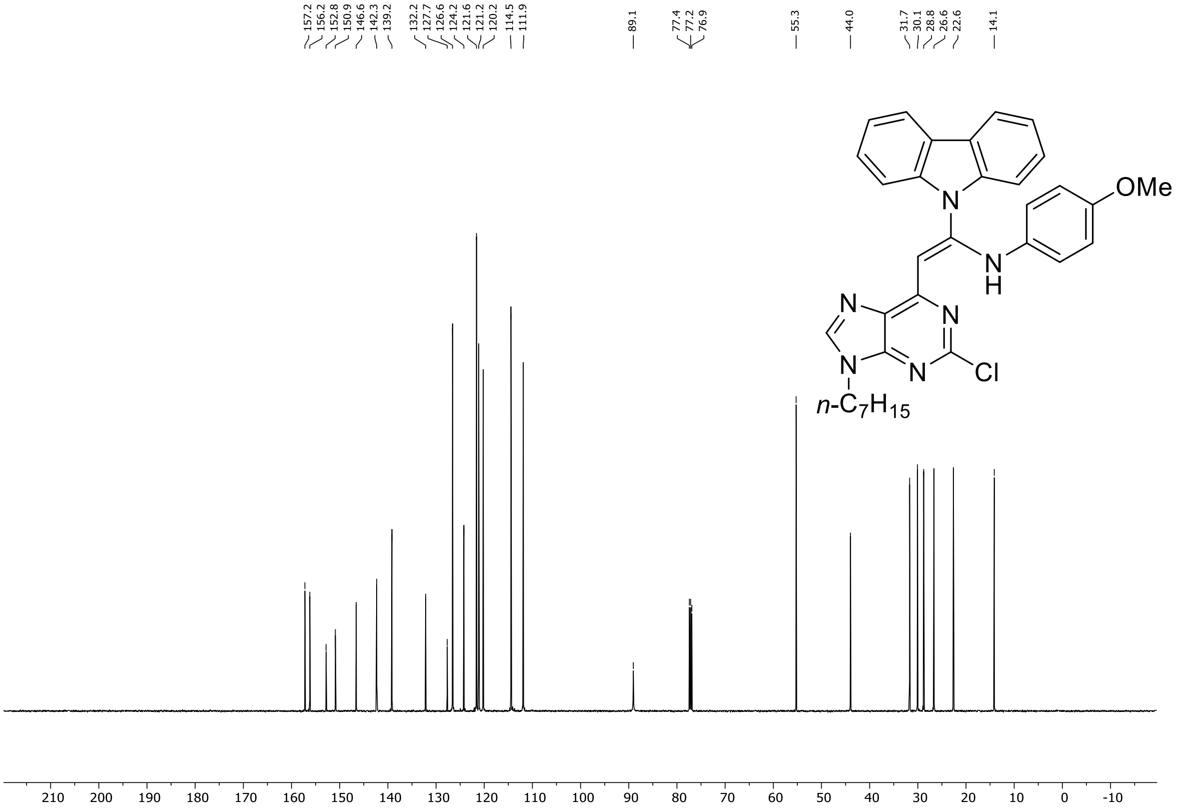

(*E*)-*N*-{[1-(9*H*-Carbazol-9-yl)-2-(2-chloro-9-heptyl-9*H*-purin-6-yl)vinyl]amino}benzonitrile (5d)

^{1}H-NMR (500 MHz, $CDCl_3$) spectrum of compound 5d:

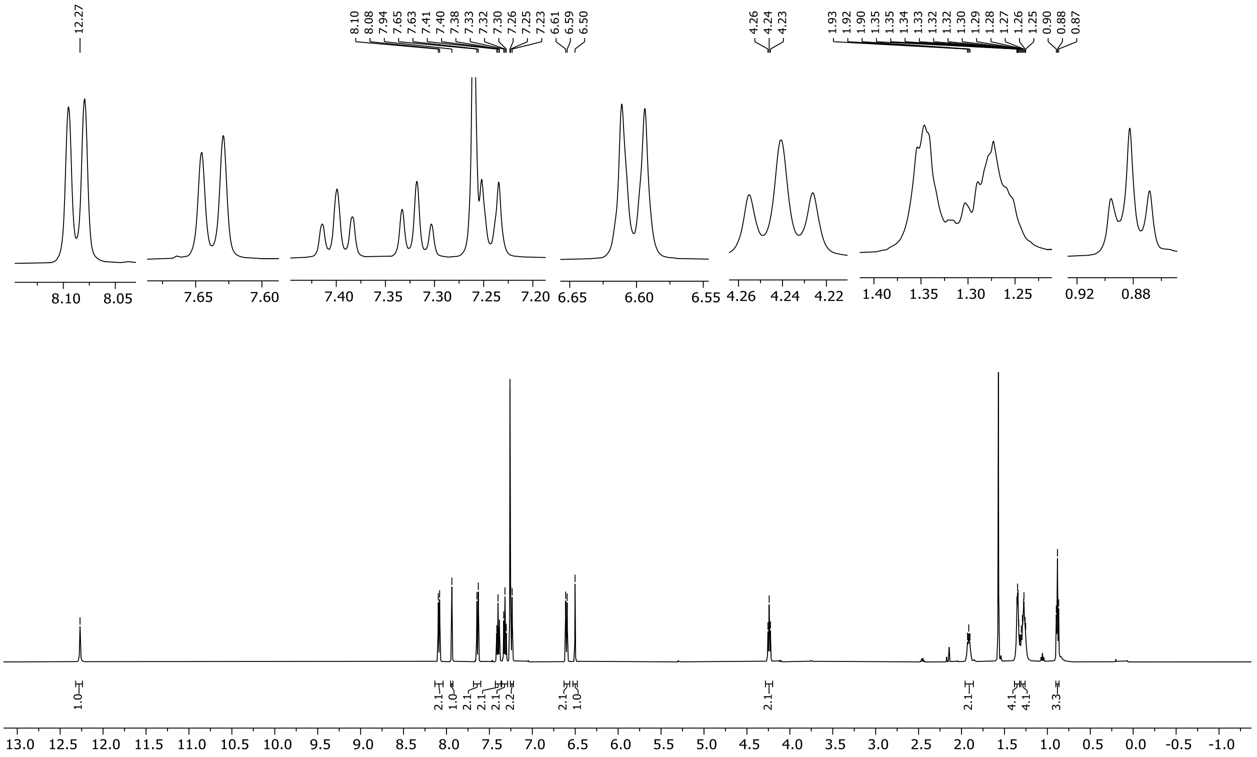

^{13}C-NMR (126 MHz, $CDCl_3$) spectrum of compound 5d:

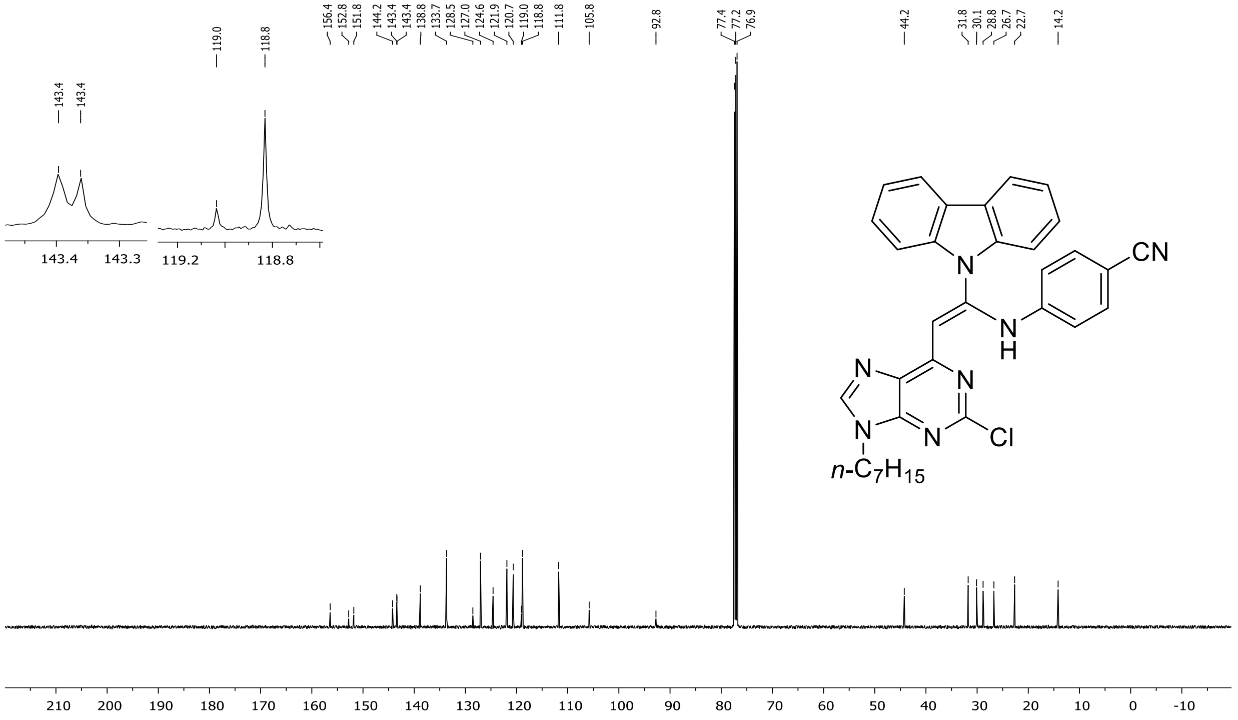

(E)-N^1-[1-(9H-carbazol-9-yl)-2-(2-chloro-9-heptyl-9H-purin-6-yl)vinyl]-N^4,N^4-dimethylbenzene-1,4-diamine (5e)

^{1}H-NMR (500 MHz, $CDCl_3$) spectrum of compound 5e:

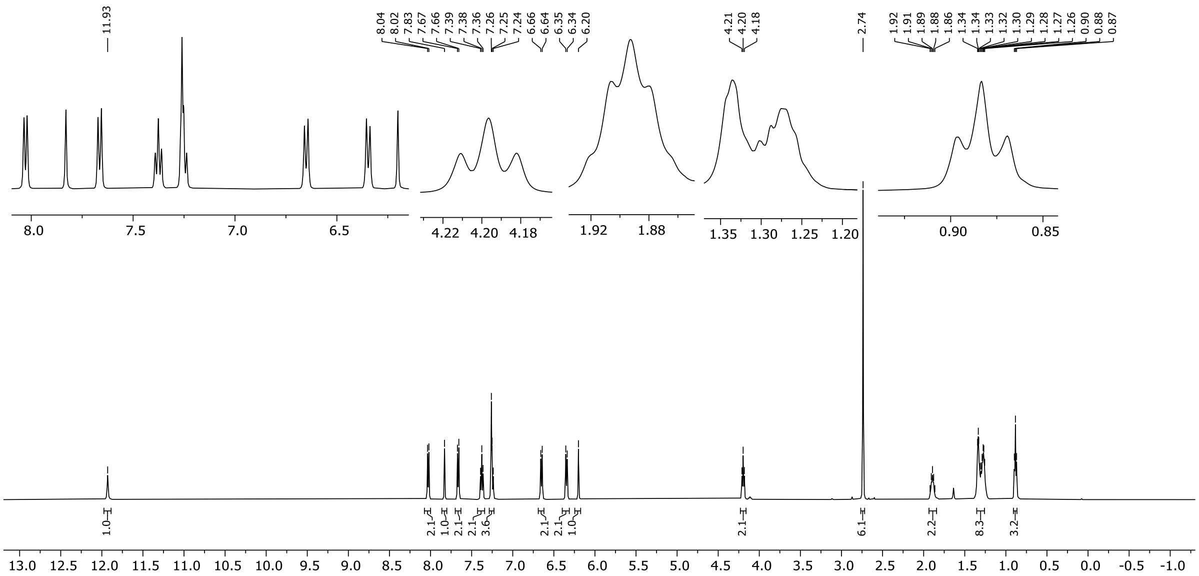

^{13}C-NMR (126 MHz, $CDCl_3$) spectrum of compound 5e:

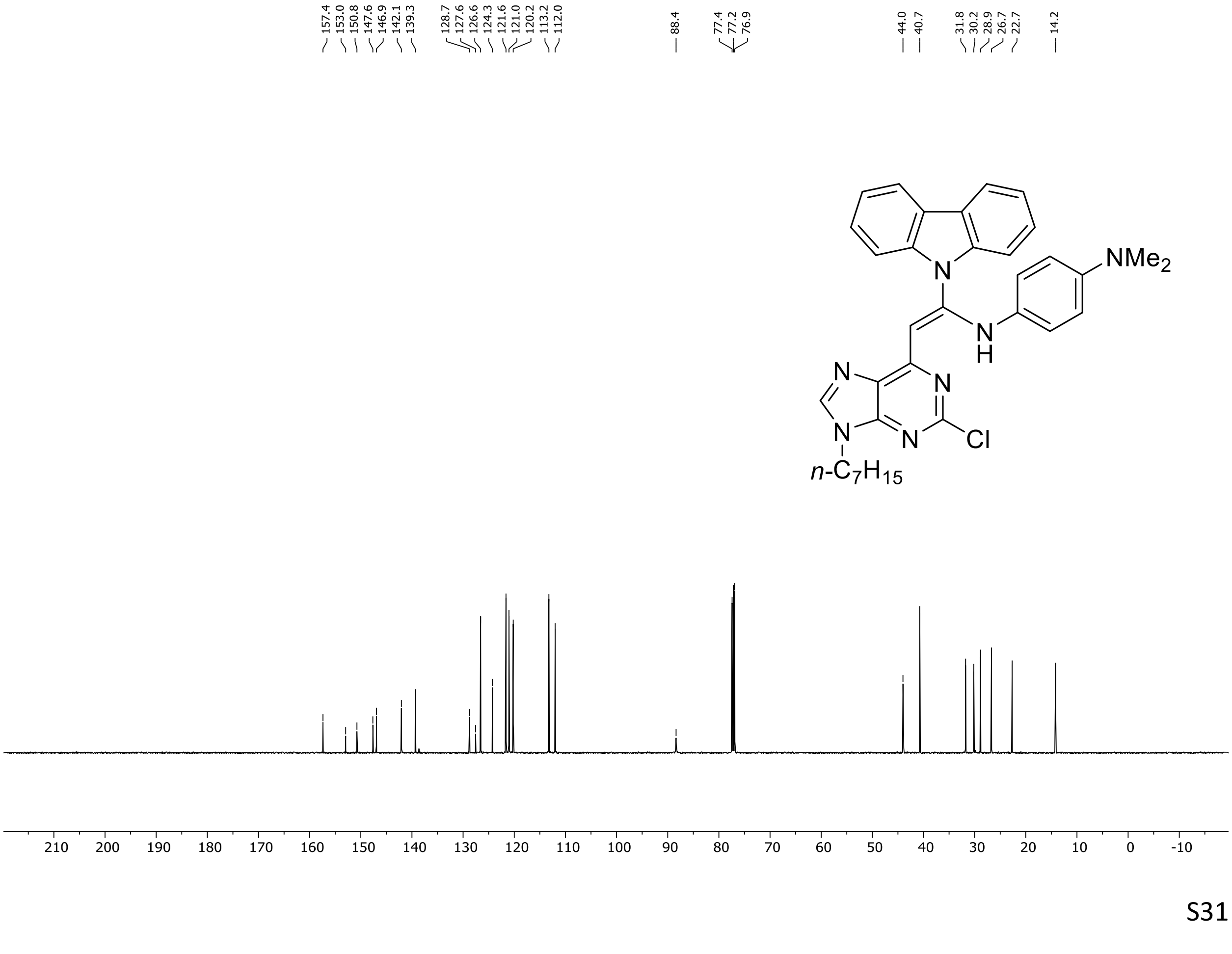

(*E*)-*N*-(1-(9*H*-Carbazol-9-yl)-2-(9-heptyl-2-(1-phenyl-1*H*-1,2,3-triazol-4-yl)-9*H*-purin-6-yl)vinyl)aniline (5h)

^{1}H-NMR (500 MHz, $CDCl_3$) spectrum of compound 5h:

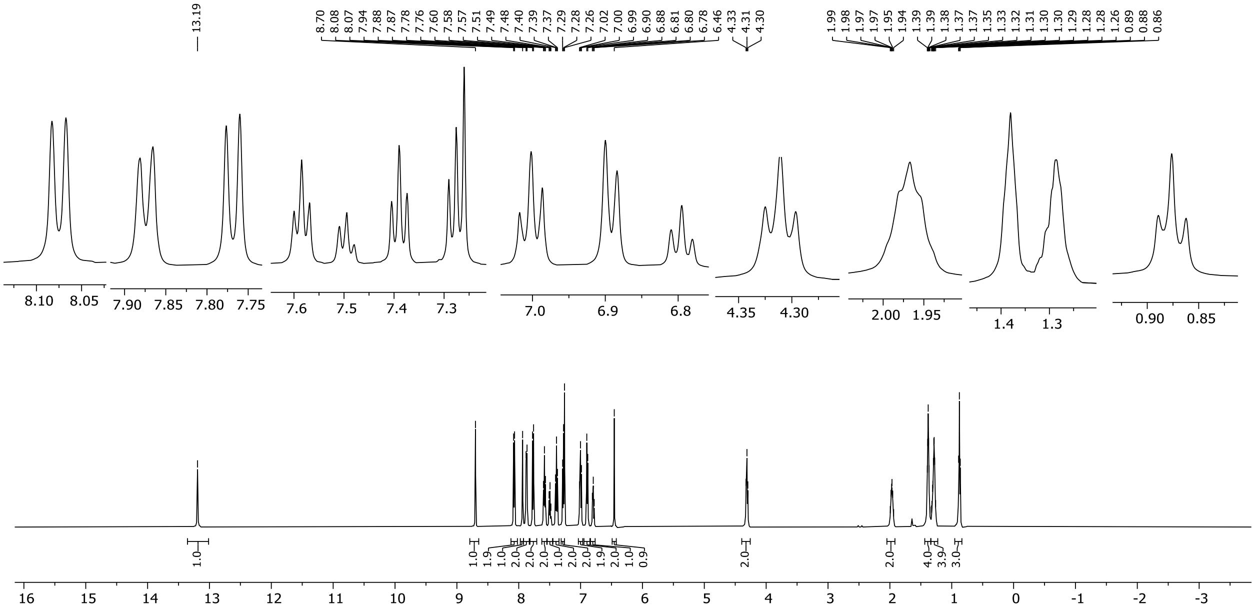

^{13}C-NMR (126 MHz, $CDCl_3$) spectrum of compound 5h:

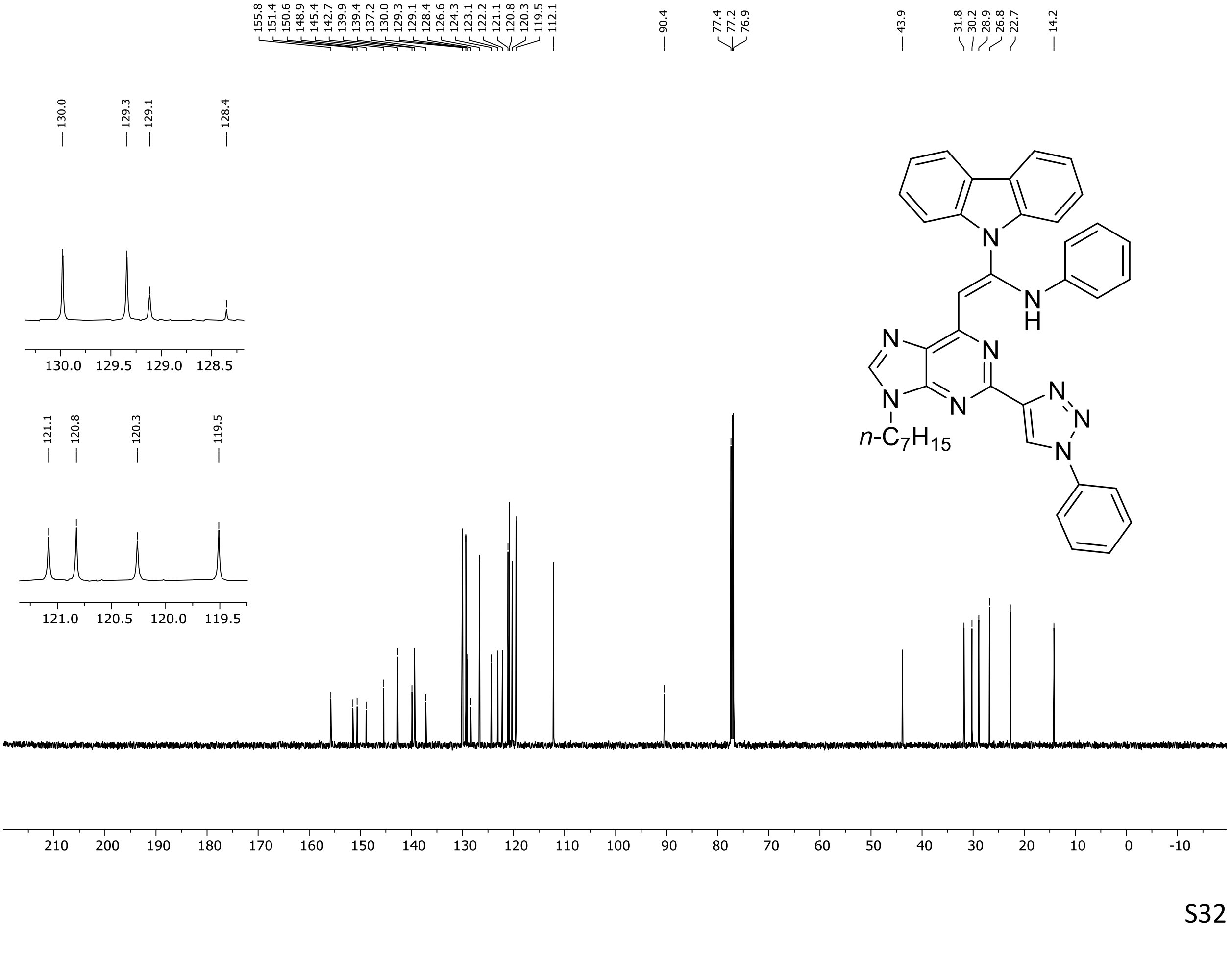

9-[3-(2-Chloro-9-heptyl-9*H*-purin-6-yl)-1*H*-indol-2-yl]-9*H*-carbazole (6a)

^{1}H-NMR (500 MHz, $CDCl_3$) spectrum of compound 6a:

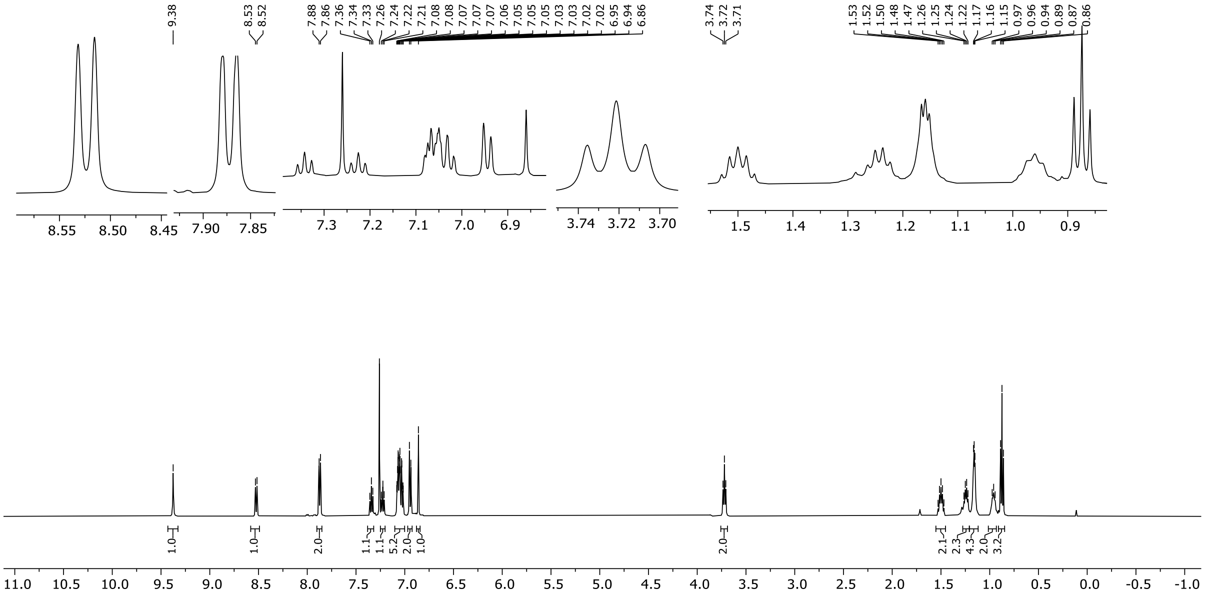

^{13}C-NMR (126 MHz, $CDCl_3$) spectrum of compound 6a:

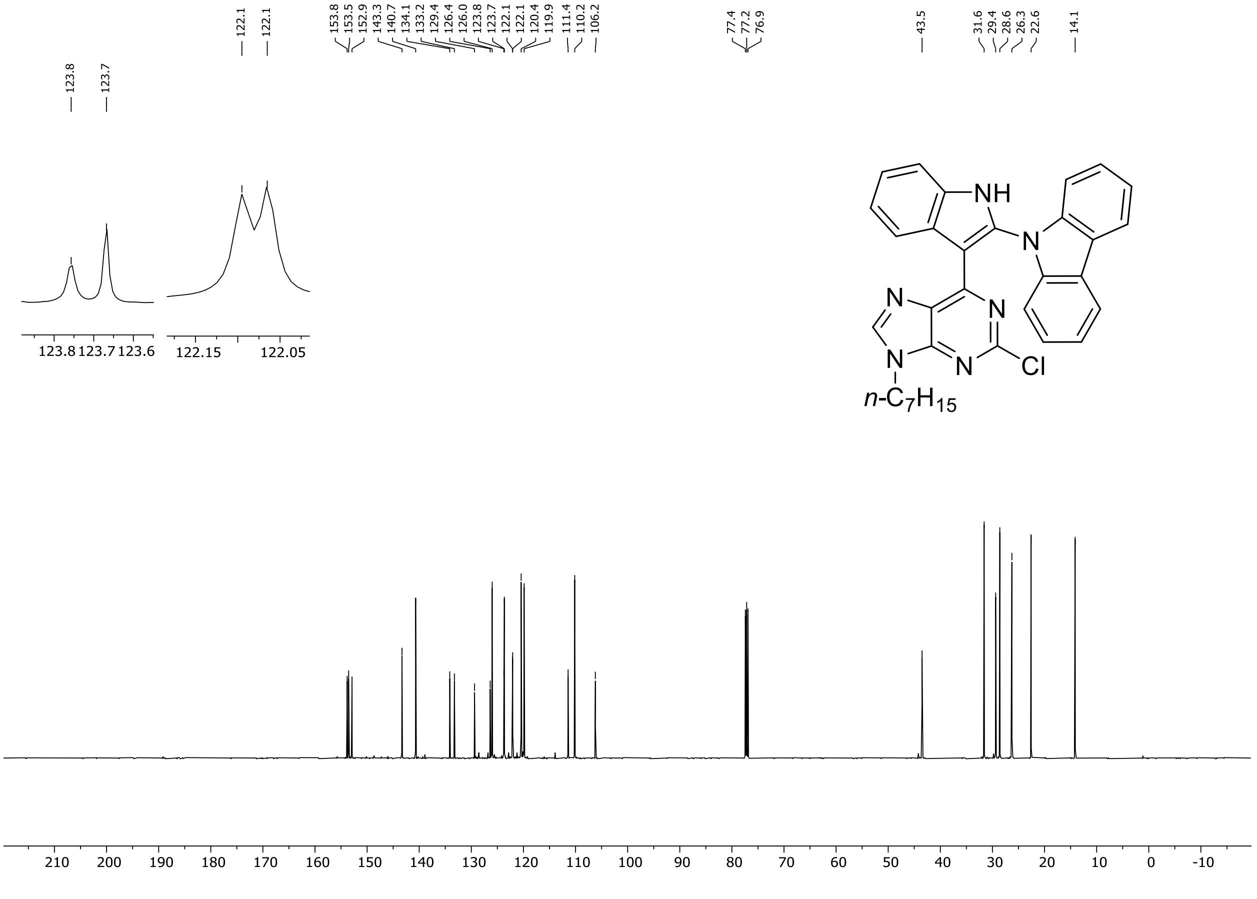

9-[3-(2-Chloro-9-heptyl-9*H*-purin-6-yl)-5-nitro-1*H*-indol-2-yl]-9*H*-carbazole (6b)

^{1}H-NMR (500 MHz, $CDCl_3$) spectrum of compound 6b:

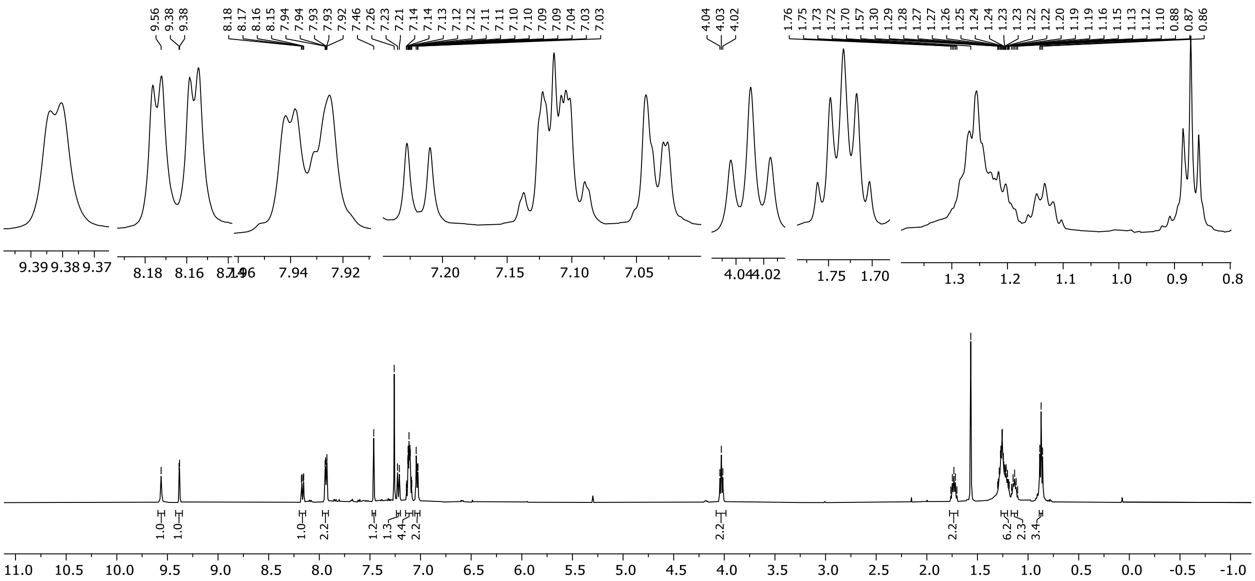

^{13}C-NMR (126 MHz, $CDCl_3$) spectrum of compound 6b:

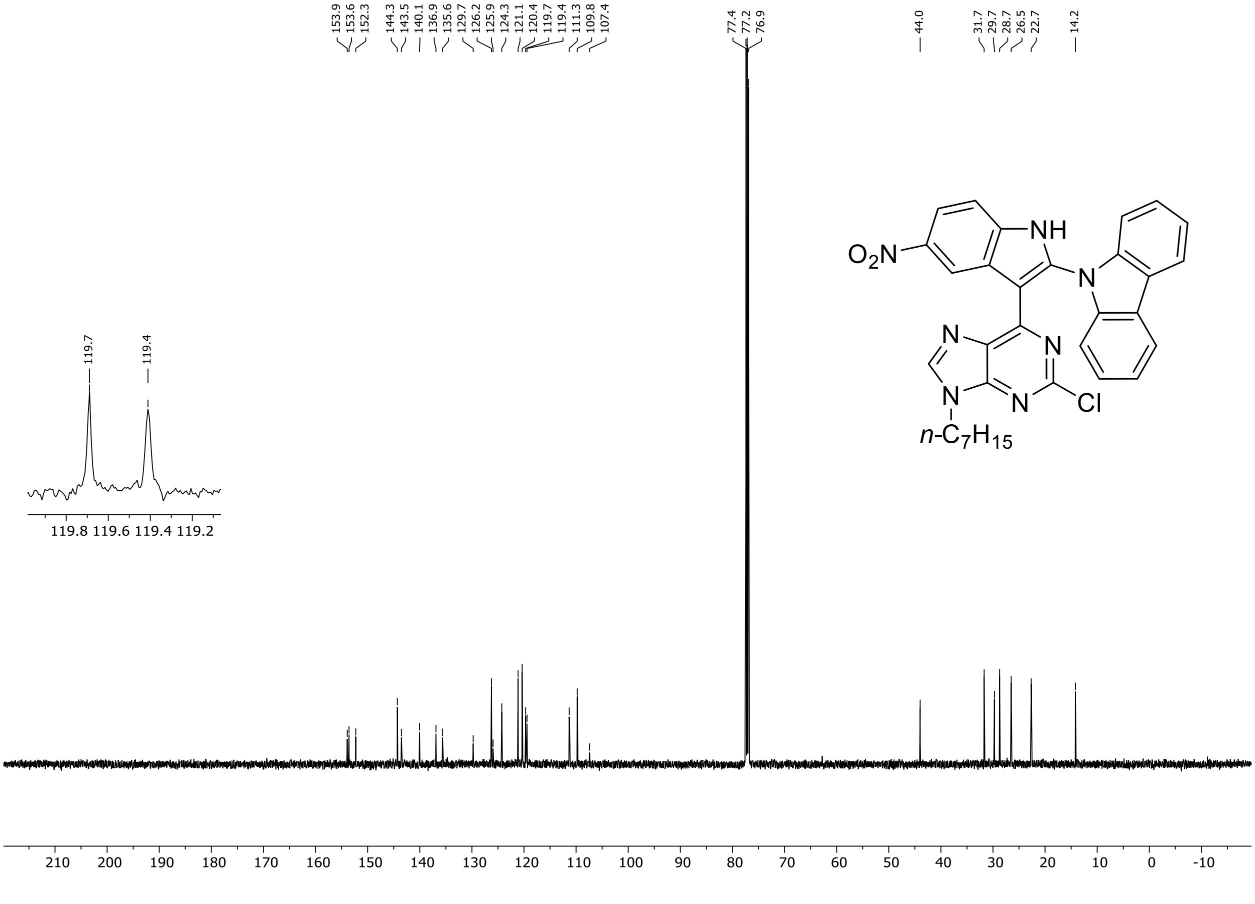

9-[3-(2-Chloro-9-heptyl-9*H*-purin-6-yl)-5-methoxy-1*H*-indol-2-yl]-9*H*-carbazole (6c)

^{1}H-NMR (500 MHz, $CDCl_3$) spectrum of compound 6c:

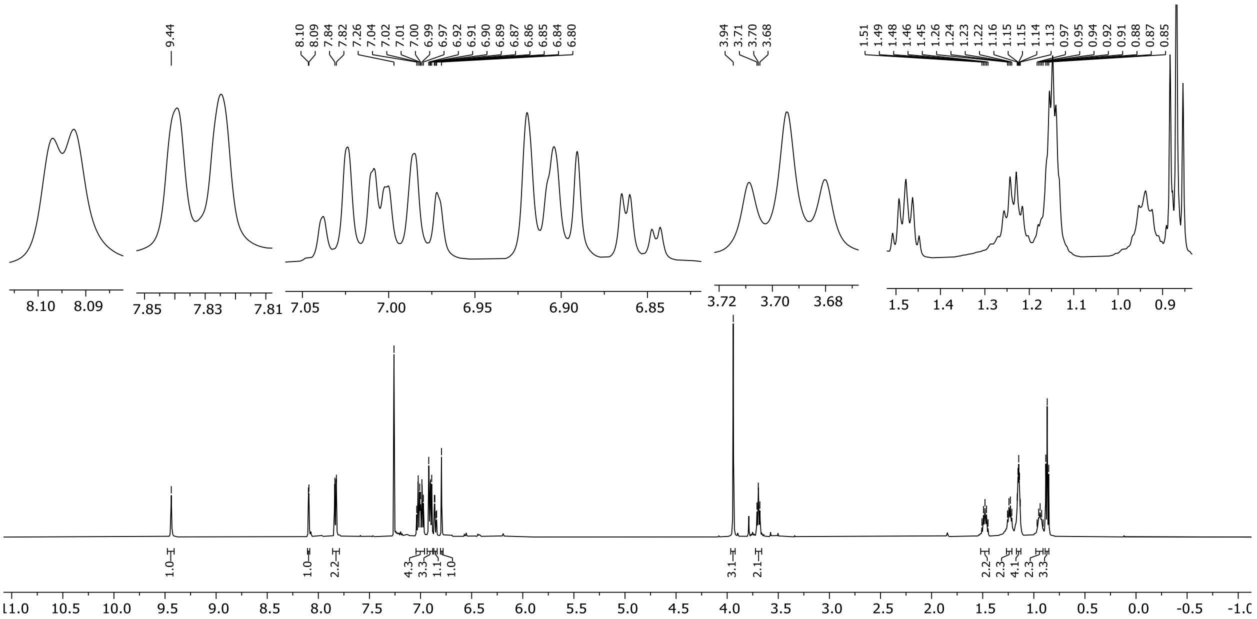

^{13}C-NMR (126 MHz, $CDCl_3$) spectrum of compound 6c:

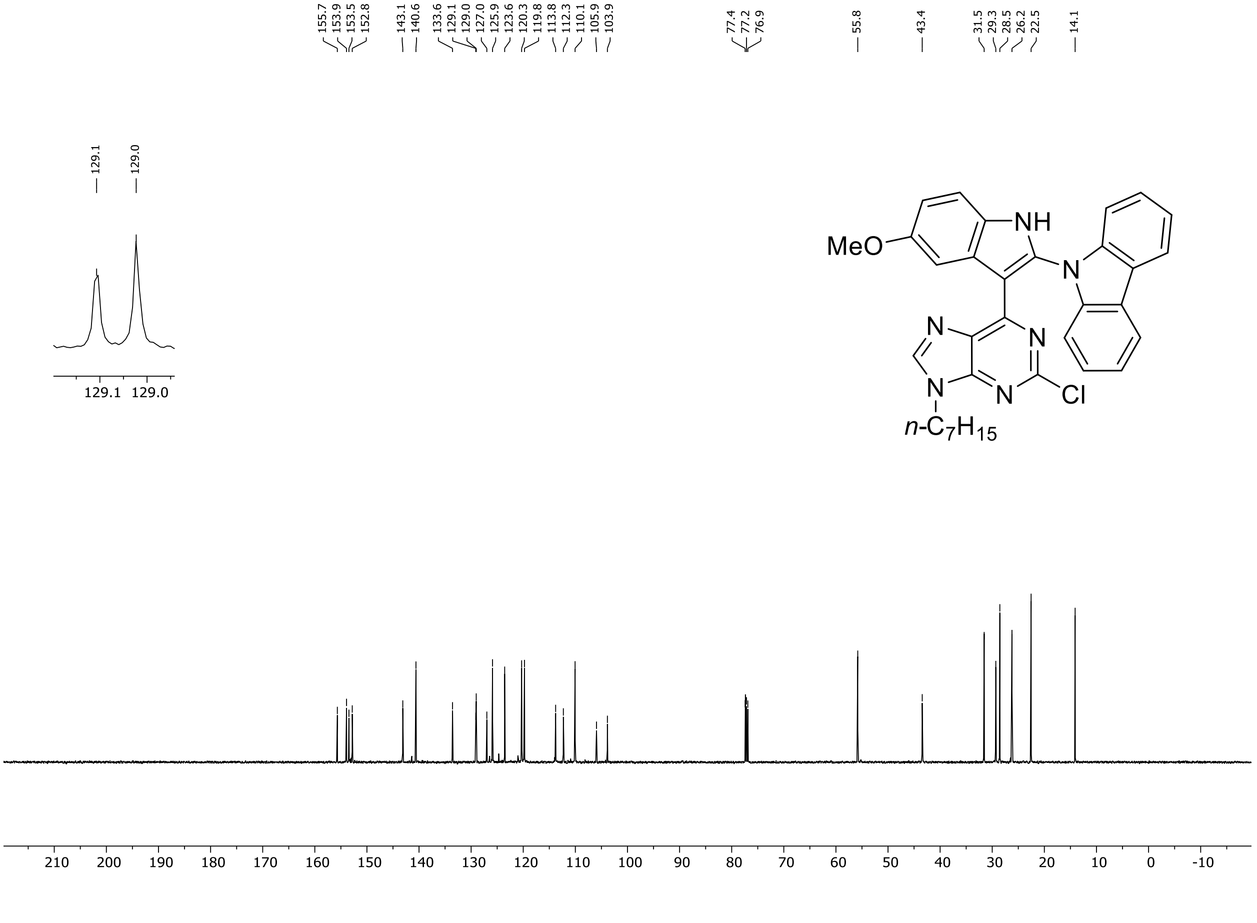

2-(9*H*-Carbazol-9-yl)-3-(2-chloro-9-heptyl-9*H*-purin-6-yl)-1*H*-indole-5-carbonitrile (6d)

^{1}H-NMR (500 MHz, DMSO-d$_6$) spectrum of compound 6d:

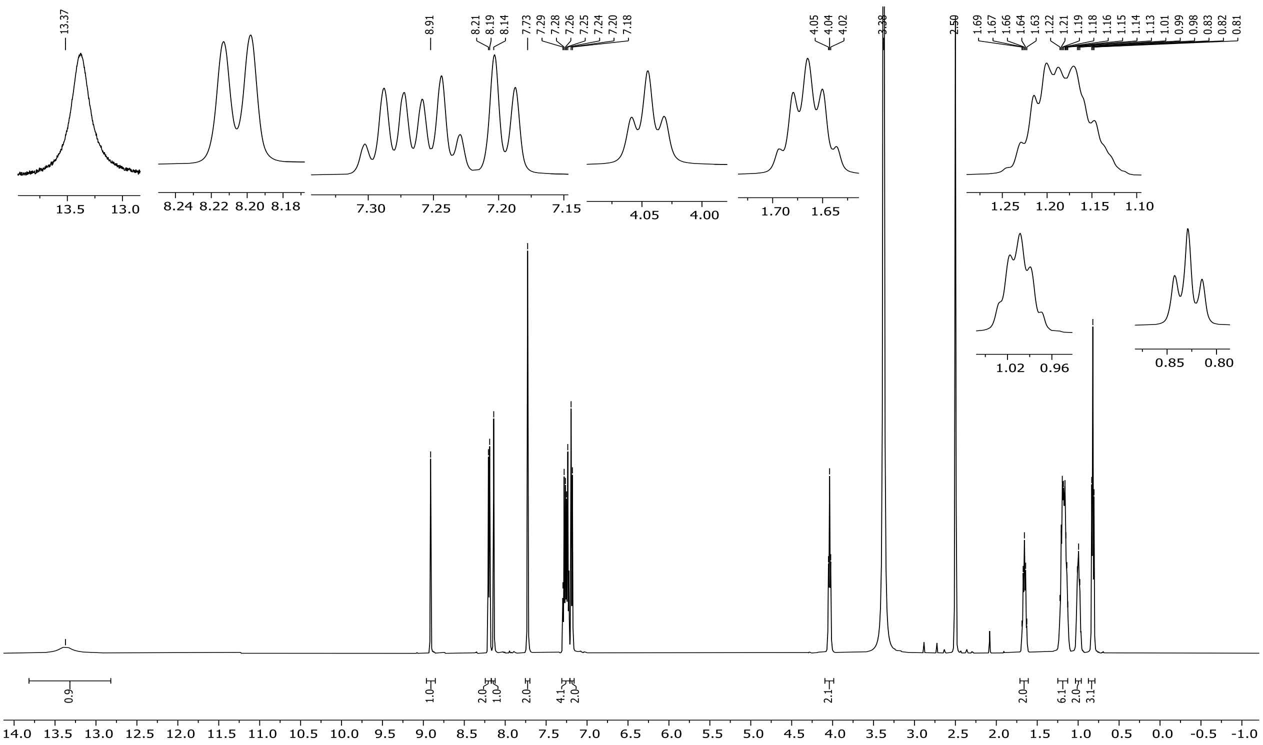

^{13}C-NMR (126 MHz, DMSO-d$_6$) spectrum of compound 6d:

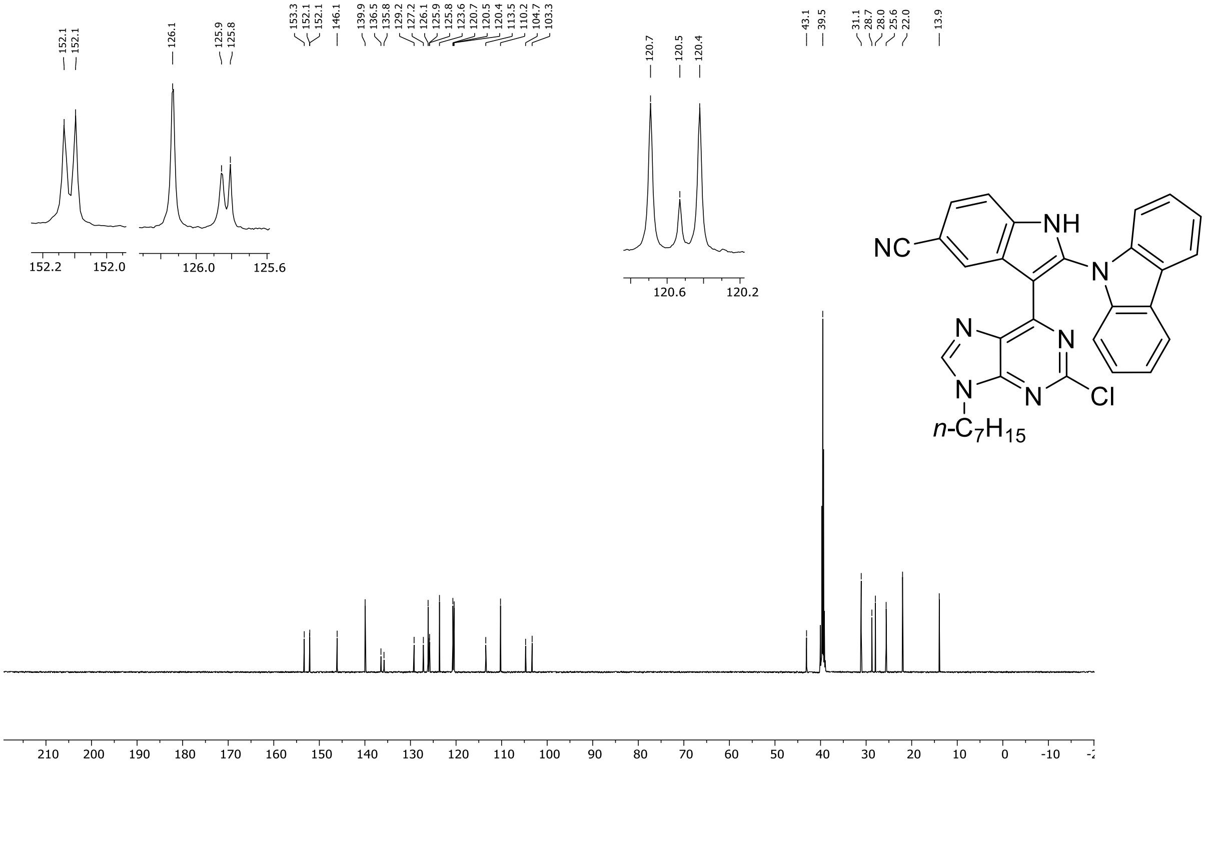

9-[3-(9-Heptyl-9*H*-purin-6-yl)-1*H*-indol-2-yl]-9*H*-carbazole (6f)

^{1}H-NMR (500 MHz, $CDCl_3$) spectrum of compound 6f:

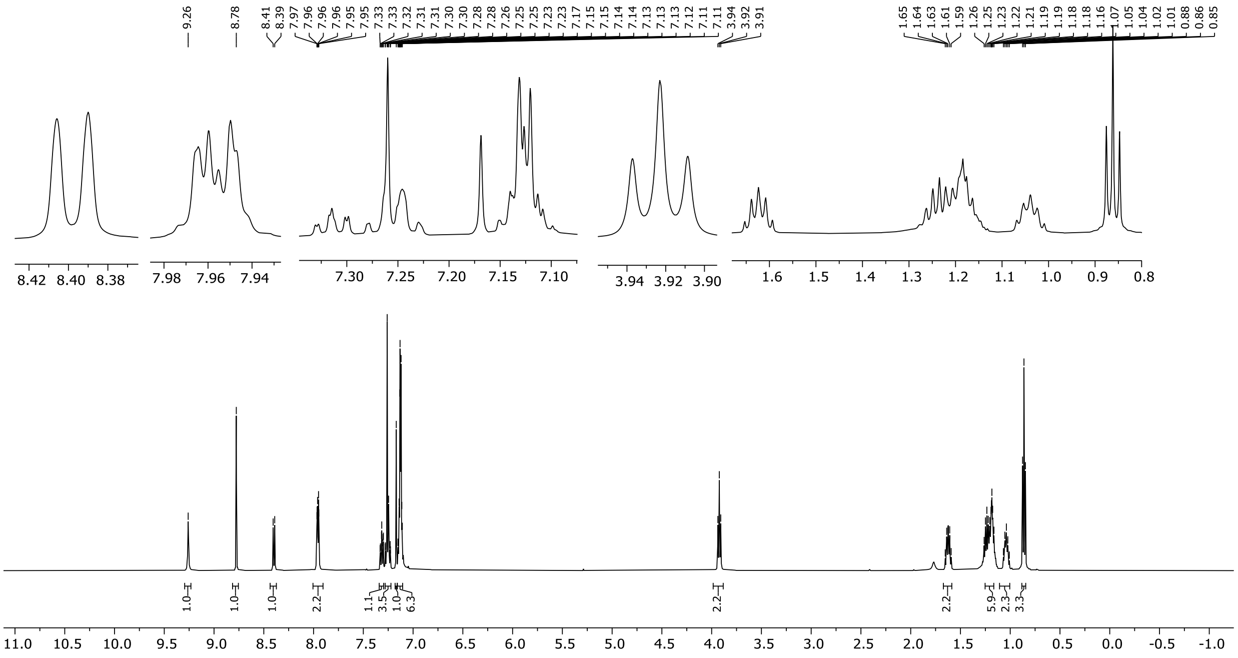

^{13}C-NMR (126 MHz, $CDCl_3$) spectrum of compound 6f:

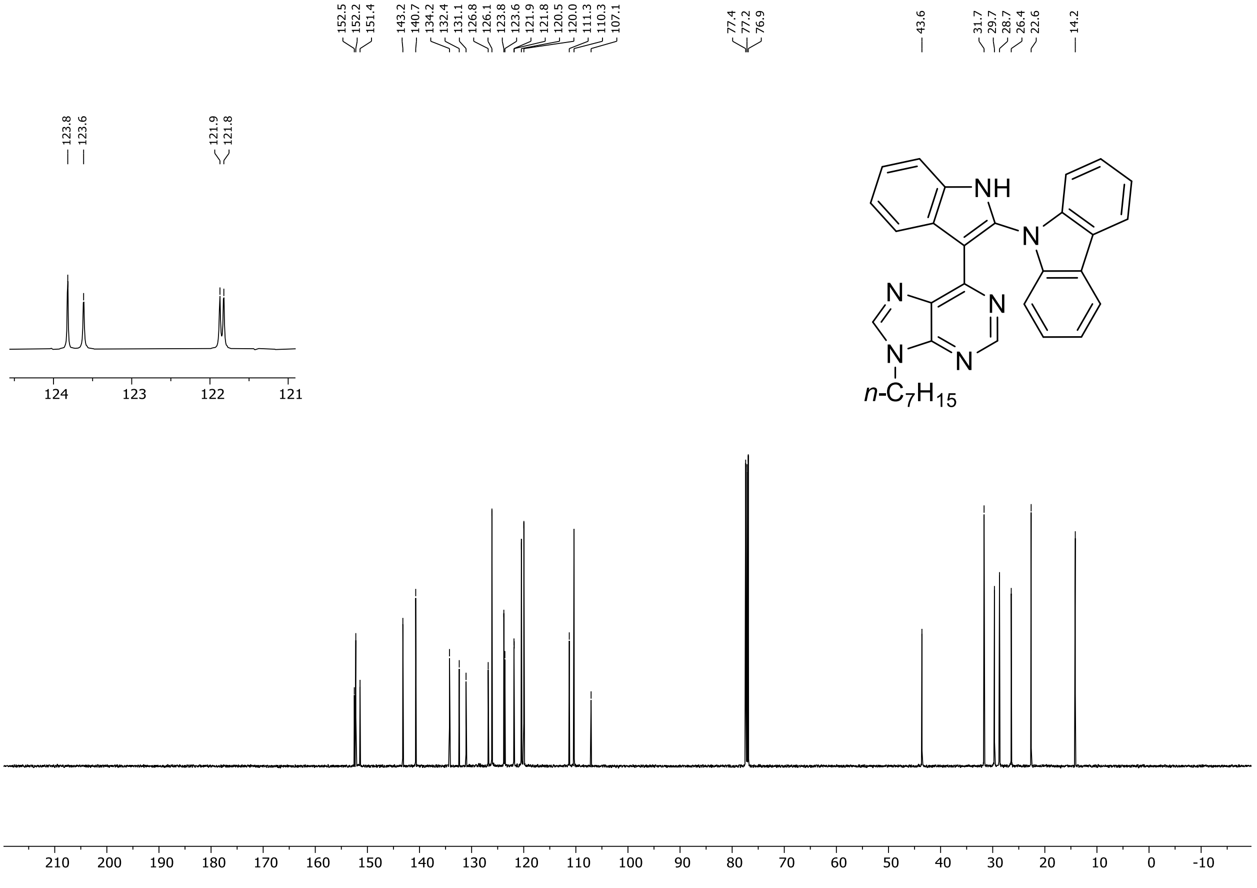

9-[3-(9-Heptyl-9*H*-purin-6-yl)-5-nitro-1*H*-indol-2-yl]-9*H*-carbazole (6g)

^{1}H-NMR (500 MHz, DMSO-d$_6$) spectrum of compound 6g:

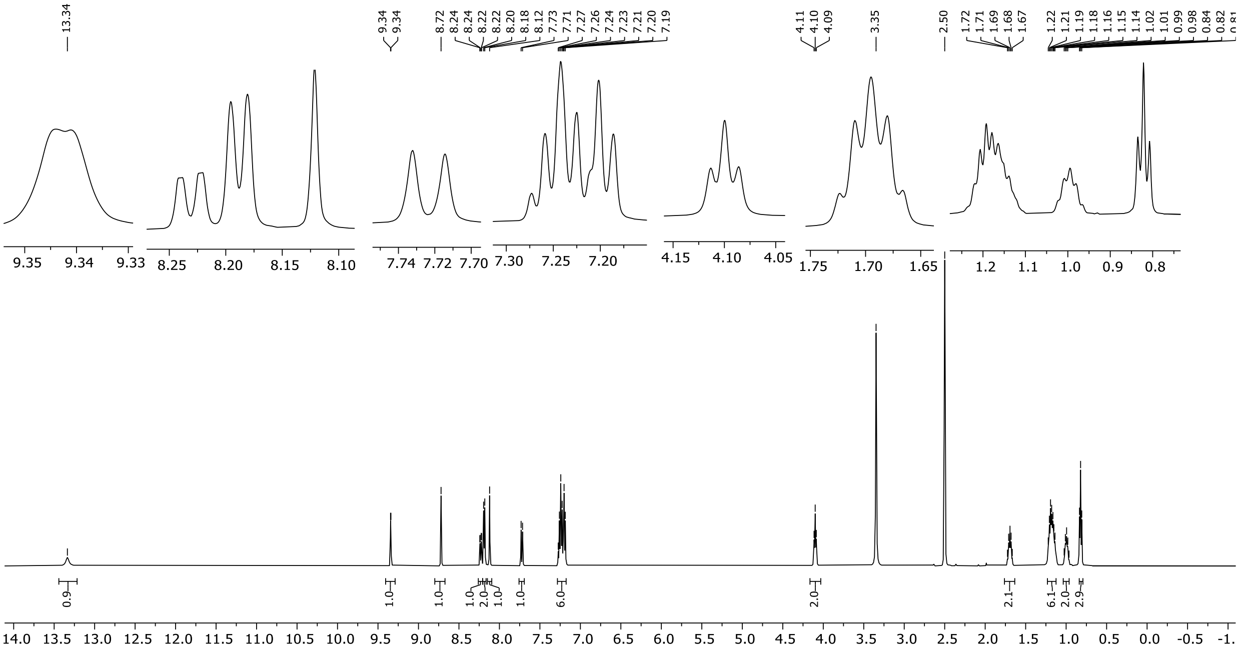

^{13}C-NMR (126 MHz, DMSO-d$_6$) spectrum of compound 6g:

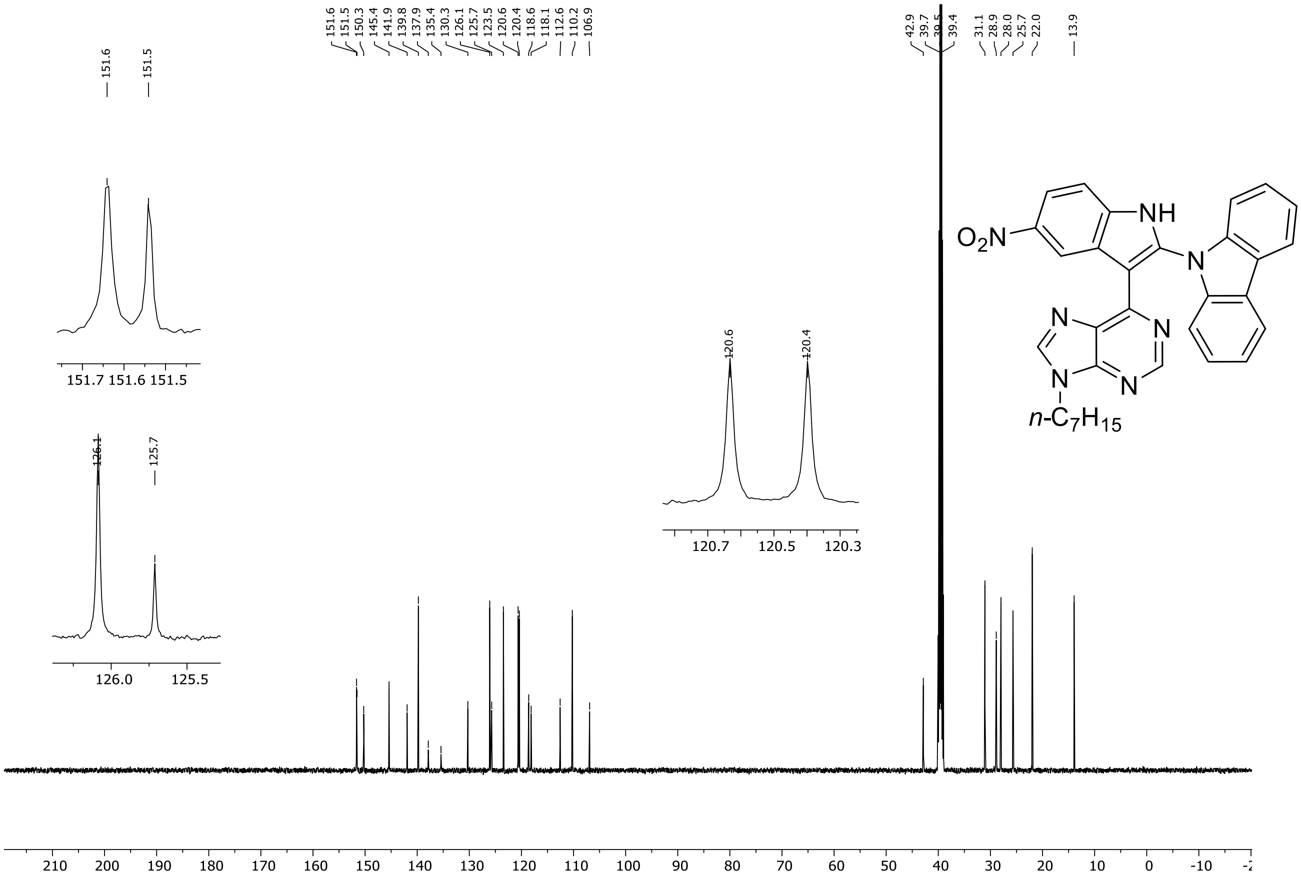

(*E*)-2-(2-Chloro-9-heptyl-9*H*-purin-6-yl)-*N*,*N*,*N*'-triphenylethene-1,1-diamine (7a)

^{1}H-NMR (500 MHz, $CDCl_3$) spectrum of compound 7a:

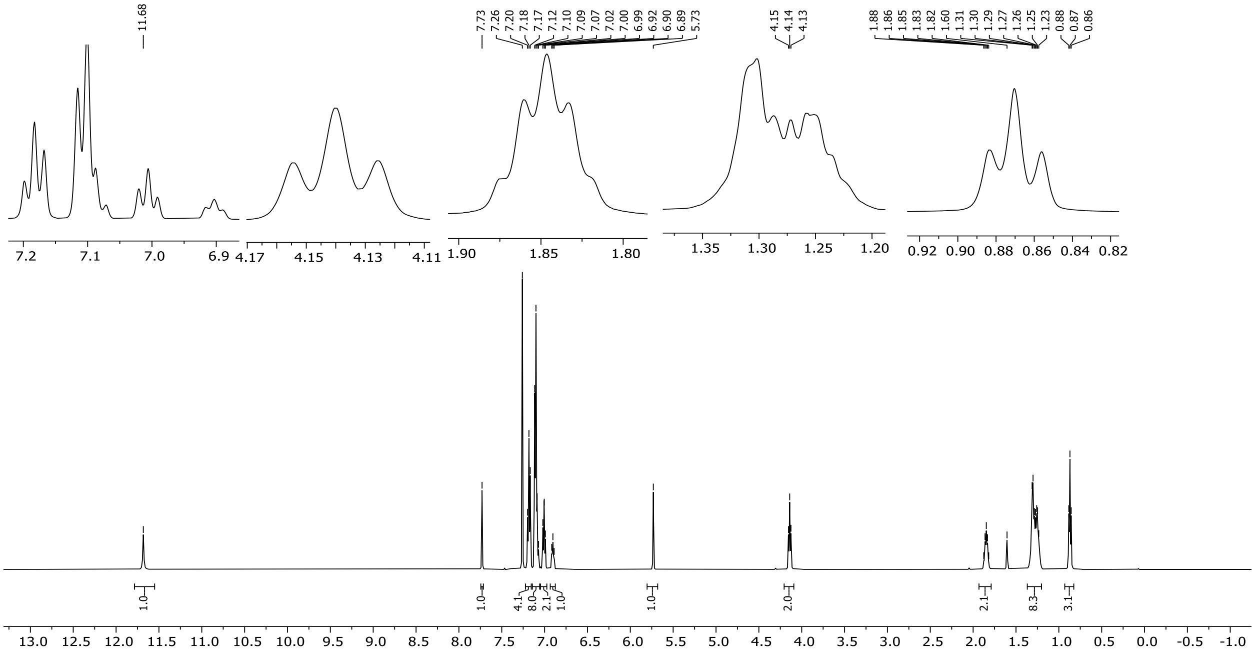

^{13}C-NMR (126 MHz, $CDCl_3$) spectrum of compound 7a:

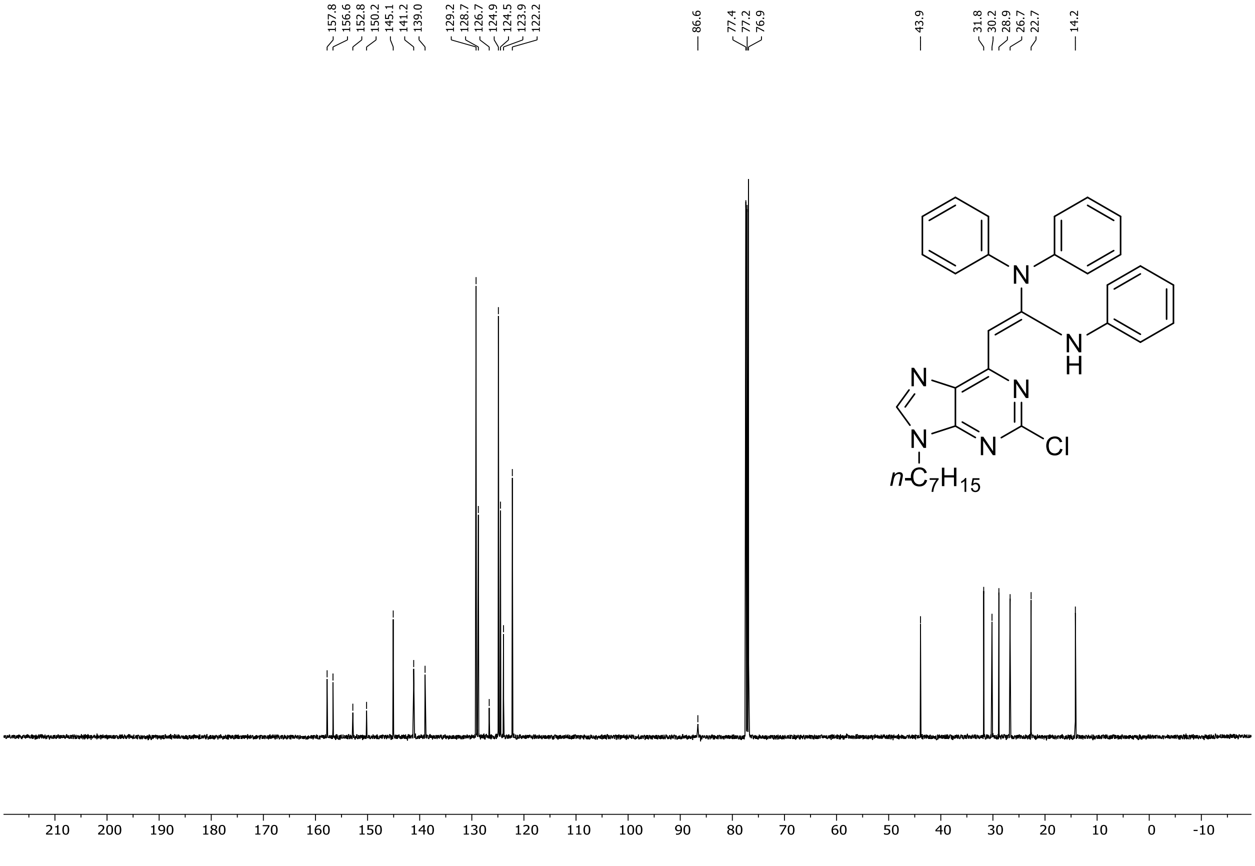

(*E*)-2-(2-Chloro-9-heptyl-9*H*-purin-6-yl)-*N*-(4-nitrophenyl)-*N*',*N*'-diphenylethene-1,1-diamine (7b)

^{1}H-NMR (500 MHz, $CDCl_3$) spectrum of compound 7b:

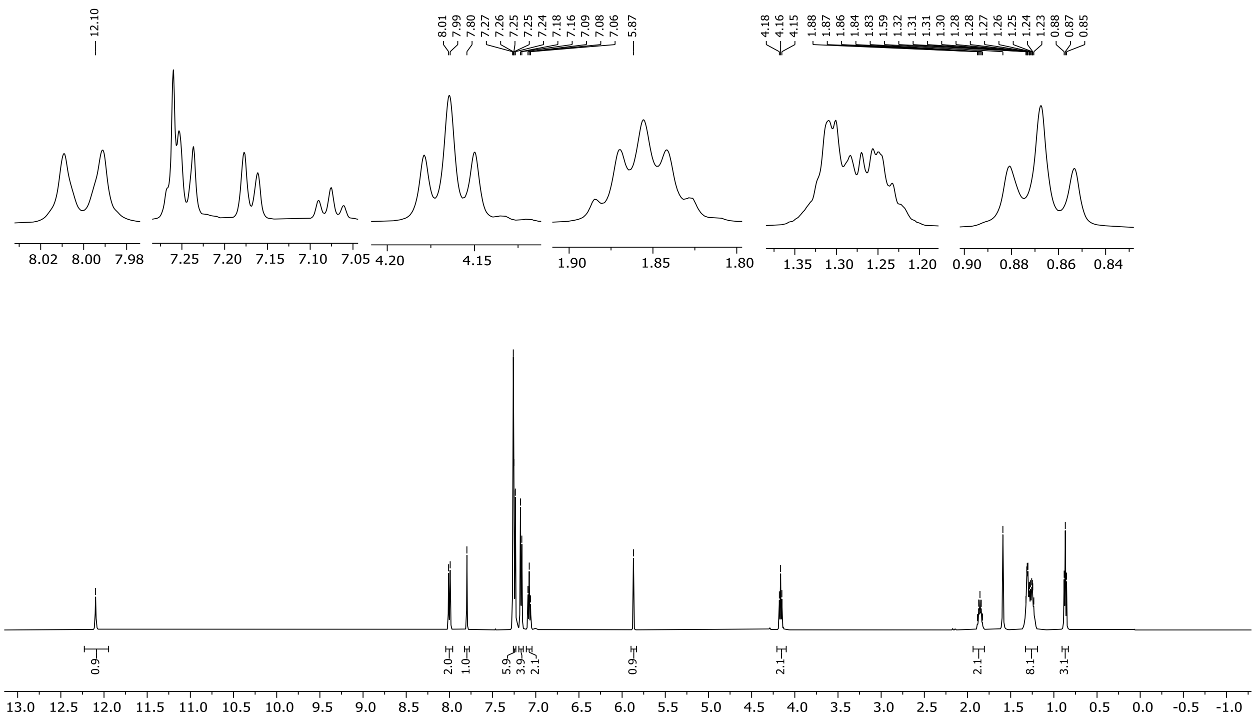

^{13}C-NMR (126 MHz, $CDCl_3$) spectrum of compound 7b:

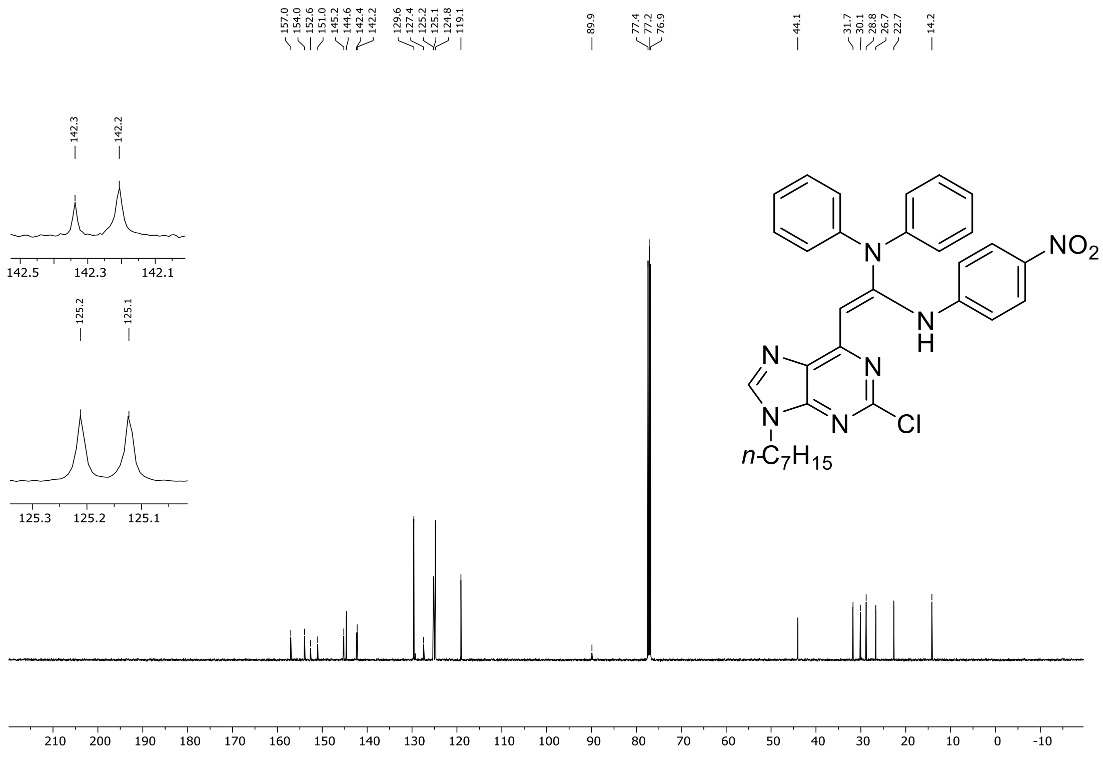

3-(2-Chloro-9-heptyl-9*H*-purin-6-yl)-*N*,1-diphenyl-1*H*-indol-2-amine (8a)

^{1}H-NMR (500 MHz, $CDCl_3$) spectrum of compound 8a:

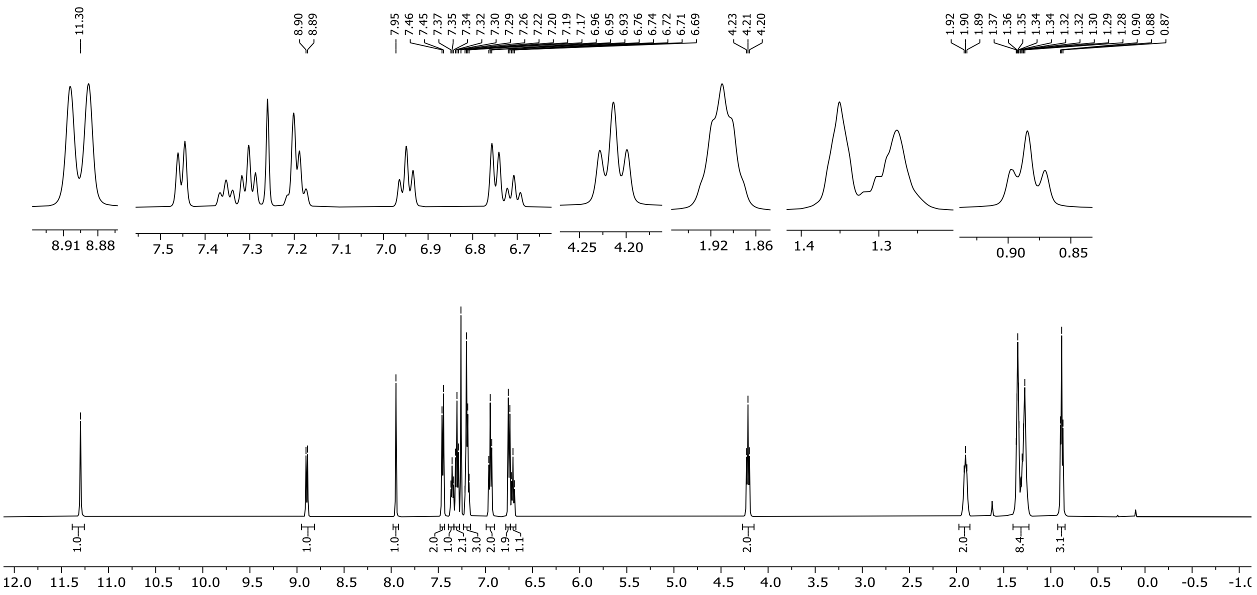

^{13}C-NMR (126 MHz, $CDCl_3$) spectrum of compound 8a:

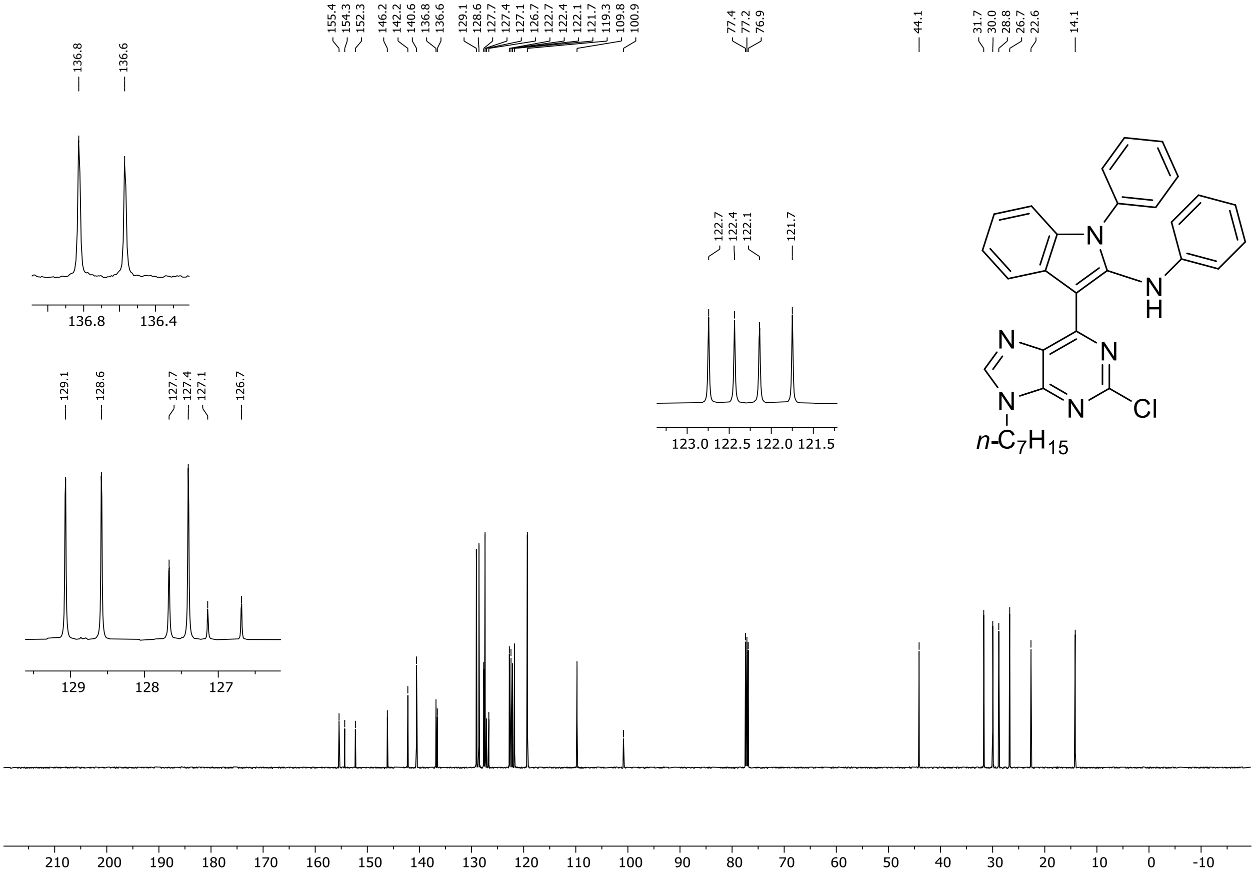

3-(2-Chloro-9-heptyl-9*H*-purin-6-yl)-*N*-(4-nitrophenyl)-1-phenyl-1*H*-indol-2-amine (8b)

^{1}H-NMR (500 MHz, $CDCl_3$) spectrum of compound 8b:

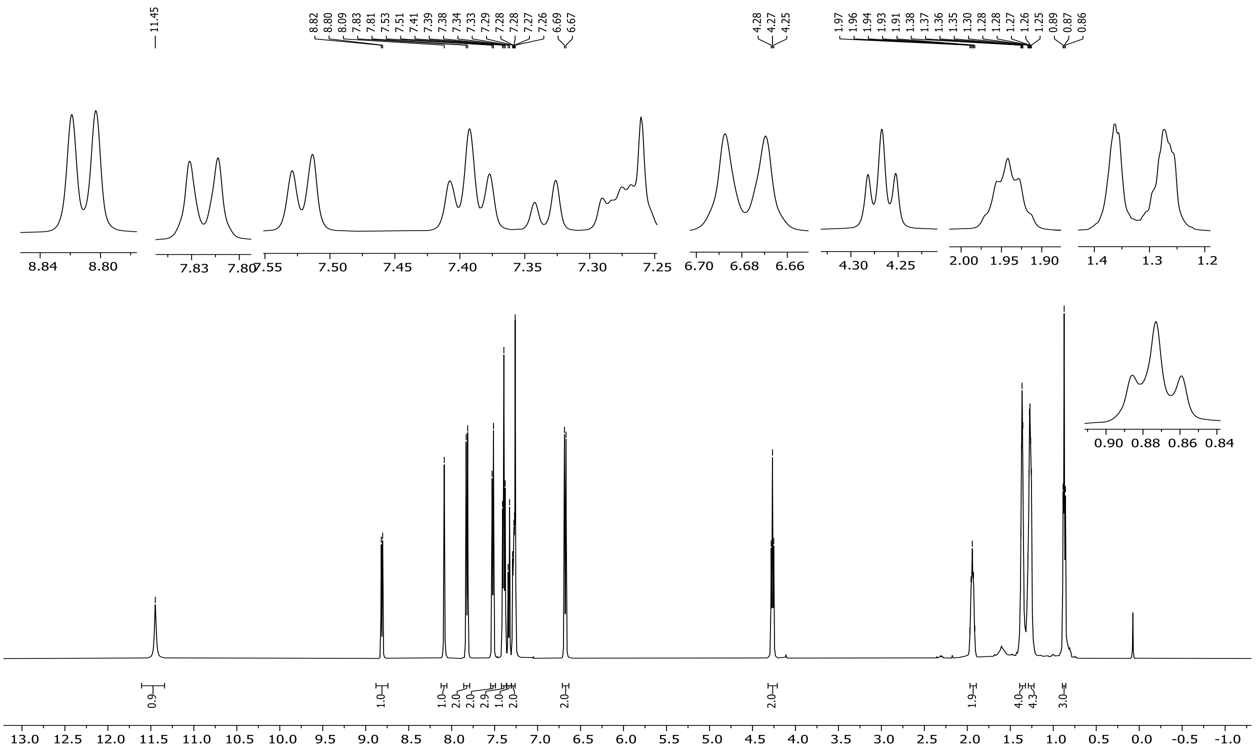

^{13}C-NMR (126 MHz, $CDCl_3$) spectrum of compound 8b:

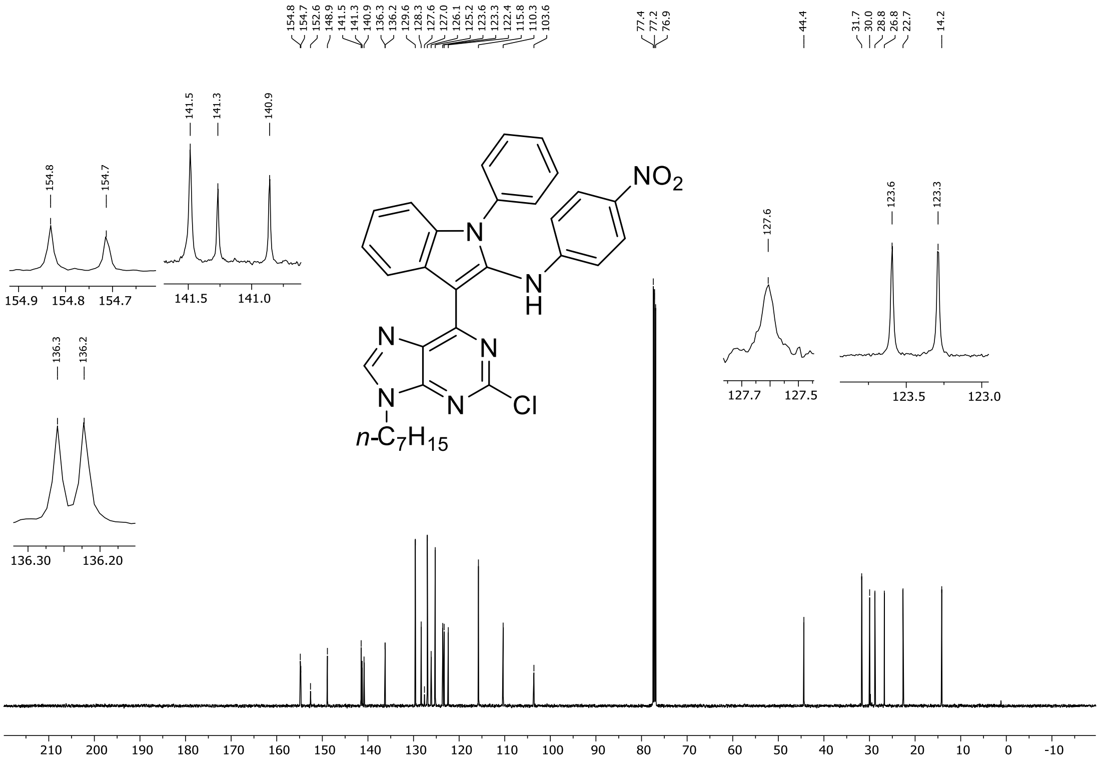

8. X-ray crystallography data

(*E*)-2-(9*H*-Carbazol-9-yl)-2-((4-nitrophenyl)imino)-1-(pyridin-2-yl)ethan-1-one (2c')

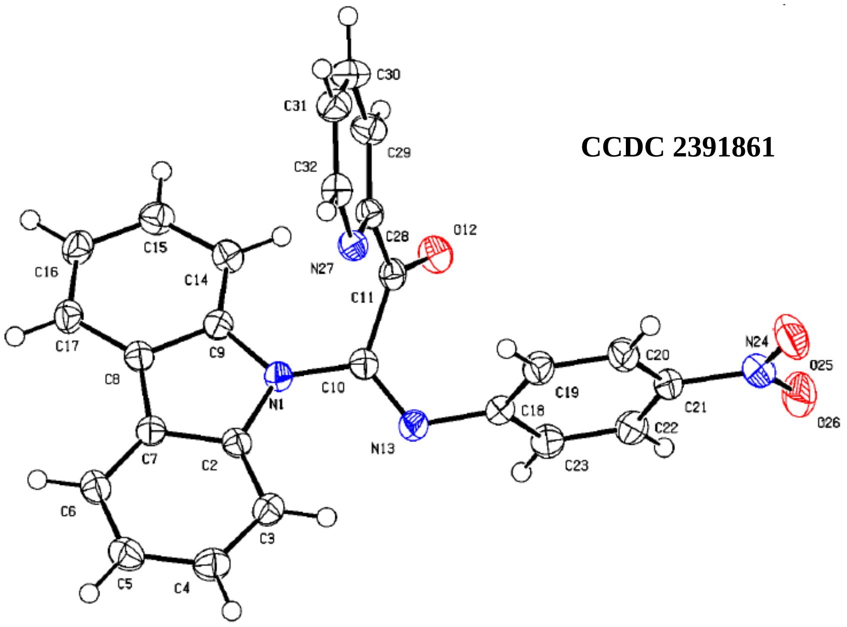

Figure S1. ORTEP plot for **2c'** with the atom numbering scheme.

Crystal data, data collection and structure refinement details are summarized in Table S1.

Table S1. Experimental details

Crystal data	
Chemical formula	$C_{25}H_{16}N_4O_3$
M_r	420.42
Crystal system, space group	Monoclinic, $P2_1/n$
Temperature (K)	150
a, *b*, *c* (Å)	9.25256 (17), 17.6794 (8), 12.9948 (3)
β (°)	110.477 (2)
V (Å^3)	1991.37 (11)
Z	4
Radiation type	Cu *K*α
μ (mm^{-1})	0.78
Crystal size (mm)	0.10 × 0.06 × 0.03
Data collection	

Diffractometer	XtaLAB Synergy, Dualflex, HyPix
Absorption correction	Multi-scan<br>*CrysAlis PRO* 1.171.42.93a (Rigaku Oxford Diffraction, 2023) Empirical absorption correction using spherical harmonics, implemented in SCALE3 ABSPACK scaling algorithm.
T_{min}, T_{max}	0.862, 1.000
No. of measured, independent and observed [$I > 2\sigma(I)$] reflections	10454, 3702, 3328
R_{int}	0.023
$(\sin \theta/\lambda)_{max}$ (Å^{-1})	0.630
Refinement	
$R[F^2 > 2\sigma(F^2)]$, $wR(F^2)$, S	0.033, 0.089, 1.06
No. of reflections	3702
No. of parameters	290
H-atom treatment	H-atom parameters constrained
$\Delta\rho_{max}$, $\Delta\rho_{min}$ (e Å^{-3})	0.21, -0.18

Computer programs: *CrysAlis PRO* 1.171.42.93a (Rigaku OD, 2023), SHELXT 2014/4 (Sheldrick, 2014), *SHELXL2018*/3 (Sheldrick, 2018).

References

Document origin: *publCIF* [Westrip, S. P. (2010). *J. Apply. Cryst.*, **43**, 920-925].

Computing details

Data collection: *CrysAlis PRO* 1.171.42.93a (Rigaku OD, 2023); cell refinement: *CrysAlis PRO* 1.171.42.93a (Rigaku OD, 2023); data reduction: *CrysAlis PRO* 1.171.42.93a (Rigaku OD, 2023); program(s) used to solve structure: SHELXT 2014/4 (Sheldrick, 2014); program(s) used to refine structure: *SHELXL2018*/3 (Sheldrick, 2018).

Crystal data

$C_{25}H_{16}N_4O_3$	$F(000) = 872$
$M_r = 420.42$	$D_x = 1.402$ Mg m^{-3}
Monoclinic, $P2_1/n$	Cu $K\alpha$ radiation, $\lambda = 1.54184$ Å
$a = 9.25256$ (17) Å	Cell parameters from 7265 reflections
$b = 17.6794$ (8) Å	$\theta = 4.4–75.7°$

c = 12.9948 (3) Å	μ = 0.78 mm^{-1}
β = 110.477 (2)°	T = 150 K
V = 1991.37 (11) Å^3	Block, colourless
Z = 4	0.10 × 0.06 × 0.03 mm

Data collection

XtaLAB Synergy, Dualflex, HyPix diffractometer	3328 reflections with $I > 2\sigma(I)$
Radiation source: micro-focus sealed X-ray tube	R_{int} = 0.023
ω scans	θ_{max} = 76.2°, θ_{min} = 4.4°
Absorption correction: multi-scan *CrysAlis PRO* 1.171.42.93a (Rigaku Oxford Diffraction, 2023) Empirical absorption correction using spherical harmonics, implemented in SCALE3 ABSPACK scaling algorithm.	h = -8→11
T_{min} = 0.862, T_{max} = 1.000	k = -9→21
10454 measured reflections	l = -15→15
3702 independent reflections	

Refinement

Refinement on F^2	Hydrogen site location: inferred from neighbouring sites
Least-squares matrix: full	H-atom parameters constrained
$R[F^2 > 2\sigma(F^2)]$ = 0.033	$w = 1/[\sigma^2(F_o^2) + (0.0424P)^2 + 0.5247P]$ where $P = (F_o^2 + 2F_c^2)/3$
$wR(F^2)$ = 0.089	$(\Delta/\sigma)_{max}$ < 0.001
S = 1.06	$\Delta\rho_{max}$ = 0.21 e Å^{-3}
3702 reflections	$\Delta\rho_{min}$ = -0.18 e Å^{-3}
290 parameters	Extinction correction: *SHELXL2018*/3 (Sheldrick 2018), $Fc^* = kFc[1+0.001xFc^2\lambda^3/\sin(2\theta)]^{-1/4}$
0 restraints	Extinction coefficient: 0.00116 (16)

Special details

Geometry. All esds (except the esd in the dihedral angle between two l.s. planes) are estimated using the full covariance matrix. The cell esds are taken into account individually in the estimation of esds in distances, angles and torsion angles; correlations between esds in cell parameters are only used when they are defined by crystal symmetry. An approximate (isotropic) treatment of cell esds is used for estimating esds involving l.s. planes.

Fractional atomic coordinates and isotropic or equivalent isotropic displacement parameters (Å^2)

	x	y	z	U_{iso}*/U_{eq}
O12	0.19668 (10)	0.18625 (5)	0.19380 (7)	0.0310 (2)
N27	0.38216 (11)	0.08465 (5)	0.43376 (8)	0.0241 (2)
O26	0.53609 (14)	0.14303 (6)	-0.14289 (9)	0.0479 (3)
N1	0.04646 (11)	0.03218 (5)	0.27556 (8)	0.0228 (2)
C9	-0.04374 (13)	0.07801 (7)	0.31927 (9)	0.0229 (3)
O25	0.72571 (12)	0.12880 (6)	0.01070 (10)	0.0480 (3)
N13	0.17442 (12)	0.01870 (6)	0.15216 (8)	0.0274 (2)
C8	-0.12503 (13)	0.03064 (7)	0.36696 (9)	0.0231 (3)
C17	-0.22337 (14)	0.06212 (7)	0.41564 (10)	0.0271 (3)
H17	-0.276345	0.030673	0.450036	0.033*
C2	0.01703 (13)	-0.04463 (7)	0.29271 (9)	0.0228 (3)
C10	0.14633 (13)	0.05821 (7)	0.22480 (9)	0.0228 (2)
C7	-0.08630 (13)	-0.04673 (7)	0.34998 (9)	0.0233 (3)
C6	-0.13232 (14)	-0.11621 (7)	0.37916 (10)	0.0283 (3)
H6	-0.201839	-0.118338	0.418290	0.034*
C19	0.43713 (15)	0.05443 (7)	0.16672 (10)	0.0291 (3)
H19	0.474407	0.042097	0.242677	0.035*
C16	-0.24286 (14)	0.13963 (7)	0.41325 (11)	0.0306 (3)
H16	-0.309460	0.161608	0.446430	0.037*
N24	0.58800 (14)	0.12543 (6)	-0.04516 (10)	0.0347 (3)
C15	-0.16559 (15)	0.18585 (7)	0.36255 (11)	0.0303 (3)
H15	-0.181607	0.238980	0.361022	0.036*
C14	-0.06589 (14)	0.15621 (7)	0.31427 (10)	0.0271 (3)
H14	-0.014536	0.187981	0.279075	0.033*
C11	0.21761 (13)	0.13608 (6)	0.26099 (10)	0.0229 (3)
C22	0.32666 (16)	0.08850 (7)	-0.05663 (11)	0.0314 (3)
H22	0.290124	0.100428	-0.132715	0.038*
C23	0.22737 (15)	0.06136 (8)	-0.00716 (11)	0.0308 (3)
H23	0.121790	0.053292	-0.049719	0.037*
C20	0.53819 (15)	0.08108 (7)	0.11758 (11)	0.0299 (3)
H20	0.644619	0.087540	0.159184	0.036*
C32	0.47768 (14)	0.09511 (7)	0.53748 (10)	0.0268 (3)
H32	0.528601	0.052227	0.578210	0.032*
C3	0.07615 (14)	-0.11038 (7)	0.26375 (10)	0.0275 (3)
H3	0.146419	-0.108612	0.225205	0.033*

C4	0.02812 (16)	-0.17854 (7)	0.29354 (11)	0.0325 (3)
H4	0.066543	-0.224378	0.274866	0.039*
C21	0.48087 (15)	0.09805 (7)	0.00688 (11)	0.0280 (3)
C18	0.28139 (14)	0.04570 (7)	0.10522 (10)	0.0258 (3)
C30	0.43411 (15)	0.22823 (7)	0.52993 (11)	0.0333 (3)
H30	0.450573	0.276834	0.563190	0.040*
C5	-0.07508 (16)	-0.18182 (7)	0.35020 (11)	0.0329 (3)
H5	-0.106141	-0.229528	0.368968	0.039*
C28	0.31625 (13)	0.14700 (6)	0.37829 (10)	0.0225 (2)
C29	0.33785 (14)	0.21911 (7)	0.42217 (11)	0.0290 (3)
H29	0.287723	0.261347	0.379281	0.035*
C31	0.50595 (14)	0.16514 (7)	0.58829 (11)	0.0306 (3)
H31	0.573807	0.169751	0.662261	0.037*

Atomic displacement parameters (Å^2)

	U^{11}	U^{22}	U^{33}	U^{12}	U^{13}	U^{23}
O12	0.0328 (5)	0.0292 (5)	0.0298 (5)	0.0024 (4)	0.0096 (4)	0.0086 (4)
N27	0.0243 (5)	0.0235 (5)	0.0244 (5)	-0.0002 (4)	0.0085 (4)	0.0015 (4)
O26	0.0600 (7)	0.0546 (7)	0.0364 (6)	-0.0094 (5)	0.0261 (5)	0.0075 (5)
N1	0.0220 (5)	0.0240 (5)	0.0233 (5)	-0.0007 (4)	0.0091 (4)	-0.0003 (4)
C9	0.0186 (5)	0.0282 (6)	0.0202 (6)	0.0000 (4)	0.0044 (4)	-0.0010 (4)
O25	0.0389 (6)	0.0540 (6)	0.0555 (7)	-0.0143 (5)	0.0223 (5)	0.0030 (5)
N13	0.0270 (5)	0.0326 (5)	0.0239 (5)	-0.0050 (4)	0.0106 (4)	-0.0041 (4)
C8	0.0201 (6)	0.0274 (6)	0.0196 (6)	-0.0015 (4)	0.0042 (4)	0.0004 (4)
C17	0.0228 (6)	0.0342 (6)	0.0253 (6)	-0.0024 (5)	0.0096 (5)	-0.0010 (5)
C2	0.0205 (6)	0.0258 (6)	0.0196 (6)	-0.0023 (4)	0.0041 (5)	0.0000 (4)
C10	0.0187 (5)	0.0279 (6)	0.0198 (6)	-0.0006 (4)	0.0044 (4)	0.0014 (5)
C7	0.0207 (6)	0.0285 (6)	0.0185 (6)	-0.0011 (4)	0.0042 (5)	-0.0005 (4)
C6	0.0273 (6)	0.0310 (6)	0.0270 (6)	-0.0030 (5)	0.0100 (5)	0.0024 (5)
C19	0.0291 (6)	0.0350 (7)	0.0229 (6)	-0.0025 (5)	0.0088 (5)	-0.0004 (5)
C16	0.0254 (6)	0.0360 (7)	0.0329 (7)	0.0018 (5)	0.0134 (5)	-0.0044 (5)
N24	0.0415 (7)	0.0285 (6)	0.0396 (7)	-0.0073 (5)	0.0213 (5)	-0.0017 (5)
C15	0.0280 (7)	0.0272 (6)	0.0342 (7)	0.0030 (5)	0.0092 (5)	-0.0014 (5)
C14	0.0251 (6)	0.0268 (6)	0.0294 (7)	-0.0001 (5)	0.0095 (5)	0.0020 (5)
C11	0.0199 (6)	0.0245 (6)	0.0259 (6)	0.0023 (4)	0.0101 (5)	0.0024 (5)
C22	0.0375 (7)	0.0339 (7)	0.0232 (6)	-0.0039 (5)	0.0111 (5)	0.0002 (5)
C23	0.0286 (7)	0.0373 (7)	0.0249 (6)	-0.0044 (5)	0.0073 (5)	-0.0024 (5)

C20	0.0257 (6)	0.0343 (7)	0.0299 (7)	-0.0038 (5)	0.0098 (5)	-0.0020 (5)
C32	0.0248 (6)	0.0294 (6)	0.0257 (6)	0.0023 (5)	0.0083 (5)	0.0037 (5)
C3	0.0261 (6)	0.0304 (6)	0.0262 (6)	0.0020 (5)	0.0096 (5)	-0.0015 (5)
C4	0.0345 (7)	0.0268 (6)	0.0358 (7)	0.0033 (5)	0.0117 (6)	-0.0020 (5)
C21	0.0336 (7)	0.0250 (6)	0.0298 (7)	-0.0040 (5)	0.0168 (5)	-0.0017 (5)
C18	0.0284 (6)	0.0262 (6)	0.0253 (6)	-0.0029 (5)	0.0125 (5)	-0.0043 (5)
C30	0.0295 (7)	0.0289 (6)	0.0373 (7)	-0.0003 (5)	0.0063 (6)	-0.0096 (5)
C5	0.0366 (7)	0.0257 (6)	0.0358 (7)	-0.0032 (5)	0.0121 (6)	0.0028 (5)
C28	0.0199 (5)	0.0235 (5)	0.0247 (6)	0.0001 (4)	0.0086 (5)	0.0010 (4)
C29	0.0273 (6)	0.0234 (6)	0.0341 (7)	0.0023 (5)	0.0079 (5)	-0.0003 (5)
C31	0.0243 (6)	0.0377 (7)	0.0263 (7)	-0.0012 (5)	0.0043 (5)	-0.0053 (5)

Geometric parameters (Å, º)

O12—C11	1.2117 (14)	C16—H16	0.9500
N27—C32	1.3411 (16)	N24—C21	1.4640 (16)
N27—C28	1.3411 (15)	C15—C14	1.3872 (17)
O26—N24	1.2302 (16)	C15—H15	0.9500
N1—C10	1.3882 (15)	C14—H14	0.9500
N1—C9	1.4167 (15)	C11—C28	1.4901 (16)
N1—C2	1.4179 (15)	C22—C23	1.3786 (18)
C9—C14	1.3958 (17)	C22—C21	1.3862 (19)
C9—C8	1.4062 (16)	C22—H22	0.9500
O25—N24	1.2276 (16)	C23—C18	1.3959 (18)
N13—C10	1.2721 (16)	C23—H23	0.9500
N13—C18	1.4149 (15)	C20—C21	1.3809 (18)
C8—C17	1.3930 (17)	C20—H20	0.9500
C8—C7	1.4504 (16)	C32—C31	1.3843 (18)
C17—C16	1.3810 (19)	C32—H32	0.9500
C17—H17	0.9500	C3—C4	1.3855 (18)
C2—C3	1.3914 (17)	C3—H3	0.9500
C2—C7	1.4024 (17)	C4—C5	1.396 (2)
C10—C11	1.5279 (16)	C4—H4	0.9500
C7—C6	1.3953 (17)	C30—C29	1.3811 (18)
C6—C5	1.3810 (19)	C30—C31	1.3814 (19)
C6—H6	0.9500	C30—H30	0.9500
C19—C20	1.3864 (18)	C5—H5	0.9500
C19—C18	1.3904 (18)	C28—C29	1.3823 (17)

C19—H19	0.9500	C29—H29	0.9500
C16—C15	1.3938 (18)	C31—H31	0.9500
C32—N27—C28	116.28 (10)	O12—C11—C10	119.66 (11)
C10—N1—C9	125.76 (10)	C28—C11—C10	118.39 (10)
C10—N1—C2	126.05 (10)	C23—C22—C21	118.67 (12)
C9—N1—C2	108.19 (9)	C23—C22—H22	120.7
C14—C9—C8	121.19 (11)	C21—C22—H22	120.7
C14—C9—N1	130.19 (11)	C22—C23—C18	120.25 (12)
C8—C9—N1	108.49 (10)	C22—C23—H23	119.9
C10—N13—C18	119.34 (10)	C18—C23—H23	119.9
C17—C8—C9	119.82 (11)	C21—C20—C19	118.59 (12)
C17—C8—C7	132.95 (11)	C21—C20—H20	120.7
C9—C8—C7	107.21 (10)	C19—C20—H20	120.7
C16—C17—C8	119.15 (12)	N27—C32—C31	123.42 (11)
C16—C17—H17	120.4	N27—C32—H32	118.3
C8—C17—H17	120.4	C31—C32—H32	118.3
C3—C2—C7	121.79 (11)	C4—C3—C2	117.14 (12)
C3—C2—N1	129.96 (11)	C4—C3—H3	121.4
C7—C2—N1	108.23 (10)	C2—C3—H3	121.4
N13—C10—N1	120.89 (11)	C3—C4—C5	121.93 (12)
N13—C10—C11	123.02 (11)	C3—C4—H4	119.0
N1—C10—C11	116.09 (10)	C5—C4—H4	119.0
C6—C7—C2	119.80 (11)	C20—C21—C22	122.28 (12)
C6—C7—C8	132.36 (12)	C20—C21—N24	118.54 (12)
C2—C7—C8	107.84 (10)	C22—C21—N24	119.17 (12)
C5—C6—C7	118.87 (12)	C19—C18—C23	119.94 (12)
C5—C6—H6	120.6	C19—C18—N13	122.03 (11)
C7—C6—H6	120.6	C23—C18—N13	118.00 (11)
C20—C19—C18	120.22 (12)	C29—C30—C31	118.57 (12)
C20—C19—H19	119.9	C29—C30—H30	120.7
C18—C19—H19	119.9	C31—C30—H30	120.7
C17—C16—C15	120.52 (12)	C6—C5—C4	120.47 (12)
C17—C16—H16	119.7	C6—C5—H5	119.8
C15—C16—H16	119.7	C4—C5—H5	119.8
O25—N24—O26	123.22 (12)	N27—C28—C29	124.28 (11)
O25—N24—C21	118.25 (11)	N27—C28—C11	116.36 (10)

O26—N24—C21	118.53 (12)	C29—C28—C11	119.33 (10)
C14—C15—C16	121.64 (12)	C30—C29—C28	118.34 (11)
C14—C15—H15	119.2	C30—C29—H29	120.8
C16—C15—H15	119.2	C28—C29—H29	120.8
C15—C14—C9	117.59 (11)	C30—C31—C32	119.07 (12)
C15—C14—H14	121.2	C30—C31—H31	120.5
C9—C14—H14	121.2	C32—C31—H31	120.5
O12—C11—C28	121.91 (11)		

Document origin: *publCIF* [Westrip, S. P. (2010). *J. Apply. Cryst.*, **43**, 920-925].

(*E*)-*N*-[1-(9*H*-Carbazol-9-yl)-2-(2-chloro-9-heptyl-9*H*-purin-6-yl)vinyl]-4-nitroaniline (5b)

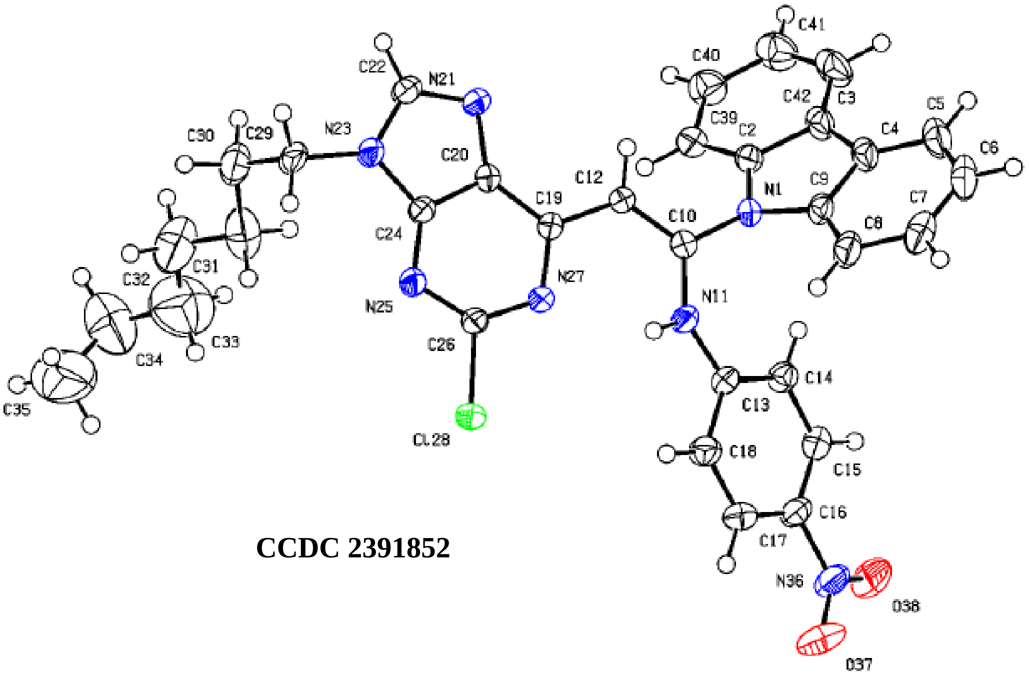

CCDC 2391852

Figure S2. ORTEP plot for **5b** with the atom numbering scheme.

Crystal data, data collection and structure refinement details are summarized in Table S2.

Table S2. Experimental details

Crystal data	
Chemical formula	$C_{32}H_{30}ClN_7O_2$
M_r	580.08
Crystal system, space group	Monoclinic, *Pc*
Temperature (K)	160
a, *b*, *c* (Å)	13.5064 (2), 7.9346 (1), 13.4492 (2)
β (°)	98.112 (1)
V (Å^3)	1426.90 (4)
Z	2
Radiation type	Cu *K*α
μ (mm^{-1})	1.54
Crystal size (mm)	0.25 × 0.12 × 0.10
Data collection	

Diffractometer	XtaLAB Synergy, Dualflex, HyPix
Absorption correction	Multi-scan<br>*CrysAlis PRO* 1.171.40.74a (Rigaku Oxford Diffraction, 2020) Empirical absorption correction using spherical harmonics, implemented in SCALE3 ABSPACK scaling algorithm.
T_{min}, T_{max}	0.723, 1.000
No. of measured, independent and observed [$I > 2\sigma(I)$] reflections	7258, 3271, 3203
R_{int}	0.021
$(\sin \theta/\lambda)_{max}$ (Å^{-1})	0.630
Refinement	
$R[F^2 > 2\sigma(F^2)]$, $wR(F^2)$, S	0.036, 0.097, 1.04
No. of reflections	3271
No. of parameters	381
No. of restraints	3
H-atom treatment	H-atom parameters constrained
$\Delta\rho_{max}$, $\Delta\rho_{min}$ (e Å^{-3})	0.46, -0.28
Absolute structure	Flack x determined using 600 quotients [(I+)-(I-)]/[(I+)+(I-)] (Parsons, Flack and Wagner, Acta Cryst. B69 (2013) 249-259).
Absolute structure parameter	0.029 (12)

Computer programs: *CrysAlis PRO* 1.171.40.74a (Rigaku OD, 2020), SHELXT 2014/4 (Sheldrick, 2014), *SHELXL2018*/3 (Sheldrick, 2018).

References

Document origin: *publCIF* [Westrip, S. P. (2010). *J. Apply. Cryst.*, **43**, 920-925].

Computing details

Data collection: *CrysAlis PRO* 1.171.40.74a (Rigaku OD, 2020); cell refinement: *CrysAlis PRO* 1.171.40.74a (Rigaku OD, 2020); data reduction: *CrysAlis PRO* 1.171.40.74a (Rigaku OD, 2020); program(s) used to solve structure: SHELXT 2014/4 (Sheldrick, 2014); program(s) used to refine structure: *SHELXL2018*/3 (Sheldrick, 2018).

Crystal data

$C_{32}H_{30}ClN_7O_2$	$F(000) = 608$
$M_r = 580.08$	$D_x = 1.350$ Mg m^{-3}
Monoclinic, *Pc*	Cu $K\alpha$ radiation, $\lambda = 1.54184$ Å
$a = 13.5064$ (2) Å	Cell parameters from 6809 reflections
$b = 7.9346$ (1) Å	$\theta = 3.3$–$75.6°$
$c = 13.4492$ (2) Å	$\mu = 1.54$ mm^{-1}
$\beta = 98.112$ (1)°	$T = 160$ K
$V = 1426.90$ (4) Å^3	Block, colourless
$Z = 2$	0.25 × 0.12 × 0.10 mm

Data collection

XtaLAB Synergy, Dualflex, HyPix diffractometer	3203 reflections with $I > 2\sigma(I)$
Radiation source: micro-focus sealed X-ray tube	$R_{int} = 0.021$
ω scans	$\theta_{max} = 76.3°$, $\theta_{min} = 3.3°$
Absorption correction: multi-scan *CrysAlis PRO* 1.171.40.74a (Rigaku Oxford Diffraction, 2020) Empirical absorption correction using spherical harmonics, implemented in SCALE3 ABSPACK scaling algorithm.	$h = -16 \rightarrow 13$
$T_{min} = 0.723$, $T_{max} = 1.000$	$k = -9 \rightarrow 10$
7258 measured reflections	$l = -11 \rightarrow 16$
3271 independent reflections	

Refinement

Refinement on F^2	H-atom parameters constrained
Least-squares matrix: full	$w = 1/[\sigma^2(F_o^2) + (0.0577P)^2 + 0.5479P]$ where $P = (F_o^2 + 2F_c^2)/3$
$R[F^2 > 2\sigma(F^2)] = 0.036$	$(\Delta/\sigma)_{max} < 0.001$
$wR(F^2) = 0.097$	$\Delta\rho_{max} = 0.46$ e Å^{-3}

S = 1.04	$\Delta\rho_{min}$ = -0.28 e Å^{-3}
3271 reflections	Extinction correction: *SHELXL2018*/3 (Sheldrick 2018), $Fc^*=kFc[1+0.001xFc^2\lambda^3/\sin(2\theta)]^{-1/4}$
381 parameters	Extinction coefficient: 0.0018 (4)
3 restraints	Absolute structure: Flack x determined using 600 quotients [(I+)-(I-)]/[(I+)+(I-)] (Parsons, Flack and Wagner, Acta Cryst. B69 (2013) 249-259).
Hydrogen site location: inferred from neighbouring sites	Absolute structure parameter: 0.029 (12)

Special details

Geometry. All esds (except the esd in the dihedral angle between two l.s. planes) are estimated using the full covariance matrix. The cell esds are taken into account individually in the estimation of esds in distances, angles and torsion angles; correlations between esds in cell parameters are only used when they are defined by crystal symmetry. An approximate (isotropic) treatment of cell esds is used for estimating esds involving l.s. planes.

Fractional atomic coordinates and isotropic or equivalent isotropic displacement parameters (Å^2)

	x	*y*	*z*	U_{iso}*/U_{eq}
Cl28	0.45131 (5)	0.76608 (9)	0.21853 (6)	0.03144 (19)
O38	0.0755 (2)	0.1329 (4)	0.5574 (3)	0.0582 (8)
N25	0.62306 (18)	0.8928 (3)	0.29179 (19)	0.0266 (5)
N23	0.7796 (2)	1.0038 (3)	0.3781 (2)	0.0308 (6)
N1	0.4453 (2)	0.7464 (3)	0.7099 (2)	0.0290 (6)
N27	0.51269 (18)	0.8078 (3)	0.40759 (19)	0.0252 (5)
N36	0.0813 (2)	0.2358 (4)	0.4903 (3)	0.0428 (7)
N11	0.38730 (19)	0.7165 (3)	0.5351 (2)	0.0300 (6)
H11	0.387871	0.771783	0.478450	0.036*
N21	0.7520 (2)	0.9745 (4)	0.5386 (2)	0.0329 (6)
C24	0.6867 (2)	0.9350 (4)	0.3742 (2)	0.0254 (6)
O37	0.0244 (2)	0.2358 (4)	0.4108 (3)	0.0643 (9)
C9	0.3677 (2)	0.8243 (4)	0.7535 (3)	0.0313 (7)
C26	0.5404 (2)	0.8299 (4)	0.3177 (2)	0.0246 (6)
C2	0.5198 (2)	0.6947 (4)	0.7891 (2)	0.0295 (6)
C13	0.3116 (2)	0.5988 (4)	0.5313 (2)	0.0272 (6)
C20	0.6701 (2)	0.9175 (4)	0.4741 (2)	0.0262 (6)
C16	0.1592 (2)	0.3623 (4)	0.5042 (3)	0.0331 (7)
C39	0.6090 (3)	0.6100 (4)	0.7841 (3)	0.0351 (7)
H39	0.629199	0.580986	0.721483	0.042*

C29	0.8301 (3)	1.0466 (5)	0.2917 (3)	0.0410 (8)
H29A	0.863545	1.157103	0.304134	0.049*
H29B	0.779162	1.058223	0.231451	0.049*
C14	0.2891 (2)	0.5031 (4)	0.6127 (2)	0.0289 (6)
H14	0.325328	0.520264	0.677686	0.035*
C10	0.4620 (2)	0.7643 (4)	0.6100 (2)	0.0275 (6)
C8	0.2800 (3)	0.8976 (4)	0.7068 (3)	0.0391 (8)
H8	0.263288	0.897468	0.635770	0.047*
C17	0.1791 (3)	0.4570 (4)	0.4225 (3)	0.0364 (7)
H17	0.140963	0.441529	0.358241	0.044*
C3	0.4877 (3)	0.7360 (4)	0.8811 (3)	0.0342 (8)
C12	0.5485 (2)	0.8346 (4)	0.5888 (2)	0.0276 (6)
H12	0.593851	0.876046	0.643812	0.033*
C4	0.3926 (3)	0.8195 (4)	0.8583 (3)	0.0348 (7)
C18	0.2551 (2)	0.5738 (4)	0.4364 (3)	0.0343 (7)
H18	0.269671	0.638674	0.380874	0.041*
C22	0.8139 (2)	1.0240 (4)	0.4778 (2)	0.0341 (7)
H22	0.877797	1.070439	0.501297	0.041*
C15	0.2140 (2)	0.3838 (4)	0.5981 (3)	0.0321 (7)
H15	0.200098	0.316241	0.652804	0.039*
C19	0.5771 (2)	0.8517 (4)	0.4904 (2)	0.0253 (6)
C7	0.2177 (3)	0.9715 (4)	0.7685 (3)	0.0458 (9)
H7	0.157295	1.023665	0.738985	0.055*
C5	0.3294 (3)	0.8950 (5)	0.9189 (3)	0.0444 (9)
H5	0.345766	0.894756	0.989957	0.053*
C41	0.6378 (3)	0.6112 (5)	0.9655 (3)	0.0500 (10)
H41	0.679839	0.582343	1.025798	0.060*
C42	0.5489 (4)	0.6938 (5)	0.9711 (3)	0.0488 (9)
H42	0.529488	0.721630	1.034292	0.059*
C40	0.6674 (3)	0.5693 (5)	0.8744 (3)	0.0455 (9)
H40	0.728902	0.511563	0.873474	0.055*
C6	0.2422 (3)	0.9703 (5)	0.8730 (3)	0.0497 (10)
H6	0.198379	1.022038	0.913188	0.060*
C31	0.8620 (4)	0.7506 (6)	0.2439 (4)	0.0639 (13)
H31A	0.803019	0.763251	0.191684	0.077*
H31B	0.839031	0.699951	0.303954	0.077*
C30	0.9052 (3)	0.9204 (7)	0.2706 (3)	0.0568 (11)

H30A	0.956542	0.909613	0.330546	0.068*
H30B	0.938952	0.961784	0.214573	0.068*
C32	0.9365 (5)	0.6335 (7)	0.2057 (6)	0.0842 (18)
H32A	0.954388	0.680622	0.142380	0.101*
H32B	0.998131	0.631937	0.255209	0.101*
C34	0.9752 (7)	0.3454 (9)	0.1388 (6)	0.103 (2)
H34A	1.045436	0.379373	0.160436	0.124*
H34B	0.966807	0.228036	0.161124	0.124*
C33	0.9037 (8)	0.4641 (10)	0.1874 (8)	0.126 (3)
H33A	0.838549	0.466757	0.143191	0.151*
H33B	0.891956	0.413965	0.252131	0.151*
C35	0.9532 (9)	0.3547 (14)	0.0327 (8)	0.167 (5)
H35A	1.001479	0.286351	0.002411	0.251*
H35B	0.957490	0.472172	0.011270	0.251*
H35C	0.885510	0.311955	0.011220	0.251*

Atomic displacement parameters (Å^2)

	U^{11}	U^{22}	U^{33}	U^{12}	U^{13}	U^{23}
Cl28	0.0266 (3)	0.0426 (4)	0.0240 (3)	-0.0044 (3)	-0.0002 (2)	-0.0038 (3)
O38	0.0514 (17)	0.0541 (16)	0.072 (2)	-0.0249 (14)	0.0185 (15)	-0.0003 (15)
N25	0.0242 (12)	0.0331 (12)	0.0228 (13)	-0.0011 (10)	0.0048 (10)	0.0009 (10)
N23	0.0262 (13)	0.0450 (15)	0.0220 (13)	-0.0086 (11)	0.0057 (10)	0.0022 (11)
N1	0.0261 (13)	0.0393 (12)	0.0231 (14)	-0.0070 (11)	0.0089 (10)	-0.0051 (11)
N27	0.0227 (12)	0.0290 (11)	0.0241 (13)	-0.0044 (10)	0.0046 (9)	-0.0022 (10)
N36	0.0254 (14)	0.0466 (16)	0.057 (2)	-0.0086 (12)	0.0071 (13)	-0.0100 (15)
N11	0.0245 (13)	0.0396 (13)	0.0259 (15)	-0.0092 (11)	0.0039 (11)	0.0010 (11)
N21	0.0242 (13)	0.0489 (15)	0.0256 (14)	-0.0108 (11)	0.0034 (10)	-0.0040 (12)
C24	0.0225 (14)	0.0294 (13)	0.0244 (15)	-0.0031 (11)	0.0034 (11)	0.0006 (12)
O37	0.0392 (16)	0.0699 (19)	0.079 (2)	-0.0227 (14)	-0.0086 (15)	-0.0090 (16)
C9	0.0323 (16)	0.0306 (14)	0.0342 (17)	-0.0098 (12)	0.0157 (13)	-0.0038 (13)
C26	0.0211 (13)	0.0277 (13)	0.0248 (15)	0.0008 (10)	0.0020 (11)	-0.0016 (12)
C2	0.0337 (17)	0.0313 (14)	0.0246 (16)	-0.0095 (13)	0.0077 (12)	-0.0011 (12)
C13	0.0205 (13)	0.0311 (14)	0.0300 (16)	-0.0011 (11)	0.0037 (11)	-0.0008 (12)
C20	0.0246 (14)	0.0325 (14)	0.0222 (15)	-0.0037 (11)	0.0058 (11)	-0.0011 (12)
C16	0.0225 (14)	0.0317 (14)	0.045 (2)	-0.0044 (12)	0.0062 (13)	-0.0062 (13)
C39	0.0337 (17)	0.0423 (16)	0.0296 (17)	-0.0059 (14)	0.0052 (13)	-0.0034 (14)
C29	0.0313 (17)	0.065 (2)	0.0273 (17)	-0.0198 (16)	0.0076 (13)	0.0053 (16)

C14	0.0255 (15)	0.0313 (14)	0.0296 (17)	0.0008 (11)	0.0026 (12)	0.0027 (12)
C10	0.0260 (15)	0.0323 (14)	0.0247 (16)	-0.0031 (11)	0.0058 (12)	-0.0037 (12)
C8	0.0354 (18)	0.0392 (17)	0.046 (2)	-0.0060 (14)	0.0186 (15)	-0.0011 (15)
C17	0.0299 (16)	0.0433 (17)	0.0342 (18)	-0.0055 (14)	-0.0020 (13)	-0.0040 (15)
C3	0.047 (2)	0.0311 (15)	0.0269 (18)	-0.0097 (13)	0.0153 (15)	-0.0004 (13)
C12	0.0240 (14)	0.0361 (15)	0.0233 (15)	-0.0075 (12)	0.0048 (11)	-0.0046 (13)
C4	0.0459 (19)	0.0292 (15)	0.0338 (19)	-0.0114 (13)	0.0217 (14)	-0.0044 (13)
C18	0.0336 (17)	0.0392 (16)	0.0292 (17)	-0.0067 (14)	0.0013 (13)	0.0019 (14)
C22	0.0256 (16)	0.0488 (18)	0.0275 (17)	-0.0119 (13)	0.0025 (12)	0.0012 (14)
C15	0.0297 (15)	0.0308 (14)	0.0374 (19)	0.0003 (12)	0.0100 (13)	0.0019 (13)
C19	0.0229 (14)	0.0300 (13)	0.0236 (15)	-0.0024 (11)	0.0053 (11)	-0.0011 (12)
C7	0.0384 (19)	0.0357 (16)	0.068 (3)	-0.0023 (14)	0.0255 (18)	0.0030 (17)
C5	0.061 (2)	0.0372 (17)	0.041 (2)	-0.0089 (16)	0.0290 (18)	-0.0057 (16)
C41	0.059 (3)	0.055 (2)	0.034 (2)	-0.0006 (19)	-0.0014 (18)	0.0062 (18)
C42	0.073 (3)	0.050 (2)	0.0247 (19)	-0.0121 (19)	0.0129 (18)	0.0010 (16)
C40	0.047 (2)	0.0488 (19)	0.039 (2)	-0.0031 (17)	0.0008 (16)	0.0052 (18)
C6	0.060 (3)	0.0365 (17)	0.063 (3)	-0.0064 (17)	0.043 (2)	-0.0070 (17)
C31	0.063 (3)	0.080 (3)	0.052 (3)	0.018 (2)	0.022 (2)	0.012 (2)
C30	0.035 (2)	0.092 (3)	0.045 (2)	-0.004 (2)	0.0141 (16)	0.003 (2)
C32	0.068 (3)	0.079 (3)	0.113 (5)	-0.004 (3)	0.038 (3)	0.005 (3)
C34	0.145 (7)	0.087 (4)	0.085 (5)	0.007 (4)	0.040 (4)	0.001 (4)
C33	0.135 (7)	0.095 (5)	0.147 (8)	0.016 (5)	0.018 (6)	-0.026 (5)
C35	0.208 (12)	0.192 (10)	0.108 (7)	-0.138 (10)	0.044 (7)	-0.016 (7)

Geometric parameters (Å, º)

Cl28—C26	1.741 (3)	C17—C18	1.376 (5)
O38—N36	1.227 (5)	C17—H17	0.9500
N25—C26	1.315 (4)	C3—C42	1.407 (6)
N25—C24	1.345 (4)	C3—C4	1.438 (5)
N23—C24	1.363 (4)	C12—C19	1.437 (4)
N23—C22	1.365 (4)	C12—H12	0.9500
N23—C29	1.469 (4)	C4—C5	1.396 (5)
N1—C10	1.400 (5)	C18—H18	0.9500
N1—C9	1.413 (4)	C22—H22	0.9500
N1—C2	1.419 (5)	C15—H15	0.9500
N27—C26	1.326 (4)	C7—C6	1.398 (6)
N27—C19	1.358 (4)	C7—H7	0.9500

N36—O37	1.225 (5)	C5—C6	1.386 (7)
N36—C16	1.447 (4)	C5—H5	0.9500
N11—C10	1.375 (4)	C41—C42	1.380 (6)
N11—C13	1.381 (4)	C41—C40	1.381 (6)
N11—H11	0.8800	C41—H41	0.9500
N21—C22	1.310 (4)	C42—H42	0.9500
N21—C20	1.382 (4)	C40—H40	0.9500
C24—C20	1.400 (4)	C6—H6	0.9500
C9—C8	1.387 (5)	C31—C30	1.492 (7)
C9—C4	1.403 (5)	C31—C32	1.513 (7)
C2—C39	1.390 (5)	C31—H31A	0.9900
C2—C3	1.406 (5)	C31—H31B	0.9900
C13—C14	1.400 (5)	C30—H30A	0.9900
C13—C18	1.405 (4)	C30—H30B	0.9900
C20—C19	1.405 (4)	C32—C33	1.426 (10)
C16—C15	1.382 (5)	C32—H32A	0.9900
C16—C17	1.388 (5)	C32—H32B	0.9900
C39—C40	1.389 (5)	C34—C35	1.417 (12)
C39—H39	0.9500	C34—C33	1.557 (11)
C29—C30	1.481 (6)	C34—H34A	0.9900
C29—H29A	0.9900	C34—H34B	0.9900
C29—H29B	0.9900	C33—H33A	0.9900
C14—C15	1.380 (4)	C33—H33B	0.9900
C14—H14	0.9500	C35—H35A	0.9800
C10—C12	1.361 (4)	C35—H35B	0.9800
C8—C7	1.392 (5)	C35—H35C	0.9800
C8—H8	0.9500		
C26—N25—C24	110.1 (2)	C17—C18—C13	121.1 (3)
C24—N23—C22	105.6 (2)	C17—C18—H18	119.4
C24—N23—C29	126.2 (3)	C13—C18—H18	119.4
C22—N23—C29	128.3 (3)	N21—C22—N23	114.8 (3)
C10—N1—C9	125.7 (3)	N21—C22—H22	122.6
C10—N1—C2	123.8 (3)	N23—C22—H22	122.6
C9—N1—C2	107.7 (3)	C14—C15—C16	120.1 (3)
C26—N27—C19	118.9 (3)	C14—C15—H15	119.9
O37—N36—O38	123.0 (3)	C16—C15—H15	119.9

O37—N36—C16	117.9 (3)	N27—C19—C20	116.8 (3)
O38—N36—C16	119.1 (3)	N27—C19—C12	120.4 (3)
C10—N11—C13	132.6 (3)	C20—C19—C12	122.8 (3)
C10—N11—H11	113.7	C8—C7—C6	121.2 (4)
C13—N11—H11	113.7	C8—C7—H7	119.4
C22—N21—C20	103.4 (3)	C6—C7—H7	119.4
N25—C24—N23	127.5 (3)	C6—C5—C4	118.4 (4)
N25—C24—C20	126.6 (3)	C6—C5—H5	120.8
N23—C24—C20	105.9 (3)	C4—C5—H5	120.8
C8—C9—C4	122.3 (3)	C42—C41—C40	121.7 (4)
C8—C9—N1	129.1 (3)	C42—C41—H41	119.1
C4—C9—N1	108.5 (3)	C40—C41—H41	119.1
N25—C26—N27	130.7 (3)	C41—C42—C3	118.4 (4)
N25—C26—Cl28	115.3 (2)	C41—C42—H42	120.8
N27—C26—Cl28	114.0 (2)	C3—C42—H42	120.8
C39—C2—C3	122.2 (3)	C41—C40—C39	121.3 (4)
C39—C2—N1	129.1 (3)	C41—C40—H40	119.4
C3—C2—N1	108.6 (3)	C39—C40—H40	119.4
N11—C13—C14	125.4 (3)	C5—C6—C7	121.2 (3)
N11—C13—C18	115.7 (3)	C5—C6—H6	119.4
C14—C13—C18	118.9 (3)	C7—C6—H6	119.4
N21—C20—C24	110.4 (3)	C30—C31—C32	112.3 (4)
N21—C20—C19	132.7 (3)	C30—C31—H31A	109.1
C24—C20—C19	117.0 (3)	C32—C31—H31A	109.1
C15—C16—C17	121.2 (3)	C30—C31—H31B	109.1
C15—C16—N36	119.3 (3)	C32—C31—H31B	109.1
C17—C16—N36	119.5 (3)	H31A—C31—H31B	107.9
C40—C39—C2	117.4 (3)	C29—C30—C31	113.6 (3)
C40—C39—H39	121.3	C29—C30—H30A	108.8
C2—C39—H39	121.3	C31—C30—H30A	108.8
N23—C29—C30	113.7 (3)	C29—C30—H30B	108.8
N23—C29—H29A	108.8	C31—C30—H30B	108.8
C30—C29—H29A	108.8	H30A—C30—H30B	107.7
N23—C29—H29B	108.8	C33—C32—C31	115.6 (6)
C30—C29—H29B	108.8	C33—C32—H32A	108.4
H29A—C29—H29B	107.7	C31—C32—H32A	108.4
C15—C14—C13	119.8 (3)	C33—C32—H32B	108.4

C15—C14—H14	120.1	C31—C32—H32B	108.4
C13—C14—H14	120.1	H32A—C32—H32B	107.4
C12—C10—N11	121.5 (3)	C35—C34—C33	109.7 (9)
C12—C10—N1	120.1 (3)	C35—C34—H34A	109.7
N11—C10—N1	118.3 (3)	C33—C34—H34A	109.7
C9—C8—C7	117.1 (4)	C35—C34—H34B	109.7
C9—C8—H8	121.4	C33—C34—H34B	109.7
C7—C8—H8	121.4	H34A—C34—H34B	108.2
C18—C17—C16	118.8 (3)	C32—C33—C34	116.5 (8)
C18—C17—H17	120.6	C32—C33—H33A	108.2
C16—C17—H17	120.6	C34—C33—H33A	108.2
C2—C3—C42	119.0 (4)	C32—C33—H33B	108.2
C2—C3—C4	107.3 (3)	C34—C33—H33B	108.2
C42—C3—C4	133.7 (3)	H33A—C33—H33B	107.3
C10—C12—C19	125.6 (3)	C34—C35—H35A	109.5
C10—C12—H12	117.2	C34—C35—H35B	109.5
C19—C12—H12	117.2	H35A—C35—H35B	109.5
C5—C4—C9	119.7 (4)	C34—C35—H35C	109.5
C5—C4—C3	132.4 (4)	H35A—C35—H35C	109.5
C9—C4—C3	107.9 (3)	H35B—C35—H35C	109.5
C26—N25—C24—N23	-180.0 (3)	C39—C2—C3—C42	1.6 (5)
C26—N25—C24—C20	-0.3 (4)	N1—C2—C3—C42	178.9 (3)
C22—N23—C24—N25	179.7 (3)	C39—C2—C3—C4	-179.0 (3)
C29—N23—C24—N25	0.0 (5)	N1—C2—C3—C4	-1.7 (3)
C22—N23—C24—C20	0.0 (3)	N11—C10—C12—C19	-6.7 (5)
C29—N23—C24—C20	-179.7 (3)	N1—C10—C12—C19	176.4 (3)
C10—N1—C9—C8	17.2 (5)	C8—C9—C4—C5	-2.0 (5)
C2—N1—C9—C8	178.6 (3)	N1—C9—C4—C5	177.4 (3)
C10—N1—C9—C4	-162.1 (3)	C8—C9—C4—C3	-179.7 (3)
C2—N1—C9—C4	-0.7 (3)	N1—C9—C4—C3	-0.4 (3)
C24—N25—C26—N27	1.6 (4)	C2—C3—C4—C5	-176.1 (3)
C24—N25—C26—Cl28	-179.0 (2)	C42—C3—C4—C5	3.2 (6)
C19—N27—C26—N25	-1.3 (5)	C2—C3—C4—C9	1.3 (3)
C19—N27—C26—Cl28	179.3 (2)	C42—C3—C4—C9	-179.5 (4)
C10—N1—C2—C39	-19.6 (5)	C16—C17—C18—C13	-0.5 (5)
C9—N1—C2—C39	178.6 (3)	N11—C13—C18—C17	178.5 (3)

C10—N1—C2—C3	163.3 (3)	C14—C13—C18—C17	-0.3 (5)
C9—N1—C2—C3	1.5 (3)	C20—N21—C22—N23	-0.2 (4)
C10—N11—C13—C14	5.1 (5)	C24—N23—C22—N21	0.1 (4)
C10—N11—C13—C18	-173.6 (3)	C29—N23—C22—N21	179.9 (3)
C22—N21—C20—C24	0.2 (4)	C13—C14—C15—C16	-2.0 (5)
C22—N21—C20—C19	-178.3 (3)	C17—C16—C15—C14	1.2 (5)
N25—C24—C20—N21	-179.9 (3)	N36—C16—C15—C14	179.6 (3)
N23—C24—C20—N21	-0.2 (3)	C26—N27—C19—C20	-0.4 (4)
N25—C24—C20—C19	-1.1 (5)	C26—N27—C19—C12	178.0 (3)
N23—C24—C20—C19	178.6 (3)	N21—C20—C19—N27	179.8 (3)
O37—N36—C16—C15	169.6 (3)	C24—C20—C19—N27	1.4 (4)
O38—N36—C16—C15	-11.2 (5)	N21—C20—C19—C12	1.5 (5)
O37—N36—C16—C17	-12.0 (5)	C24—C20—C19—C12	-177.0 (3)
O38—N36—C16—C17	167.3 (3)	C10—C12—C19—N27	5.3 (5)
C3—C2—C39—C40	-1.2 (5)	C10—C12—C19—C20	-176.4 (3)
N1—C2—C39—C40	-177.9 (3)	C9—C8—C7—C6	-0.5 (5)
C24—N23—C29—C30	-100.7 (4)	C9—C4—C5—C6	1.2 (5)
C22—N23—C29—C30	79.7 (4)	C3—C4—C5—C6	178.3 (3)
N11—C13—C14—C15	-177.1 (3)	C40—C41—C42—C3	-0.1 (6)
C18—C13—C14—C15	1.6 (5)	C2—C3—C42—C41	-0.9 (5)
C13—N11—C10—C12	155.2 (3)	C4—C3—C42—C41	179.9 (4)
C13—N11—C10—N1	-27.9 (5)	C42—C41—C40—C39	0.5 (6)
C9—N1—C10—C12	117.5 (3)	C2—C39—C40—C41	0.1 (5)
C2—N1—C10—C12	-41.1 (4)	C4—C5—C6—C7	-0.1 (5)
C9—N1—C10—N11	-59.5 (4)	C8—C7—C6—C5	-0.2 (6)
C2—N1—C10—N11	141.9 (3)	N23—C29—C30—C31	62.3 (5)
C4—C9—C8—C7	1.6 (5)	C32—C31—C30—C29	170.1 (5)
N1—C9—C8—C7	-177.6 (3)	C30—C31—C32—C33	174.4 (7)
C15—C16—C17—C18	0.1 (5)	C31—C32—C33—C34	174.6 (7)
N36—C16—C17—C18	-178.4 (3)	C35—C34—C33—C32	-89.3 (10)

Document origin: *publCIF* [Westrip, S. P. (2010). *J. Apply. Cryst.*, **43**, 920-925].

(*E*)-*N*-(1-(9*H*-Carbazol-9-yl)-2-(9-heptyl-2-(1-phenyl-1*H*-1,2,3-triazol-4-yl)-9*H*-purin-6-yl)vinyl)aniline (5h)

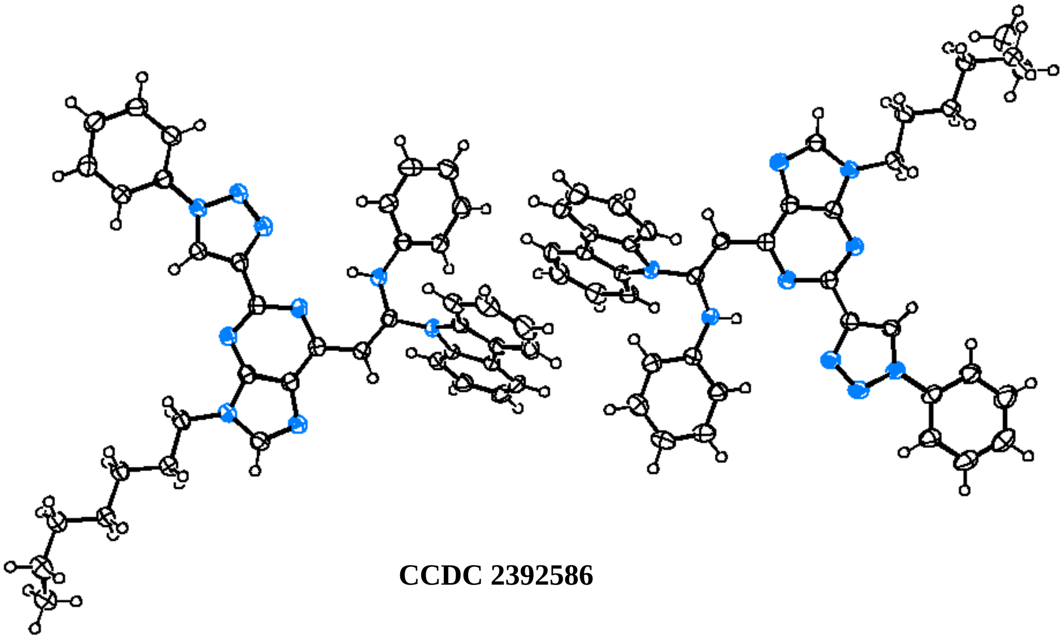

CCDC 2392586

Figure S5. ORTEP view of compound **5h** asymmetric unit.

Crystal data, data collection and structure refinement are summarized in Table S5.

Table S5. Experimental details

Crystal data	
Chemical formula	$C_{80}H_{74}N_{18}$
M_r	1287.57
Crystal system, space group	Monoclinic, $P2_1/n$
Temperature (K)	170
a, *b*, *c* (Å)	13.8681 (2), 27.8979 (3), 17.4886 (2)
β (°)	98.836 (1)
V (Å^3)	6685.88 (14)
Z	4
Radiation type	Cu *K*α
μ (mm^{-1})	0.62
Crystal size (mm)	0.25 × 0.08 × 0.05

Data collection	
Diffractometer	XtaLAB Synergy, Dualflex, HyPix
Absorption correction	Multi-scan<br>*CrysAlis PRO* 1.171.40.71a (Rigaku Oxford Diffraction, 2020) Empirical absorption correction using spherical harmonics, implemented in SCALE3 ABSPACK scaling algorithm.
T_{min}, T_{max}	0.579, 1.000
No. of measured, independent and observed [$I > 2\sigma(I)$] reflections	64830, 13209, 10940
R_{int}	0.043
$(\sin \theta/\lambda)_{max}$ (Å^{-1})	0.631
Refinement	
$R[F^2 > 2\sigma(F^2)]$, $wR(F^2)$, S	0.042, 0.116, 1.03
No. of reflections	13209
No. of parameters	885
H-atom treatment	H-atom parameters constrained
$\Delta\rho_{max}$, $\Delta\rho_{min}$ (e Å^{-3})	0.18, -0.25

Computer programs: *CrysAlis PRO* 1.171.42.93a (Rigaku OD, 2023), SHELXT 2014/4 (Sheldrick, 2014), *SHELXL2018*/3 (Sheldrick, 2018).

References

Document origin: *publCIF* [Westrip, S. P. (2010). *J. Apply. Cryst.*, **43**, 920-925].

9-[3-(2-Chloro-9-heptyl-9*H*-purin-6-yl)-5-nitro-1*H*-indol-2-yl]-9*H*-carbazole (6b)

CCDC 2391858

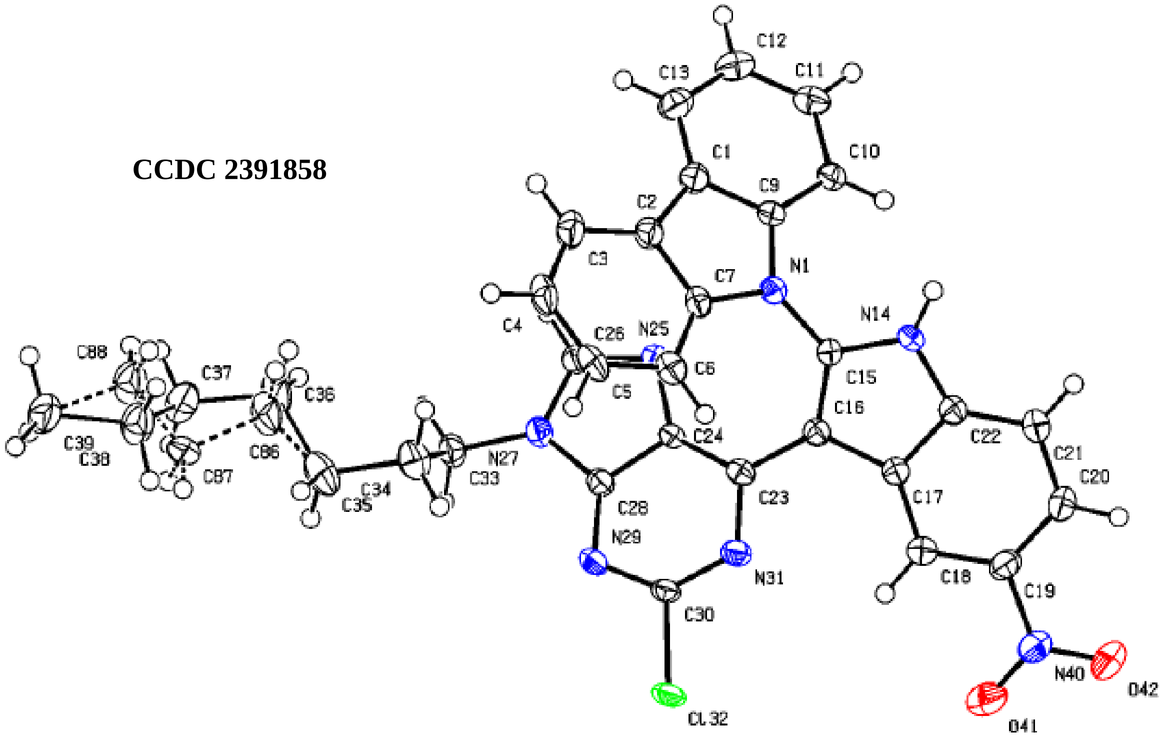

Figure S3. ORTEP plot for **6b** with the atom numbering scheme, showing disorder in *n*-C_7H_{15} fragment. Crystal data, data collection and structure refinement details are summarized in Table S3.

Table S3. Experimental details

Crystal data	
Chemical formula	$C_{32}H_{28}ClN_7O_2$
M_r	578.06
Crystal system, space group	Monoclinic, $P2_1/c$
Temperature (K)	150
a, *b*, *c* (Å)	13.0778 (2), 7.5547 (1), 28.6597 (4)
β (°)	101.588 (1)
V (Å^3)	2773.83 (7)
Z	4
Radiation type	Cu *K*α
μ (mm^{-1})	1.58
Crystal size (mm)	0.20 × 0.12 × 0.08
Data collection	
Diffractometer	XtaLAB Synergy, Dualflex, HyPix

Absorption correction	Multi-scan<br>*CrysAlis PRO* 1.171.41.123a (Rigaku Oxford Diffraction, 2022) Empirical absorption correction using spherical harmonics, implemented in SCALE3 ABSPACK scaling algorithm.
T_{min}, T_{max}	0.783, 1.000
No. of measured, independent and observed [$I > 2\sigma(I)$] reflections	26402, 5448, 5074
R_{int}	0.025
$(\sin \theta/\lambda)_{max}$ (Å^{-1})	0.631
Refinement	
$R[F^2 > 2\sigma(F^2)]$, $wR(F^2)$, S	0.070, 0.192, 1.05
No. of reflections	5448
No. of parameters	395
No. of restraints	10
H-atom treatment	H-atom parameters constrained
$\Delta\rho_{max}$, $\Delta\rho_{min}$ (e Å^{-3})	0.96, -0.48

Computer programs: *CrysAlis PRO* 1.171.41.123a (Rigaku OD, 2022), SHELXT 2014/4 (Sheldrick, 2014), *SHELXL2018*/3 (Sheldrick, 2018).

References

Document origin: *publCIF* [Westrip, S. P. (2010). *J. Apply. Cryst.*, **43**, 920-925].

Computing details

Data collection: *CrysAlis PRO* 1.171.41.123a (Rigaku OD, 2022); cell refinement: *CrysAlis PRO* 1.171.41.123a (Rigaku OD, 2022); data reduction: *CrysAlis PRO* 1.171.41.123a (Rigaku OD, 2022); program(s) used to solve structure: SHELXT 2014/4 (Sheldrick, 2014); program(s) used to refine structure: *SHELXL2018*/3 (Sheldrick, 2018).

Crystal data

$C_{32}H_{28}ClN_7O_2$	$F(000) = 1208$
$M_r = 578.06$	$D_x = 1.384$ Mg m^{-3}
Monoclinic, $P2_1/c$	Cu $K\alpha$ radiation, $\lambda = 1.54184$ Å
$a = 13.0778$ (2) Å	Cell parameters from 19098 reflections

b = 7.5547 (1) Å	θ = 3.1–76.5°
c = 28.6597 (4) Å	μ = 1.58 mm^{-1}
β = 101.588 (1)°	T = 150 K
V = 2773.83 (7) Å^3	Block, yellow
Z = 4	0.20 × 0.12 × 0.08 mm

Data collection

XtaLAB Synergy, Dualflex, HyPix diffractometer	5074 reflections with $I > 2\sigma(I)$
Radiation source: micro-focus sealed X-ray tube	R_{int} = 0.025
ω scans	θ_{max} = 76.7°, θ_{min} = 3.2°
Absorption correction: multi-scan *CrysAlis PRO* 1.171.41.123a (Rigaku Oxford Diffraction, 2022) Empirical absorption correction using spherical harmonics, implemented in SCALE3 ABSPACK scaling algorithm.	h = -15→16
T_{min} = 0.783, T_{max} = 1.000	k = -9→7
26402 measured reflections	l = -32→35
5448 independent reflections	

Refinement

Refinement on F^2	10 restraints
Least-squares matrix: full	Hydrogen site location: inferred from neighbouring sites
$R[F^2 > 2\sigma(F^2)]$ = 0.070	H-atom parameters constrained
$wR(F^2)$ = 0.192	$w = 1/[\sigma^2(F_o^2) + (0.1227P)^2 + 3.0177P]$ where $P = (F_o^2 + 2F_c^2)/3$
S = 1.05	$(\Delta/\sigma)_{max}$ = 0.001
5448 reflections	$\Delta\rho_{max}$ = 0.96 e Å^{-3}
395 parameters	$\Delta\rho_{min}$ = -0.48 e Å^{-3}

Special details

Geometry. All esds (except the esd in the dihedral angle between two l.s. planes) are estimated using the full covariance matrix. The cell esds are taken into account individually in the estimation of esds in distances, angles and torsion angles; correlations between esds in cell parameters are only used when they are defined by crystal symmetry. An approximate (isotropic) treatment of cell esds is used for estimating esds involving l.s. planes.

Fractional atomic coordinates and isotropic or equivalent isotropic displacement parameters (Å^2)

	x	*y*	*z*	U_{iso}*/U_{eq}	Occ. (<1)
N1	0.52659 (14)	0.5464 (3)	0.71473 (6)	0.0216 (4)	
Cl32	0.23060 (4)	0.24846 (8)	0.50400 (2)	0.0290 (2)	
N25	0.57882 (15)	0.2253 (3)	0.66104 (7)	0.0215 (4)	
N14	0.36672 (14)	0.5409 (3)	0.74327 (6)	0.0224 (4)	
H14	0.391797	0.596996	0.770033	0.027*	
N29	0.42737 (15)	0.2121 (3)	0.54211 (7)	0.0243 (4)	
O42	-0.09838 (15)	0.2999 (3)	0.67940 (8)	0.0470 (5)	
N31	0.31174 (15)	0.3144 (3)	0.59197 (7)	0.0234 (4)	
N27	0.60392 (15)	0.1698 (3)	0.58661 (7)	0.0234 (4)	
N40	-0.02301 (17)	0.2777 (3)	0.66021 (8)	0.0345 (5)	
O41	-0.02932 (17)	0.1951 (4)	0.62323 (9)	0.0623 (7)	
C24	0.48596 (17)	0.2489 (3)	0.62833 (8)	0.0179 (4)	
C15	0.42236 (16)	0.4926 (3)	0.70972 (7)	0.0201 (4)	
C30	0.33619 (18)	0.2562 (3)	0.55196 (8)	0.0216 (5)	
C28	0.50071 (18)	0.2118 (3)	0.58222 (8)	0.0218 (4)	
C9	0.60785 (17)	0.5009 (3)	0.75349 (8)	0.0223 (5)	
C23	0.38807 (17)	0.3182 (3)	0.63176 (8)	0.0207 (4)	
C10	0.60043 (18)	0.4388 (3)	0.79773 (8)	0.0243 (5)	
H10	0.534888	0.423640	0.806698	0.029*	
C16	0.36018 (17)	0.3993 (3)	0.67327 (8)	0.0220 (5)	
C17	0.25667 (17)	0.4011 (3)	0.68377 (8)	0.0228 (5)	
C7	0.57169 (18)	0.6078 (3)	0.67723 (8)	0.0230 (5)	
C22	0.26429 (17)	0.4869 (3)	0.72805 (8)	0.0240 (5)	
C26	0.64546 (18)	0.1790 (3)	0.63423 (8)	0.0241 (5)	
H26	0.716753	0.153928	0.647060	0.029*	
C6	0.5237 (2)	0.6660 (3)	0.63207 (8)	0.0270 (5)	
H6	0.449850	0.667677	0.622339	0.032*	
C1	0.70369 (18)	0.5333 (3)	0.73973 (9)	0.0258 (5)	
C5	0.5883 (2)	0.7219 (3)	0.60162 (9)	0.0316 (6)	
H5	0.557869	0.762581	0.570563	0.038*	
C2	0.68101 (18)	0.6035 (3)	0.69200 (8)	0.0258 (5)	
C33	0.65940 (18)	0.1335 (3)	0.54795 (8)	0.0273 (5)	
H33A	0.621534	0.040476	0.526990	0.033*	
H33B	0.729989	0.087807	0.561684	0.033*	
C18	0.16055 (18)	0.3334 (3)	0.66031 (8)	0.0257 (5)	
H18	0.152692	0.276845	0.630198	0.031*	

C21	0.17933 (19)	0.5065 (4)	0.75005 (9)	0.0306 (5)	
H21	0.186140	0.565130	0.779834	0.037*	
C19	0.07749 (18)	0.3528 (3)	0.68305 (9)	0.0291 (5)	
C13	0.79588 (19)	0.4879 (4)	0.77112 (9)	0.0327 (6)	
H13	0.861463	0.502481	0.762144	0.039*	
C11	0.69463 (19)	0.3990 (3)	0.82897 (9)	0.0303 (5)	
H11	0.692961	0.356035	0.859956	0.036*	
C20	0.08523 (19)	0.4380 (4)	0.72719 (9)	0.0321 (5)	
H20	0.025540	0.448214	0.741196	0.038*	
C3	0.7440 (2)	0.6608 (3)	0.66083 (10)	0.0329 (6)	
H3	0.817902	0.659463	0.670300	0.039*	
C4	0.6966 (2)	0.7192 (4)	0.61601 (10)	0.0347 (6)	
H4	0.738633	0.758232	0.594580	0.042*	
C12	0.7904 (2)	0.4210 (4)	0.81567 (10)	0.0345 (6)	
H12	0.852826	0.389821	0.837306	0.041*	
C35	0.7336 (3)	0.2600 (4)	0.48020 (11)	0.0409 (7)	
H35A	0.726211	0.360240	0.457519	0.049*	
H35B	0.706471	0.152329	0.462223	0.049*	
C34	0.6693 (2)	0.2974 (4)	0.51837 (10)	0.0359 (6)	
H34A	0.703191	0.393173	0.539572	0.043*	
H34B	0.598855	0.338570	0.502806	0.043*	
C39	1.0175 (2)	0.2988 (6)	0.40367 (12)	0.0504 (8)	
H39A	1.003501	0.361562	0.373119	0.076*	
H39B	1.021195	0.171298	0.397980	0.076*	
H39C	1.084043	0.339508	0.422786	0.076*	
C36	0.8520 (4)	0.2331 (7)	0.50303 (17)	0.0420 (10)	0.75
H36A	0.877885	0.341036	0.521212	0.050*	0.75
H36B	0.858172	0.133798	0.525970	0.050*	0.75
C37	0.9236 (3)	0.1941 (6)	0.46694 (15)	0.0396 (9)	0.75
H37A	0.995244	0.172214	0.485117	0.048*	0.75
H37B	0.899123	0.083588	0.449688	0.048*	0.75
C38	0.9286 (3)	0.3370 (6)	0.43074 (15)	0.0414 (9)	0.75
H38A	0.940833	0.452873	0.447015	0.050*	0.75
H38B	0.861144	0.342987	0.407783	0.050*	0.75
C86	0.8387 (11)	0.261 (3)	0.4883 (5)	0.0420 (10)	0.25
H86A	0.867219	0.160534	0.508939	0.050*	0.25
H86B	0.866009	0.372540	0.504340	0.050*	0.25

C87	0.8702 (9)	0.246 (2)	0.4410 (4)	0.047 (3)	0.25
H87A	0.862644	0.123227	0.429065	0.056*	0.25
H87B	0.827159	0.325171	0.417230	0.056*	0.25
C88	0.9786 (10)	0.3008 (19)	0.4501 (5)	0.0414 (9)	0.25
H88A	1.020886	0.218914	0.473253	0.050*	0.25
H88B	0.985408	0.421337	0.463944	0.050*	0.25

Atomic displacement parameters (Å^2)

	U^{11}	U^{22}	U^{33}	U^{12}	U^{13}	U^{23}
N1	0.0215 (9)	0.0251 (9)	0.0181 (9)	-0.0016 (7)	0.0042 (7)	0.0009 (7)
Cl32	0.0266 (3)	0.0396 (4)	0.0169 (3)	0.0039 (2)	-0.0049 (2)	-0.0058 (2)
N25	0.0216 (9)	0.0261 (9)	0.0162 (9)	0.0011 (7)	0.0024 (7)	-0.0001 (7)
N14	0.0226 (9)	0.0288 (10)	0.0161 (8)	-0.0029 (7)	0.0045 (7)	-0.0040 (7)
N29	0.0260 (10)	0.0309 (10)	0.0155 (9)	0.0021 (8)	0.0027 (7)	-0.0027 (8)
O42	0.0247 (9)	0.0683 (14)	0.0496 (12)	-0.0067 (9)	0.0116 (8)	-0.0005 (11)
N31	0.0233 (9)	0.0258 (10)	0.0196 (9)	0.0006 (7)	0.0007 (7)	-0.0013 (8)
N27	0.0252 (9)	0.0247 (10)	0.0212 (9)	0.0020 (7)	0.0070 (7)	-0.0012 (7)
N40	0.0236 (10)	0.0459 (13)	0.0333 (12)	-0.0028 (9)	0.0041 (9)	0.0011 (10)
O41	0.0314 (11)	0.0957 (19)	0.0586 (15)	-0.0150 (12)	0.0059 (10)	-0.0343 (15)
C24	0.0195 (10)	0.0192 (10)	0.0146 (10)	0.0000 (7)	0.0025 (8)	-0.0008 (7)
C15	0.0189 (10)	0.0233 (10)	0.0184 (10)	-0.0006 (8)	0.0049 (8)	-0.0003 (8)
C30	0.0214 (11)	0.0256 (12)	0.0156 (10)	0.0004 (8)	-0.0013 (8)	-0.0008 (8)
C28	0.0229 (10)	0.0244 (10)	0.0180 (10)	0.0019 (9)	0.0038 (8)	-0.0007 (9)
C9	0.0218 (10)	0.0254 (11)	0.0190 (10)	-0.0033 (8)	0.0029 (8)	-0.0052 (9)
C23	0.0242 (10)	0.0202 (10)	0.0174 (10)	-0.0005 (8)	0.0032 (8)	0.0006 (8)
C10	0.0251 (11)	0.0274 (11)	0.0206 (10)	-0.0048 (9)	0.0052 (8)	-0.0043 (9)
C16	0.0214 (10)	0.0256 (11)	0.0192 (10)	0.0018 (8)	0.0044 (8)	0.0007 (9)
C17	0.0214 (10)	0.0269 (11)	0.0203 (10)	0.0014 (9)	0.0045 (8)	-0.0002 (9)
C7	0.0280 (11)	0.0206 (10)	0.0226 (11)	-0.0029 (8)	0.0107 (9)	-0.0030 (9)
C22	0.0234 (11)	0.0280 (11)	0.0208 (10)	0.0006 (9)	0.0050 (8)	0.0013 (9)
C26	0.0237 (10)	0.0256 (11)	0.0226 (11)	0.0024 (9)	0.0038 (8)	0.0004 (9)
C6	0.0330 (12)	0.0243 (11)	0.0248 (11)	-0.0008 (9)	0.0084 (9)	-0.0009 (9)
C1	0.0254 (11)	0.0252 (11)	0.0279 (11)	-0.0026 (9)	0.0078 (9)	-0.0040 (9)
C5	0.0479 (15)	0.0289 (12)	0.0202 (12)	-0.0012 (11)	0.0121 (11)	0.0016 (9)
C2	0.0266 (11)	0.0253 (11)	0.0269 (11)	-0.0037 (9)	0.0086 (9)	-0.0047 (9)
C33	0.0285 (11)	0.0308 (12)	0.0247 (11)	0.0038 (10)	0.0103 (9)	-0.0023 (10)
C18	0.0242 (11)	0.0287 (12)	0.0233 (11)	0.0001 (9)	0.0027 (9)	-0.0003 (9)

C21	0.0285 (12)	0.0390 (14)	0.0259 (12)	0.0012 (10)	0.0094 (9)	-0.0038 (10)
C19	0.0202 (11)	0.0352 (13)	0.0308 (12)	-0.0019 (9)	0.0026 (9)	0.0025 (10)
C13	0.0245 (11)	0.0352 (13)	0.0373 (13)	-0.0031 (10)	0.0033 (10)	-0.0026 (11)
C11	0.0323 (12)	0.0316 (12)	0.0240 (11)	-0.0016 (10)	-0.0013 (9)	0.0014 (10)
C20	0.0243 (11)	0.0417 (14)	0.0324 (13)	0.0016 (10)	0.0110 (9)	0.0001 (11)
C3	0.0326 (12)	0.0327 (13)	0.0376 (14)	-0.0056 (10)	0.0177 (11)	-0.0042 (11)
C4	0.0451 (15)	0.0342 (13)	0.0311 (13)	-0.0069 (11)	0.0229 (12)	-0.0003 (11)
C12	0.0266 (12)	0.0360 (13)	0.0366 (13)	0.0000 (10)	-0.0042 (10)	-0.0006 (11)
C35	0.0630 (19)	0.0360 (15)	0.0278 (14)	0.0002 (12)	0.0188 (14)	0.0045 (10)
C34	0.0438 (15)	0.0347 (13)	0.0319 (14)	0.0032 (12)	0.0137 (12)	0.0032 (11)
C39	0.0327 (14)	0.084 (2)	0.0369 (16)	-0.0038 (16)	0.0121 (12)	0.0107 (16)
C36	0.049 (2)	0.054 (3)	0.028 (3)	-0.0037 (18)	0.021 (2)	0.016 (2)
C37	0.0295 (17)	0.047 (2)	0.044 (2)	0.0055 (16)	0.0120 (17)	0.0138 (18)
C38	0.037 (2)	0.050 (2)	0.040 (2)	0.0075 (18)	0.0148 (16)	0.0139 (18)
C86	0.049 (2)	0.054 (3)	0.028 (3)	-0.0037 (18)	0.021 (2)	0.016 (2)
C87	0.030 (6)	0.092 (11)	0.018 (5)	-0.008 (6)	0.001 (4)	-0.003 (5)
C88	0.037 (2)	0.050 (2)	0.040 (2)	0.0075 (18)	0.0148 (16)	0.0139 (18)

Geometric parameters (Å, º)

N1—C15	1.402 (3)	C33—H33B	0.9900
N1—C7	1.404 (3)	C18—C19	1.382 (3)
N1—C9	1.417 (3)	C18—H18	0.9500
Cl32—C30	1.743 (2)	C21—C20	1.374 (4)
N25—C26	1.320 (3)	C21—H21	0.9500
N25—C24	1.389 (3)	C19—C20	1.405 (4)
N14—C15	1.367 (3)	C13—C12	1.388 (4)
N14—C22	1.384 (3)	C13—H13	0.9500
N14—H14	0.8800	C11—C12	1.391 (4)
N29—C30	1.322 (3)	C11—H11	0.9500
N29—C28	1.341 (3)	C20—H20	0.9500
O42—N40	1.233 (3)	C3—C4	1.382 (4)
N31—C30	1.326 (3)	C3—H3	0.9500
N31—C23	1.357 (3)	C4—H4	0.9500
N27—C26	1.366 (3)	C12—H12	0.9500
N27—C28	1.367 (3)	C35—C86	1.347 (15)
N27—C33	1.467 (3)	C35—C34	1.534 (4)
N40—O41	1.218 (3)	C35—C36	1.568 (6)

N40—C19	1.460 (3)	C35—H35A	0.9900
C24—C28	1.402 (3)	C35—H35B	0.9900
C24—C23	1.405 (3)	C34—H34A	0.9900
C15—C16	1.381 (3)	C34—H34B	0.9900
C9—C10	1.373 (3)	C39—C88	1.519 (12)
C9—C1	1.409 (3)	C39—C38	1.549 (5)
C23—C16	1.449 (3)	C39—H39A	0.9800
C10—C11	1.402 (3)	C39—H39B	0.9800
C10—H10	0.9500	C39—H39C	0.9800
C16—C17	1.445 (3)	C36—C37	1.557 (6)
C17—C18	1.398 (3)	C36—H36A	0.9900
C17—C22	1.411 (3)	C36—H36B	0.9900
C7—C6	1.391 (3)	C37—C38	1.508 (5)
C7—C2	1.408 (3)	C37—H37A	0.9900
C22—C21	1.391 (3)	C37—H37B	0.9900
C26—H26	0.9500	C38—H38A	0.9900
C6—C5	1.396 (3)	C38—H38B	0.9900
C6—H6	0.9500	C86—C87	1.498 (14)
C1—C13	1.395 (3)	C86—H86A	0.9900
C1—C2	1.441 (3)	C86—H86B	0.9900
C5—C4	1.393 (4)	C87—C88	1.447 (17)
C5—H5	0.9500	C87—H87A	0.9900
C2—C3	1.400 (3)	C87—H87B	0.9900
C33—C34	1.521 (4)	C88—H88A	0.9900
C33—H33A	0.9900	C88—H88B	0.9900
C15—N1—C7	124.51 (18)	C18—C19—N40	118.0 (2)
C15—N1—C9	124.79 (19)	C20—C19—N40	118.5 (2)
C7—N1—C9	108.42 (18)	C12—C13—C1	119.0 (2)
C26—N25—C24	103.37 (18)	C12—C13—H13	120.5
C15—N14—C22	108.53 (18)	C1—C13—H13	120.5
C15—N14—H14	125.7	C12—C11—C10	121.6 (2)
C22—N14—H14	125.7	C12—C11—H11	119.2
C30—N29—C28	109.74 (19)	C10—C11—H11	119.2
C30—N31—C23	117.99 (19)	C21—C20—C19	119.8 (2)
C26—N27—C28	105.51 (18)	C21—C20—H20	120.1
C26—N27—C33	127.33 (19)	C19—C20—H20	120.1

C28—N27—C33	127.06 (19)	C4—C3—C2	118.7 (2)
O41—N40—O42	122.5 (2)	C4—C3—H3	120.6
O41—N40—C19	119.1 (2)	C2—C3—H3	120.6
O42—N40—C19	118.4 (2)	C3—C4—C5	121.3 (2)
N25—C24—C28	110.10 (19)	C3—C4—H4	119.3
N25—C24—C23	133.3 (2)	C5—C4—H4	119.3
C28—C24—C23	116.1 (2)	C13—C12—C11	120.7 (2)
N14—C15—C16	110.65 (19)	C13—C12—H12	119.6
N14—C15—N1	119.63 (19)	C11—C12—H12	119.6
C16—C15—N1	129.7 (2)	C86—C35—C34	124.3 (7)
N29—C30—N31	130.8 (2)	C34—C35—C36	111.3 (3)
N29—C30—Cl32	115.39 (17)	C34—C35—H35A	109.4
N31—C30—Cl32	113.75 (17)	C36—C35—H35A	109.4
N29—C28—N27	127.0 (2)	C34—C35—H35B	109.4
N29—C28—C24	126.7 (2)	C36—C35—H35B	109.4
N27—C28—C24	106.25 (19)	H35A—C35—H35B	108.0
C10—C9—C1	123.3 (2)	C33—C34—C35	111.6 (2)
C10—C9—N1	128.7 (2)	C33—C34—H34A	109.3
C1—C9—N1	107.92 (19)	C35—C34—H34A	109.3
N31—C23—C24	117.4 (2)	C33—C34—H34B	109.3
N31—C23—C16	115.78 (19)	C35—C34—H34B	109.3
C24—C23—C16	126.8 (2)	H34A—C34—H34B	108.0
C9—C10—C11	116.5 (2)	C38—C39—H39A	109.5
C9—C10—H10	121.7	C38—C39—H39B	109.5
C11—C10—H10	121.7	H39A—C39—H39B	109.5
C15—C16—C17	105.77 (19)	C38—C39—H39C	109.5
C15—C16—C23	129.0 (2)	H39A—C39—H39C	109.5
C17—C16—C23	125.2 (2)	H39B—C39—H39C	109.5
C18—C17—C22	119.7 (2)	C37—C36—C35	115.0 (4)
C18—C17—C16	133.3 (2)	C37—C36—H36A	108.5
C22—C17—C16	106.96 (19)	C35—C36—H36A	108.5
C6—C7—N1	129.5 (2)	C37—C36—H36B	108.5
C6—C7—C2	121.9 (2)	C35—C36—H36B	108.5
N1—C7—C2	108.6 (2)	H36A—C36—H36B	107.5
N14—C22—C21	129.5 (2)	C38—C37—C36	115.9 (3)
N14—C22—C17	107.93 (19)	C38—C37—H37A	108.3
C21—C22—C17	122.5 (2)	C36—C37—H37A	108.3

N25—C26—N27	114.8 (2)	C38—C37—H37B	108.3
N25—C26—H26	122.6	C36—C37—H37B	108.3
N27—C26—H26	122.6	H37A—C37—H37B	107.4
C7—C6—C5	117.4 (2)	C37—C38—C39	110.6 (3)
C7—C6—H6	121.3	C37—C38—H38A	109.5
C5—C6—H6	121.3	C39—C38—H38A	109.5
C13—C1—C9	118.6 (2)	C37—C38—H38B	109.5
C13—C1—C2	133.6 (2)	C39—C38—H38B	109.5
C9—C1—C2	107.7 (2)	H38A—C38—H38B	108.1
C4—C5—C6	121.1 (2)	C35—C86—C87	107.5 (11)
C4—C5—H5	119.4	C35—C86—H86A	110.2
C6—C5—H5	119.4	C87—C86—H86A	110.2
C3—C2—C7	119.6 (2)	C35—C86—H86B	110.2
C3—C2—C1	133.2 (2)	C87—C86—H86B	110.2
C7—C2—C1	107.3 (2)	H86A—C86—H86B	108.5
N27—C33—C34	112.3 (2)	C88—C87—C86	104.6 (11)
N27—C33—H33A	109.1	C88—C87—H87A	110.8
C34—C33—H33A	109.1	C86—C87—H87A	110.8
N27—C33—H33B	109.1	C88—C87—H87B	110.8
C34—C33—H33B	109.1	C86—C87—H87B	110.8
H33A—C33—H33B	107.9	H87A—C87—H87B	108.9
C19—C18—C17	116.8 (2)	C87—C88—C39	109.1 (10)
C19—C18—H18	121.6	C87—C88—H88A	109.9
C17—C18—H18	121.6	C39—C88—H88A	109.9
C20—C21—C22	117.7 (2)	C87—C88—H88B	109.9
C20—C21—H21	121.2	C39—C88—H88B	109.9
C22—C21—H21	121.2	H88A—C88—H88B	108.3
C18—C19—C20	123.5 (2)		

Document origin: *publCIF* [Westrip, S. P. (2010). *J. Apply. Cryst.*, **43**, 920-925].

3-(2-Chloro-9-heptyl-9*H*-purin-6-yl)-*N*-(4-nitrophenyl)-1-phenyl-1*H*-indol-2-amine (8b)

CCDC 2391859

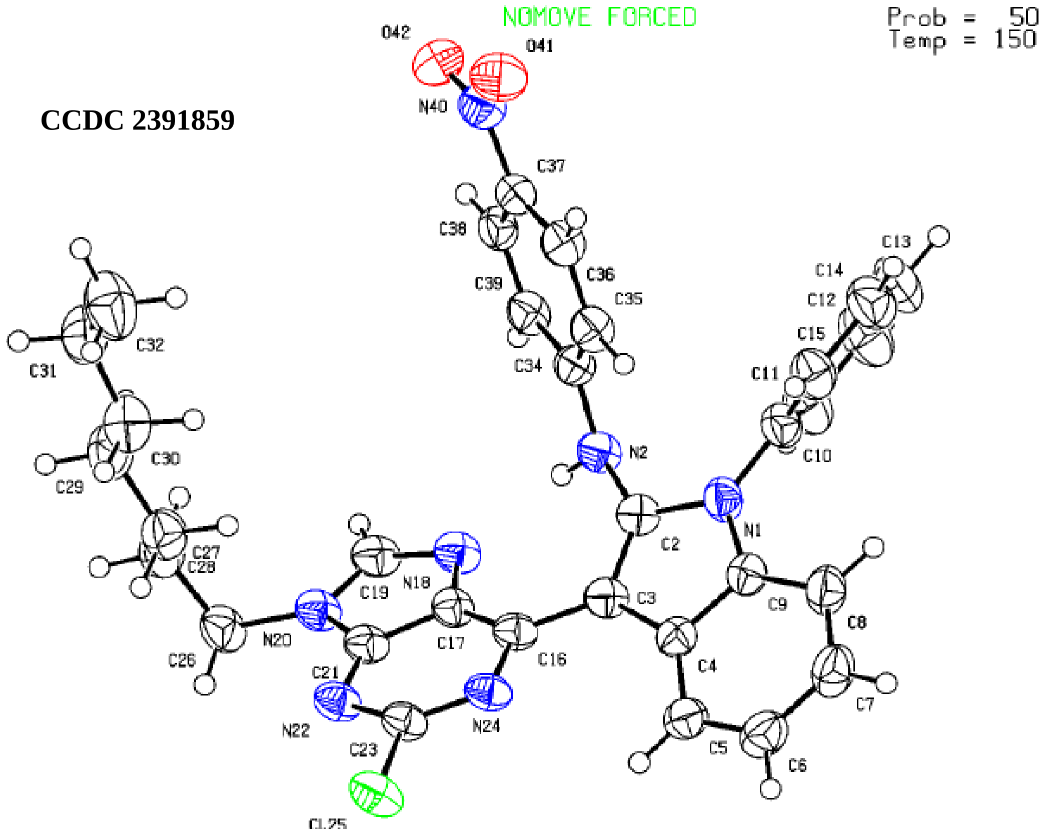

Figure S4. ORTEP plot for **8b** with the atom numbering scheme.

Crystal data, data collection and structure refinement details are summarized in Table S4.

Table S4. Experimental details

Crystal data	
Chemical formula	$C_{32}H_{30}ClN_7O_2$
M_r	580.08
Crystal system, space group	Monoclinic, $P2_1/c$
Temperature (K)	150
a, *b*, *c* (Å)	14.6251 (8), 18.5732 (9), 11.5394 (5)
β (°)	107.436 (5)
V (Å^3)	2990.5 (3)
Z	4
Radiation type	Cu $K\alpha$
μ (mm^{-1})	1.47
Crystal size (mm)	0.20 × 0.15 × 0.05

Data collection	
Diffractometer	XtaLAB Synergy, Dualflex, HyPix
Absorption correction	Multi-scan<br>*CrysAlis PRO* 1.171.40.71a (Rigaku Oxford Diffraction, 2020) Empirical absorption correction using spherical harmonics, implemented in SCALE3 ABSPACK scaling algorithm.
T_{min}, T_{max}	0.435, 1.000
No. of measured, independent and observed [$I > 2\sigma(I)$] reflections	20321, 5563, 4448
R_{int}	0.058
$(\sin \theta/\lambda)_{max}$ (Å^{-1})	0.631
Refinement	
$R[F^2 > 2\sigma(F^2)]$, $wR(F^2)$, S	0.086, 0.201, 1.13
No. of reflections	5563
No. of parameters	383
H-atom treatment	H atoms treated by a mixture of independent and constrained refinement
$\Delta\rho_{max}$, $\Delta\rho_{min}$ (e Å^{-3})	0.39, -0.35

Computer programs: *CrysAlis PRO* 1.171.42.93a (Rigaku OD, 2023), SHELXT 2014/4 (Sheldrick, 2014), *SHELXL2018*/3 (Sheldrick, 2018).

References

Document origin: *publCIF* [Westrip, S. P. (2010). *J. Apply. Cryst.*, **43**, 920-925].

Computing details

Data collection: *CrysAlis PRO* 1.171.42.93a (Rigaku OD, 2023); cell refinement: *CrysAlis PRO* 1.171.42.93a (Rigaku OD, 2023); data reduction: *CrysAlis PRO* 1.171.42.93a (Rigaku OD, 2023); program(s) used to solve structure: SHELXT 2014/4 (Sheldrick, 2014); program(s) used to refine structure: *SHELXL2018*/3 (Sheldrick, 2018).

Crystal data

$C_{32}H_{30}ClN_7O_2$	$F(000) = 1216$
$M_r = 580.08$	$D_x = 1.288$ Mg m^{-3}
Monoclinic, $P2_1/c$	Cu $K\alpha$ radiation, $\lambda = 1.54184$ Å
$a = 14.6251$ (8) Å	Cell parameters from 10451 reflections
$b = 18.5732$ (9) Å	$\theta = 3.2–74.1°$

c = 11.5394 (5) Å	μ = 1.47 mm^{-1}
β = 107.436 (5)°	T = 150 K
V = 2990.5 (3) Å^3	Plate, yellow
Z = 4	0.20 × 0.15 × 0.05 mm

Data collection

XtaLAB Synergy, Dualflex, HyPix diffractometer	4448 reflections with $I > 2\sigma(I)$
Radiation source: micro-focus sealed X-ray tube	R_{int} = 0.058
ω scans	θ_{max} = 76.6°, θ_{min} = 4.7°
Absorption correction: multi-scan *CrysAlis PRO* 1.171.40.71a (Rigaku Oxford Diffraction, 2020) Empirical absorption correction using spherical harmonics, implemented in SCALE3 ABSPACK scaling algorithm.	H = -17→18
T_{min} = 0.435, T_{max} = 1.000	k = -23→21
20321 measured reflections	l = -13→14
5563 independent reflections	

Refinement

Refinement on F^2	0 restraints
Least-squares matrix: full	Hydrogen site location: mixed
$R[F^2 > 2\sigma(F^2)]$ = 0.086	H atoms treated by a mixture of independent and constrained refinement
$wR(F^2)$ = 0.201	$w = 1/[\sigma^2(F_o^2) + (0.0412P)^2 + 8.3055P]$ where $P = (F_o^2 + 2F_c^2)/3$
S = 1.13	$(\Delta/\sigma)_{max}$ < 0.001
5563 reflections	$\Delta\rho_{max}$ = 0.39 e Å^{-3}
383 parameters	$\Delta\rho_{min}$ = -0.35 e Å^{-3}

Special details

Geometry. All esds (except the esd in the dihedral angle between two l.s. planes) are estimated using the full covariance matrix. The cell esds are taken into account individually in the estimation of esds in distances, angles and torsion angles; correlations between esds in cell parameters are only used when they are defined by crystal symmetry. An approximate (isotropic) treatment of cell esds is used for estimating esds involving l.s. planes.

Fractional atomic coordinates and isotropic or equivalent isotropic displacement parameters (Å^2)

	x	*y*	*z*	U_{iso}*/U_{eq}
Cl25	0.41582 (9)	0.04434 (6)	0.34156 (10)	0.0531 (3)
O42	0.5494 (2)	0.62989 (17)	0.5817 (3)	0.0582 (8)
O41	0.5597 (3)	0.57373 (19)	0.7496 (3)	0.0616 (9)
N24	0.5599 (3)	0.13166 (17)	0.4015 (3)	0.0393 (8)
N20	0.4634 (3)	0.26733 (19)	0.1213 (3)	0.0415 (8)
N18	0.6069 (3)	0.29754 (19)	0.2505 (3)	0.0424 (8)
N1	0.8119 (2)	0.26047 (19)	0.6418 (3)	0.0421 (8)
N2	0.7232 (3)	0.33620 (18)	0.4782 (3)	0.0407 (8)
H1N2	0.700 (4)	0.335 (3)	0.400 (5)	0.061*
N22	0.4318 (3)	0.15883 (19)	0.2196 (3)	0.0436 (8)
N40	0.5719 (3)	0.5767 (2)	0.6485 (3)	0.0499 (9)
C17	0.5736 (3)	0.2357 (2)	0.2915 (3)	0.0365 (9)
C21	0.4849 (3)	0.2163 (2)	0.2109 (3)	0.0384 (9)
C4	0.7466 (3)	0.1503 (2)	0.5817 (4)	0.0381 (9)
C39	0.6674 (3)	0.4577 (2)	0.4551 (3)	0.0409 (9)
H39	0.679015	0.458648	0.378403	0.049*
C37	0.6146 (3)	0.5150 (2)	0.6072 (4)	0.0422 (9)
C2	0.7407 (3)	0.2692 (2)	0.5320 (3)	0.0385 (9)
C16	0.6109 (3)	0.1917 (2)	0.3925 (3)	0.0379 (9)
C34	0.6894 (3)	0.3956 (2)	0.5260 (3)	0.0382 (9)
C23	0.4773 (3)	0.1203 (2)	0.3173 (3)	0.0410 (9)
C38	0.6295 (3)	0.5173 (2)	0.4932 (4)	0.0425 (10)
H38	0.613678	0.558973	0.443549	0.051*
C3	0.6982 (3)	0.2034 (2)	0.4919 (3)	0.0388 (9)
C9	0.8159 (3)	0.1876 (2)	0.6724 (4)	0.0404 (9)
C35	0.6742 (3)	0.3949 (2)	0.6408 (3)	0.0416 (9)
H35	0.688994	0.353084	0.690394	0.050*
C36	0.6378 (3)	0.4552 (2)	0.6809 (4)	0.0438 (10)
H36	0.628614	0.455464	0.759006	0.053*
C5	0.7385 (3)	0.0759 (2)	0.5917 (4)	0.0439 (10)
H5	0.693027	0.049410	0.530519	0.053*
C19	0.5388 (3)	0.3144 (2)	0.1501 (3)	0.0436 (10)
H19	0.541940	0.355343	0.102135	0.052*
C10	0.8771 (3)	0.3137 (2)	0.7087 (4)	0.0455 (10)
C8	0.8769 (3)	0.1532 (3)	0.7733 (4)	0.0492 (11)

H8	0.923719	0.179249	0.833677	0.059*
C26	0.3758 (3)	0.2702 (3)	0.0174 (4)	0.0492 (11)
H26A	0.345748	0.221903	0.004915	0.059*
H26B	0.393211	0.282748	-0.056699	0.059*
C6	0.7982 (3)	0.0415 (3)	0.6928 (4)	0.0528 (11)
H6	0.792491	-0.008978	0.701537	0.063*
C28	0.2680 (3)	0.3081 (3)	0.1441 (4)	0.0535 (12)
H28A	0.232146	0.262147	0.129133	0.064*
H28B	0.324020	0.301723	0.216997	0.064*
C7	0.8665 (3)	0.0799 (3)	0.7822 (4)	0.0547 (12)
H7	0.906700	0.054865	0.850379	0.066*
C27	0.3037 (4)	0.3247 (3)	0.0351 (4)	0.0541 (12)
H27A	0.248105	0.326050	-0.039306	0.065*
H27B	0.333613	0.373029	0.046024	0.065*
C15	0.8814 (3)	0.3272 (3)	0.8281 (4)	0.0531 (12)
H15	0.840524	0.302080	0.864582	0.064*
C11	0.9360 (4)	0.3497 (3)	0.6551 (4)	0.0580 (13)
H11	0.932779	0.340063	0.573153	0.070*
C29	0.2037 (4)	0.3667 (3)	0.1693 (4)	0.0611 (13)
H29A	0.142141	0.366959	0.103281	0.073*
H29B	0.234633	0.414141	0.169312	0.073*
C30	0.1836 (4)	0.3561 (3)	0.2918 (4)	0.0606 (13)
H30A	0.144968	0.311848	0.287381	0.073*
H30B	0.245239	0.348919	0.355987	0.073*
C14	0.9462 (4)	0.3779 (3)	0.8942 (5)	0.0624 (13)
H14	0.949479	0.387754	0.976172	0.075*
C13	1.0057 (4)	0.4140 (3)	0.8413 (5)	0.0698 (15)
H13	1.050460	0.448169	0.886923	0.084*
C12	1.0000 (4)	0.4003 (3)	0.7221 (5)	0.0717 (16)
H12	1.040343	0.425753	0.685321	0.086*
C31	0.1311 (4)	0.4185 (3)	0.3273 (5)	0.0666 (14)
H31A	0.066646	0.422842	0.267681	0.080*
H31B	0.166447	0.463665	0.324516	0.080*
C32	0.1207 (4)	0.4093 (4)	0.4550 (5)	0.0825 (19)
H32A	0.085566	0.364807	0.458181	0.124*
H32B	0.085522	0.450429	0.473687	0.124*
H32C	0.184427	0.406858	0.514773	0.124*

Atomic displacement parameters (Å^2)

	U^{11}	U^{22}	U^{33}	U^{12}	U^{13}	U^{23}
Cl25	0.0695 (7)	0.0465 (6)	0.0416 (5)	-0.0171 (5)	0.0142 (5)	-0.0042 (5)
O42	0.063 (2)	0.0438 (18)	0.066 (2)	0.0075 (16)	0.0164 (17)	0.0036 (16)
O41	0.075 (2)	0.064 (2)	0.0519 (19)	-0.0001 (18)	0.0273 (17)	-0.0101 (16)
N24	0.053 (2)	0.0356 (18)	0.0315 (16)	-0.0040 (15)	0.0160 (15)	-0.0054 (14)
N20	0.055 (2)	0.0429 (19)	0.0262 (16)	-0.0011 (17)	0.0114 (15)	-0.0013 (14)
N18	0.055 (2)	0.044 (2)	0.0286 (16)	-0.0016 (16)	0.0142 (15)	-0.0002 (15)
N1	0.0390 (19)	0.043 (2)	0.0386 (18)	-0.0009 (15)	0.0032 (15)	0.0039 (15)
N2	0.052 (2)	0.0363 (18)	0.0308 (16)	-0.0021 (15)	0.0086 (15)	0.0029 (14)
N22	0.056 (2)	0.042 (2)	0.0327 (17)	-0.0037 (16)	0.0129 (16)	-0.0064 (15)
N40	0.052 (2)	0.047 (2)	0.050 (2)	-0.0036 (18)	0.0139 (18)	-0.0022 (18)
C17	0.043 (2)	0.040 (2)	0.0276 (18)	0.0000 (17)	0.0117 (16)	-0.0018 (16)
C21	0.051 (2)	0.039 (2)	0.0266 (18)	-0.0004 (18)	0.0142 (17)	-0.0039 (16)
C4	0.039 (2)	0.037 (2)	0.038 (2)	0.0021 (17)	0.0117 (17)	0.0013 (17)
C39	0.049 (2)	0.042 (2)	0.0291 (19)	-0.0039 (19)	0.0075 (17)	0.0048 (17)
C37	0.040 (2)	0.039 (2)	0.043 (2)	-0.0022 (18)	0.0056 (18)	-0.0027 (18)
C2	0.044 (2)	0.038 (2)	0.035 (2)	-0.0013 (18)	0.0153 (17)	0.0018 (17)
C16	0.047 (2)	0.037 (2)	0.0326 (19)	-0.0004 (18)	0.0153 (17)	-0.0054 (16)
C34	0.042 (2)	0.036 (2)	0.035 (2)	-0.0009 (17)	0.0084 (17)	0.0025 (17)
C23	0.051 (3)	0.039 (2)	0.033 (2)	-0.0044 (19)	0.0134 (18)	-0.0061 (17)
C38	0.043 (2)	0.042 (2)	0.037 (2)	-0.0015 (18)	0.0036 (18)	0.0025 (18)
C3	0.049 (2)	0.037 (2)	0.0314 (19)	-0.0001 (18)	0.0137 (17)	0.0022 (16)
C9	0.039 (2)	0.041 (2)	0.039 (2)	0.0022 (18)	0.0091 (17)	0.0022 (18)
C35	0.048 (2)	0.042 (2)	0.033 (2)	-0.0033 (19)	0.0097 (18)	0.0094 (17)
C36	0.048 (2)	0.049 (3)	0.035 (2)	-0.001 (2)	0.0132 (18)	0.0021 (18)
C5	0.044 (2)	0.038 (2)	0.051 (2)	0.0014 (18)	0.016 (2)	-0.0005 (19)
C19	0.063 (3)	0.042 (2)	0.0288 (19)	0.000 (2)	0.0168 (19)	-0.0004 (17)
C10	0.047 (2)	0.043 (2)	0.039 (2)	-0.0002 (19)	0.0011 (18)	0.0013 (19)
C8	0.041 (2)	0.051 (3)	0.050 (3)	0.003 (2)	0.0049 (19)	0.007 (2)
C26	0.056 (3)	0.056 (3)	0.032 (2)	-0.002 (2)	0.0072 (19)	-0.0019 (19)
C6	0.052 (3)	0.042 (2)	0.065 (3)	0.005 (2)	0.018 (2)	0.008 (2)
C28	0.051 (3)	0.058 (3)	0.048 (2)	-0.001 (2)	0.008 (2)	0.004 (2)
C7	0.048 (3)	0.053 (3)	0.058 (3)	0.007 (2)	0.008 (2)	0.016 (2)
C27	0.058 (3)	0.061 (3)	0.037 (2)	0.004 (2)	0.006 (2)	0.004 (2)
C15	0.054 (3)	0.061 (3)	0.040 (2)	-0.001 (2)	0.007 (2)	0.002 (2)

C11	0.060 (3)	0.069 (3)	0.043 (2)	-0.012 (3)	0.012 (2)	0.002 (2)
C29	0.053 (3)	0.081 (4)	0.046 (3)	0.006 (3)	0.010 (2)	0.003 (2)
C30	0.052 (3)	0.073 (3)	0.055 (3)	0.000 (3)	0.013 (2)	0.005 (3)
C14	0.066 (3)	0.067 (3)	0.046 (3)	-0.004 (3)	0.004 (2)	-0.003 (2)
C13	0.064 (3)	0.070 (4)	0.060 (3)	-0.020 (3)	-0.006 (3)	-0.005 (3)
C12	0.073 (4)	0.078 (4)	0.057 (3)	-0.030 (3)	0.010 (3)	0.007 (3)
C31	0.050 (3)	0.091 (4)	0.056 (3)	0.005 (3)	0.011 (2)	-0.006 (3)
C32	0.060 (4)	0.131 (6)	0.057 (3)	-0.004 (4)	0.018 (3)	-0.013 (3)

Geometric parameters (Å, º)

Cl25—C23	1.739 (4)	C19—H19	0.9500
O42—N40	1.235 (5)	C10—C11	1.375 (7)
O41—N40	1.233 (5)	C10—C15	1.383 (6)
N24—C23	1.321 (5)	C8—C7	1.378 (6)
N24—C16	1.362 (5)	C8—H8	0.9500
N20—C19	1.368 (6)	C26—C27	1.519 (7)
N20—C21	1.369 (5)	C26—H26A	0.9900
N20—C26	1.470 (5)	C26—H26B	0.9900
N18—C19	1.319 (5)	C6—C7	1.397 (7)
N18—C17	1.385 (5)	C6—H6	0.9500
N1—C2	1.388 (5)	C28—C29	1.523 (7)
N1—C9	1.395 (5)	C28—C27	1.530 (7)
N1—C10	1.429 (5)	C28—H28A	0.9900
N2—C2	1.380 (5)	C28—H28B	0.9900
N2—C34	1.389 (5)	C7—H7	0.9500
N2—H1N2	0.87 (5)	C27—H27A	0.9900
N22—C23	1.333 (5)	C27—H27B	0.9900
N22—C21	1.341 (5)	C15—C14	1.392 (7)
N40—C37	1.452 (6)	C15—H15	0.9500
C17—C16	1.394 (5)	C11—C12	1.387 (7)
C17—C21	1.399 (6)	C11—H11	0.9500
C4—C5	1.393 (6)	C29—C30	1.540 (7)
C4—C9	1.403 (6)	C29—H29A	0.9900
C4—C3	1.452 (5)	C29—H29B	0.9900
C39—C38	1.368 (6)	C30—C31	1.513 (7)
C39—C34	1.395 (5)	C30—H30A	0.9900
C39—H39	0.9500	C30—H30B	0.9900

C37—C36	1.379 (6)	C14—C13	1.376 (8)
C37—C38	1.397 (6)	C14—H14	0.9500
C2—C3	1.387 (6)	C13—C12	1.377 (8)
C16—C3	1.454 (6)	C13—H13	0.9500
C34—C35	1.408 (5)	C12—H12	0.9500
C38—H38	0.9500	C31—C32	1.536 (7)
C9—C8	1.392 (6)	C31—H31A	0.9900
C35—C36	1.378 (6)	C31—H31B	0.9900
C35—H35	0.9500	C32—H32A	0.9800
C36—H36	0.9500	C32—H32B	0.9800
C5—C6	1.386 (6)	C32—H32C	0.9800
C5—H5	0.9500		
C23—N24—C16	118.5 (3)	C7—C8—C9	117.3 (4)
C19—N20—C21	105.9 (3)	C7—C8—H8	121.3
C19—N20—C26	128.1 (4)	C9—C8—H8	121.3
C21—N20—C26	126.0 (4)	N20—C26—C27	112.6 (4)
C19—N18—C17	104.3 (4)	N20—C26—H26A	109.1
C2—N1—C9	107.9 (3)	C27—C26—H26A	109.1
C2—N1—C10	127.5 (3)	N20—C26—H26B	109.1
C9—N1—C10	124.3 (3)	C27—C26—H26B	109.1
C2—N2—C34	125.3 (3)	H26A—C26—H26B	107.8
C2—N2—H1N2	114 (3)	C5—C6—C7	121.2 (4)
C34—N2—H1N2	111 (3)	C5—C6—H6	119.4
C23—N22—C21	109.7 (4)	C7—C6—H6	119.4
O41—N40—O42	122.7 (4)	C29—C28—C27	113.3 (4)
O41—N40—C37	118.2 (4)	C29—C28—H28A	108.9
O42—N40—C37	119.1 (4)	C27—C28—H28A	108.9
N18—C17—C16	133.1 (4)	C29—C28—H28B	108.9
N18—C17—C21	109.7 (3)	C27—C28—H28B	108.9
C16—C17—C21	117.2 (4)	H28A—C28—H28B	107.7
N22—C21—N20	127.0 (4)	C8—C7—C6	121.3 (4)
N22—C21—C17	126.8 (4)	C8—C7—H7	119.4
N20—C21—C17	106.3 (4)	C6—C7—H7	119.4
C5—C4—C9	119.2 (4)	C26—C27—C28	113.4 (4)
C5—C4—C3	134.2 (4)	C26—C27—H27A	108.9
C9—C4—C3	106.6 (3)	C28—C27—H27A	108.9

C38—C39—C34	121.6 (4)	C26—C27—H27B	108.9
C38—C39—H39	119.2	C28—C27—H27B	108.9
C34—C39—H39	119.2	H27A—C27—H27B	107.7
C36—C37—C38	121.7 (4)	C10—C15—C14	119.3 (5)
C36—C37—N40	119.0 (4)	C10—C15—H15	120.4
C38—C37—N40	119.3 (4)	C14—C15—H15	120.4
N2—C2—C3	129.8 (4)	C10—C11—C12	119.2 (5)
N2—C2—N1	120.3 (4)	C10—C11—H11	120.4
C3—C2—N1	109.9 (3)	C12—C11—H11	120.4
N24—C16—C17	117.2 (4)	C28—C29—C30	112.5 (4)
N24—C16—C3	115.7 (3)	C28—C29—H29A	109.1
C17—C16—C3	127.1 (4)	C30—C29—H29A	109.1
N2—C34—C39	118.1 (4)	C28—C29—H29B	109.1
N2—C34—C35	122.8 (4)	C30—C29—H29B	109.1
C39—C34—C35	119.1 (4)	H29A—C29—H29B	107.8
N24—C23—N22	130.6 (4)	C31—C30—C29	113.7 (4)
N24—C23—Cl25	114.5 (3)	C31—C30—H30A	108.8
N22—C23—Cl25	114.9 (3)	C29—C30—H30A	108.8
C39—C38—C37	118.1 (4)	C31—C30—H30B	108.8
C39—C38—H38	120.9	C29—C30—H30B	108.8
C37—C38—H38	120.9	H30A—C30—H30B	107.7
C2—C3—C4	106.7 (4)	C13—C14—C15	120.2 (5)
C2—C3—C16	126.5 (4)	C13—C14—H14	119.9
C4—C3—C16	125.9 (4)	C15—C14—H14	119.9
C8—C9—N1	128.7 (4)	C14—C13—C12	119.7 (5)
C8—C9—C4	122.4 (4)	C14—C13—H13	120.1
N1—C9—C4	109.0 (3)	C12—C13—H13	120.1
C36—C35—C34	119.6 (4)	C13—C12—C11	120.7 (5)
C36—C35—H35	120.2	C13—C12—H12	119.6
C34—C35—H35	120.2	C11—C12—H12	119.6
C35—C36—C37	119.8 (4)	C30—C31—C32	112.3 (5)
C35—C36—H36	120.1	C30—C31—H31A	109.2
C37—C36—H36	120.1	C32—C31—H31A	109.2
C6—C5—C4	118.6 (4)	C30—C31—H31B	109.2
C6—C5—H5	120.7	C32—C31—H31B	109.2
C4—C5—H5	120.7	H31A—C31—H31B	107.9
N18—C19—N20	113.8 (4)	C31—C32—H32A	109.5

N18—C19—H19	123.1	C31—C32—H32B	109.5
N20—C19—H19	123.1	H32A—C32—H32B	109.5
C11—C10—C15	120.8 (4)	C31—C32—H32C	109.5
C11—C10—N1	120.0 (4)	H32A—C32—H32C	109.5
C15—C10—N1	119.2 (4)	H32B—C32—H32C	109.5

Document origin: *publCIF* [Westrip, S. P. (2010). *J. Apply. Cryst.*, **43**, 920-925].

9. References

32. Burcevs, A.; Sebris, A.; Traskovskis, K.; Chu, H.W.; Chang, H.T.; Jovaišaitė, J.; Juršėnas, S.; Turks, M.; Novosjolova, I. Synthesis of Fluorescent C–C Bonded Triazole-Purine Conjugates. *J. Fluoresc.* **2024**, *34*, 1091–1097, doi:10.1007/s10895-023-03337-6.
47. Barral, K.; Moorhouse, A.D.; Moses, J.E. Efficient Conversion of Aromatic Amines into Azides: A One-Pot Synthesis of Triazole Linkages. *Org. Lett.* **2007**, *9*, 1809–1811, doi: 10.1021/ol070527h.
68. Das, J.; Patil, S.N.; Awasthi, R.; Narasimhulu, C.P.; Trehan, S. An Easy Access to Aryl Azides from Aryl Amines under Neutral Conditions. *Synthesis (Stuttg).* **2005**, 1801–1806, doi:10.1055/s-2005-869974.
69. Westrip, S.P. *publCIF*: software for editing, validating and formatting crystallographic information files. *J. Appl. Cryst.* **2010**, *43*, 920–925. doi: 10.1107/S0021889810022120.
